# Exploring the Multicomponent Synergy Mechanism of Yinzhihuang Granule in Inhibiting Inflammation-Cancer Transformation of Hepar Based on Integrated Bioinformatics and Network Pharmacology

**DOI:** 10.1155/2022/6213865

**Published:** 2022-03-18

**Authors:** Jingyuan Zhang, Zhihong Huang, Xinkui Liu, Chao Wu, Wei Zhou, Peizhi Ye, Antony Stalin, Shan Lu, Yingying Tan, Zhishan Wu, Xiaotian Fan, Xiaomeng Zhang, Miaomiao Wang, Bingbing Li, Guoliang Cheng, Yanfang Mou, Jiarui Wu

**Affiliations:** ^1^Department of Clinical Chinese Pharmacy, School of Chinese Materia Medica, Beijing University of Chinese Medicine, No. 11 of North Three-Ring East Road, Chao Yang District, Beijing, China; ^2^National Clinical Research Center for Cancer, Chinese Medicine Department of the Caner Hospital of the Chinese Academy of Medical Sciences and Peking Union Medical College, Beijing, China; ^3^State Key Laboratory of Subtropical Silviculture, Department of Traditional Chinese Medicine, Zhejiang University, Hangzhou, China; ^4^State Key Laboratory of Generic Manufacture Technology of Chinese Traditional Medicine, Linyi, China

## Abstract

**Background:**

The Chinese patent drug Yinzhihuang granule (YZHG) is used to treat hepatitis B. This research is aimed at exploring the multicomponent synergistic mechanism of YZHG in the treatment of inflammation-cancer transformation of hepar and at providing new evidence and insights for its clinical application.

**Methods:**

To retrieve the components and targets of Yinzhihuang granules. The differentially expressed genes (DEGs) of hepar inflammation-cancer transformation were obtained from TTD, PharmGKB, and GEO databases. Construct the compound-prediction target network and the key module network using Cytoscape 3.7.1.

**Results:**

The results show that hepatitis B and hepatitis C shared a common target, MMP2. CDK1 and TOP2A may play an important role in the treatment with YZHG in hepatitis B inflammatory cancer transformation. KEGG pathway enrichment showed that key genes of modules 1, 2, and 4 were mainly enriched in the progesterone-mediated oocyte maturation signaling pathway and oocyte meiosis signaling pathway.

**Conclusion:**

The multicomponent, multitarget, and multichannel pharmacological benefits of YZHG in the therapy of inflammation-cancer transition of hepar are directly demonstrated by network pharmacology, providing a scientific basis for its mechanism.

## 1. Introduction

Chronic viral hepatitis affects half of the people in the world, which may lead to cirrhosis and liver cancer [1]. Liver cancer is the third leading cause of cancer-related mortality, and hepatocellular carcinoma (HCC) accounts for 90% of all primary liver cancers [2–4]. Solid tumors such as HCC are very complex and have heterogeneous tumor genome maps, leading to complexity in diagnosis and management. Chronic hepatitis B virus (HBV) and hepatitis C virus (HCV) infection is the most significant cause of HCC. Common mechanisms of HBV- and HCV-induced hepatocarcinogenesis include sustained liver inflammation, impaired antiviral immune response, immune and viral protein-mediated oxidative stress, and regulation of cellular signaling pathways by viral proteins. Promotion of genomic instability through DNA integration is a feature of HBV infection, and metabolic reprogramming leading to steatosis is driven by HCV infection [5–7]. Inflammation-cancer transformation is a dynamic process of disease occurrence, development, and transformation that represents a stage in developing and transforming many diseases. Chronic hepatitis B- (hepatitis C) cirrhosis-hepatocellular carcinoma is a common rule of disease development and transformation in the clinic. Researchers consider this transformation process a typical transformation process of inflammation and cancer [8].

**Figure 1:**
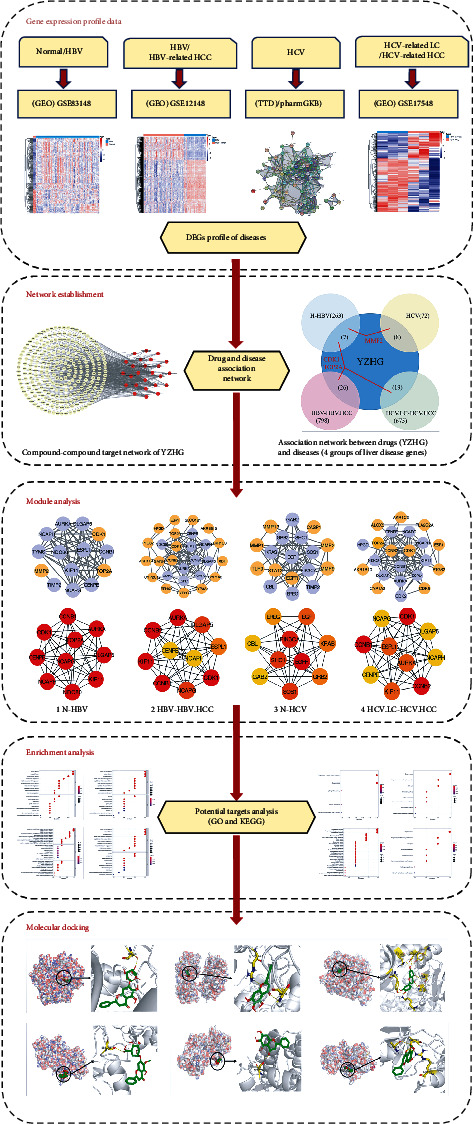
Network pharmacology analysis flow chart of YZHG in the treatment of advanced hepatitis B, hepatitis C, and related hepatocellular carcinoma. Firstly, the datasets of four different disease stages are retrieved in GEO (the missing part is replaced by the disease database). Analyze the differential genes of each dataset. Combine the predicted targets of Yinzhihuang granule components with four groups of disease differential genes, and get the potential therapeutic targets of four groups of diseases at different stages. Four groups of key genes were analyzed by the protein interaction network and enrichment. The key targets are verified by molecular docking.

YZHG is a preparation of traditional Chinese medicine made from the extracts of *Artemisia scoparza* Waldst. et Kit. or *Artemisia capillaris* Thunb. (Yinchen, YC), *Gardenia jasminoides* Ellis (Zhizi, ZZ), *Scutellaria baicalensis* Georgi (Huangqin, HQ), and *Lonicera japonica* Thunb. (Jinyinhua, JYH). It has the effects of clearing heat and detoxifying, normalizing the gallbladder to cure jaundice. It is widely used in neonatal jaundice, viral hepatitis, drug-induced hepatitis, intrahepatic cholestasis of pregnancy, alcoholic liver disease, and ABO maternal-fetal blood group incompatibility. Currently, YZHG is widely used in the treatment of viral hepatitis in clinic [9, 10]. Based on the treatment of viral hepatitis with YZHG, this study explored the mechanism of YZHG in inhibiting the inflammation-cancer transformation of hepar by integrated bioinformatics [11–14]. The purpose of the present study is to discuss the disease process of hepatitis B-hepatitis B-related cirrhosis, hepatitis B-related cirrhosis-hepatitis B-related hepatocellular carcinoma, hepatitis C-hepatitis C-related cirrhosis, hepatitis C-related cirrhosis-hepatitis C-related hepatocellular carcinoma 4. Due to the lack of available data in the GEO database, this research included normal group-hepatitis B, hepatitis B-related hepatocellular carcinoma, hepatitis C, and hepatitis C-related cirrhosis-hepatitis C-related hepatocellular carcinoma 4 groups of data (3 groups of GEO data, one set of disease database data). The overall flow chart of this study is shown in Figure 1.

## 2. Methods

### 2.1. Genes Involved in the Inflammation-Cancer Transformation of Hepar

In this study, human genes correlated with advanced hepatitis B, hepatitis C, and associated hepatocellular carcinoma were obtained from the following four resources:

1. The Gene Expression Omnibus database [15] (GEO, http://www.ncbi.nlm.nih.gov/GEO/) is an international public database that can freely store and obtain high-throughput gene expression and other functional genomic datasets. The included datasets meet the following conditions: (1) human liver tissue samples, including human normal liver tissue samples and hepatitis samples infected with HBV; chronic HBV-related HCC adjacent normal tissue and chronic HBV-related HCC liver tissue; and liver cirrhosis group and liver cancer tissue samples. (2) Each dataset contains at least 3 samples. The “limma” package of R 4.1 software was used to identify DEGs when ∣log2FC | ≥1 and adj.*P* < 0.05 were considered to be DEGs [16]
2. The Therapeutic Target Database [17] (TTD, http://db.idrblab.net/ttd/) is a database containing known targets, explored therapeutic proteins, disease targets, nucleic acid targets, pathway information, and corresponding drugs. The keyword “hepatitis C” was used to search for hepatitis C-related targets in TTD
3. Pharmacogenomics Knowledgebase [18] (PharmGKB, https://www.pharmgkb.org) is applied to collect, guide, and disseminate knowledge on clinical operable gene-drug associations and genotype-phenotype relationships. The keyword “hepatitis C” was screened in PharmGKB to obtain known hepatitis C-related targets

### 2.2. Active Ingredients and Putative Targets of YZHG

A literature search in CNKI and PubMed was performed to find the chemical ingredients of YZHG in this investigation. To collect their simplified molecular input line entry specification (SMILES) information, the obtained compounds were entered into the PubChem database [19–23] (https://pubchem.ncbi.nlm.nih.gov). ChemDraw was used to depict the structures of compounds without the standard information from SMILES [24], because the targets of compounds cannot be properly predicted without correct structural information.

All compounds' SMILES information was entered into the SuperPred and SwissTargetPrediction databases search tools. The server for chemical composition, ATC code, and target prediction is the SuperPred database (https://prediction.charite.de). It can anticipate the ATC coding or target of small molecules and acquire compound information, which is useful for drug development [25]. SwissTargetPrediction (https://www.swisstargetprediction.ch/) is a site that uses the reverse screening similarity principle to suggest the most likely protein targets for small compounds [26]. Only human-related targets were chosen for further study once the matching known or anticipated targets were collected from the SuperPred and SwissTargetPrediction databases. Cytoscape 3.7.1 [27] was used to create the YZHG compound-putative target network. Three indices are assigned to each node in the network. Three indices (important parameters) are assessed for each node in the network to evaluate its topological features, including degree, betweenness, and proximity. The number of edges related to the node is represented by “degree.” The stronger the relationship between nodes, the greater the degree. The ratio of the number of the shortest pathways through this node to the total number of paths through all nodes is called “betweenness,” and “closeness” is the reciprocal of the sum of the distances between this node and other nodes. The higher the node's three parameters, the more important it is in the network [28–30].

### 2.3. YZHG-Inflammation-Cancer Transformation of Hepar Network Construction

Protein-protein interaction (PPI) analysis was performed on the hepatitis C data. The 4 groups of disease data (normal group-hepatitis B DEG data, hepatitis B-hepatitis B-related hepatocellular carcinoma DEG data, hepatitis C protein-protein interaction data, hepatitis C-related cirrhosis-hepatitis C-related hepatocellular carcinoma DEG data) and the common targets in the compound-putative target of the YZHG network were determined to be the potential targets in the treatment of inflammation-cancer transformation of the hepar process [31].

### 2.4. Construction of PPI and Key Module Network

In this study, the potential targets of the above 4 groups of YZHG in the treatment of inflammation-cancer transformation of the hepar process were entered into the STRING [32] (https://string-db.org/) database, and the relevant information of PPI was retrieved, respectively, with the species limited to “*Homo sapiens*” and confidence scores higher than 0.7 (low: <0.4; medium: 0.4 to 0.7; and high: >0.7). The “CytoHubba” plug-in sorts the nodes in the network according to the network characteristics and provides 11 topological analysis methods to discover the most important targets and subnetworks of complex networks [33]. In this study, Cytoscape was used to visualize the selected modules and CytoHubba was used to analyze the module network. The maximum clique centrality (MCC) algorithm was selected to determine the top 10 genes with the highest score as key genes.

### 2.5. Enrichment Analysis of Key Genes

To elucidate the role of potential targets in gene function and signal transduction pathways, GO enrichment analysis and KEGG pathway enrichment analysis of targets in the inflammation-cancer transformation of the hepar target network were performed using the g:Profile (https://biit.cs.ut.ee/gprofiler/gost) database [34, 35]. GO enrichment analysis is divided into three aspects: cellular components, molecular functions, and biological processes, revealing possible biological processes associated with key targets. GO and KEGG results were visualized using the “GOplot” package in R software [36].

### 2.6. Molecular Docking

The small-molecule ligands (mol2 format) of YZHG were downloaded from the PubChem database (https://pubchem.ncbi.nlm.nih.gov/). They were processed using AutoDock Tool 1.5.6 software, including the addition of polar hydrogen atoms and Gasteiger charges, and then, the corresponding pdbqt format was saved. The crystal structure of the protein receptor was obtained from the RCSB PDB (http://www.rcsb.org/) protein database. Water molecules and original ligands in protein receptors were removed and saved in pdb format. A script file was created containing the three-dimensional coordinates of the active sites and the number of independent docking calculations; then, polar hydrogen atoms and Gasteiger charge were added to the macromolecular receptor using AutoDock Tool 1.5.6 software, and the corresponding pdbqt format was saved [37, 38]. Finally, molecular docking was performed using AutoDock Vina, with results set to return 20 conformations, and then, PyMOL (http://www.PyMOL.org) was used to view and analyze the results.

## 3. Results

### 3.1. Identification of Inflammation-Cancer Transformation of Hepar Genes

**Figure 2:**
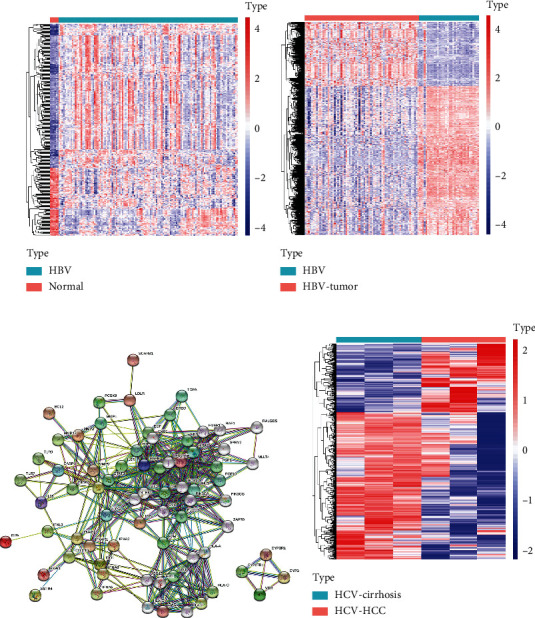
Differentially expressed genes in advanced hepatitis B, hepatitis C, and related hepatocellular carcinoma: (a) heat map of GSE83148; (b) heat map of GSE121248; (c) PPI network of the HCV disease database; (d) heat map of GSE17548.

The GSE83148 dataset included 6 normal human liver tissue samples and 122 HBV-infected hepatitis samples. Supplement Table S1 (Figure 2(a)) shows the results of difference analysis of the GSE83148 dataset, including 263 DEGs composed of 83 downregulated genes and 180 upregulated genes. The GSE121248 dataset contains 37 chronic hepatitis B-induced HCC adjacent normal tissues and 70 chronic hepatitis B-induced HCC liver tissues. Supplement Table S2 (Figure 2(b)) shows the difference analysis results of the GSE121248 dataset, including 798 DEGs composed of 559 downregulated genes and 239 upregulated genes. The GSE17548 dataset comprises 3 hepatitis C-related cirrhosis samples and 3 hepatitis C-related hepatocellular carcinoma samples. Supplement Table S4 (Figure 2(d)) shows the results of difference analysis of the GSE17548 dataset, including 706 DEGs containing 223 upregulated genes and 483 downregulated genes. Around 39 hepatitis C targets were retrieved in TTD and PharmGKB, and protein-protein interactions between 72 targets were analyzed using the STRING database (Supplementary Table S3, Table 1, Figure 2(c)). The target with the highest degree is PIK3CA.

### 3.2. Analysis of Active Ingredients and Putative Target Network of YZHG

**Figure 3:**
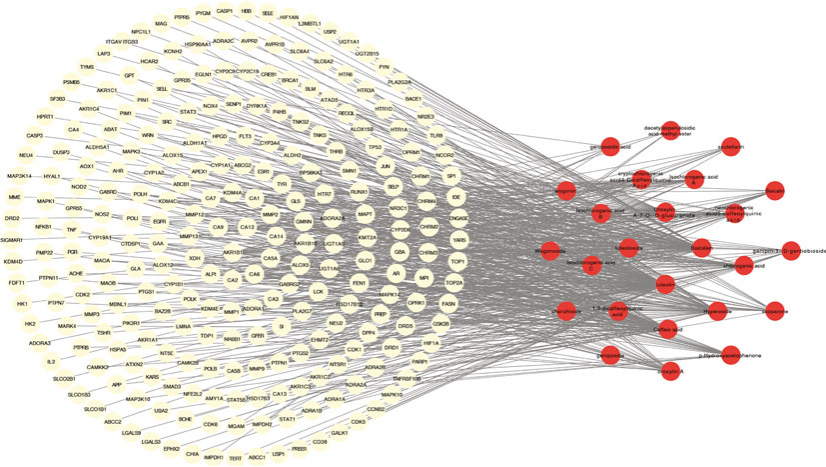
YZHG compound-putative target network. Yellow means putative targets; red indicates the YZHG compound.

A literature search in CNKI and PubMed identified 25 compounds in YZHG (Supplementary Table S5) [11]. The components in YZHG may play a synergistic effect in treating diseases. The network of active ingredients and putative targets of YZHG was constructed by using Cytoscape. As shown in Figure 3, the network consists of 281 nodes (25 active ingredients and 256 putative targets) and 614 edges (Supplementary Tables S6 and S7). In addition, the network analysis showed that the average degree of the compounds is 24.56, indicating that YZHG has the characteristics of a multitarget in the treatment of hepatitis B. There are 8 compounds with a degree value ≥ 24.56 in the network, and the first 3 compounds that play an important role in the network are luteolin (degree = 120), baicalein (degree =86), and caffeic acid (degree = 80).

### 3.3. YZHG-Inflammation-Cancer Transformation of the Hepar Network

**Figure 4:**
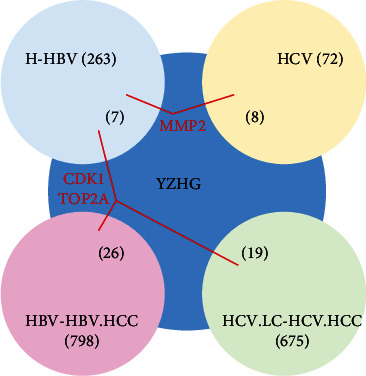
YZHG-disease association network.

Take the intersection of the YZHG target and the above four groups of disease differential genes, and get four groups of disease data. Normal group-hepatitis B (GSE83148) identified 263 DEGs, hepatitis B-hepatitis B-related hepatocellular carcinoma (GSE121248) contained 798 DEGs, 72 hepatitis C protein-protein interaction data and hepatitis C-related cirrhosis-hepatitis C-related hepatocellular carcinoma (GSE17548) contained 706 DEGs. The above DEG data (Supplementary Table S8) were combined with the putative targets of YZHG to obtain a drug-disease association network (Supplementary Table S9, Figure 4). There are 7 common targets in the data of normal group-hepatitis B and the putative targets of YZHG, 26 common targets between the data of hepatitis B-hepatitis B-related hepatocellular carcinoma and the putative targets of YZHG, 8 common targets between the data of protein-protein interaction of hepatitis C and the putative targets of YZHG, and 19 common targets between the data of hepatitis C-related cirrhosis-hepatitis C-related hepatocellular carcinoma and the putative targets of YZHG. It is worth noting that the normal group-hepatitis B and hepatitis C protein-protein interaction data share the common target MMP2 with the 5predicted targets of YZHG. The normal group-hepatitis B, hepatitis B-hepatitis B-related hepatocellular carcinoma, and hepatitis C-related cirrhosis-hepatitis C-related hepatocellular carcinoma data have 2 common targets CDK1 and TOP2A with the predicted targets of YZHG.

### 3.4. PPI Analysis and Module Analysis

**Figure 5:**
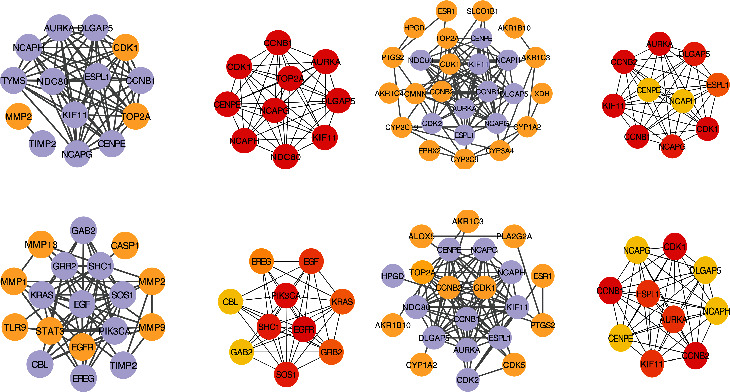
PPI network and module analysis network. (a) (left) Normal group-common target PPI network between hepatitis B data and predicted targets of YZHG; (right) module analysis of the CytoHubba plug-in identified the top 10 genes to construct key module network 1. (b) (left) The hepatitis B-hepatitis B-related hepatocellular carcinoma data and the PPI network of YZHG; (right) module analysis was performed using the CytoHubba plug-in to obtain the top 10 genes and construct key module network 2. (c) (left) Hepatitis C PPI data and PPI network of YZHG; (right) the top 10 genes were obtained to construct key module network 3 after the CytoHubba plug-in module analysis. (d) (left) PPI network of hepatitis C-related cirrhosis-hepatitis C-related hepatocellular carcinoma; (right) module analysis of the CytoHubba plug-in identified the top 10 genes to construct key module network 4. Left: orange circle represents a common target, and purple means related protein information obtained by PPI. Right: the top five genes with the highest scores are represented by yellow to red, and the redder the color, the higher the MCC score.

The PPI analysis was performed for the common targets of the YZHG-disease association network, and the PPI network was constructed by Cytoscape (Figure 5). The normal group-common target PPI network between hepatitis B data and predicted targets of YZHG includes 14 nodes and 66 edges. Module analysis of the CytoHubba plug-in identified the top 10 genes to construct key module network 1, which includes 10 nodes and 45 edges (Figure 5(a)). The hepatitis B-hepatitis B-related hepatocellular carcinoma data and the PPI network of YZHG predicting common targets contain 27 nodes and 100 edges. Module analysis was performed using the CytoHubba plug-in to obtain the top 10 genes and construct key module network 2, which consists of 10 nodes and 45 edges (Figure 5(b)). Hepatitis C PPI data and PPI network of YZHG predicting common targets comprise 18 nodes and 64 edges. The top 10 genes were obtained to construct key module network 3 after the CytoHubba plug-in module analysis, which includes 10 nodes and 39 edges (Figure 5(c)). The PPI network of hepatitis C-related cirrhosis-hepatitis C-related hepatocellular carcinoma and YZHG predicting common targets embodies 22 nodes and 84 edges. Module analysis was performed by applying the CytoHubba plug-in to acquire the top 10 genes and construct key module network 4, which includes 10 nodes and 45 edges (Figure 5(d)).

### 3.5. Enrichment Analysis of Key Genes

**Figure 6:**
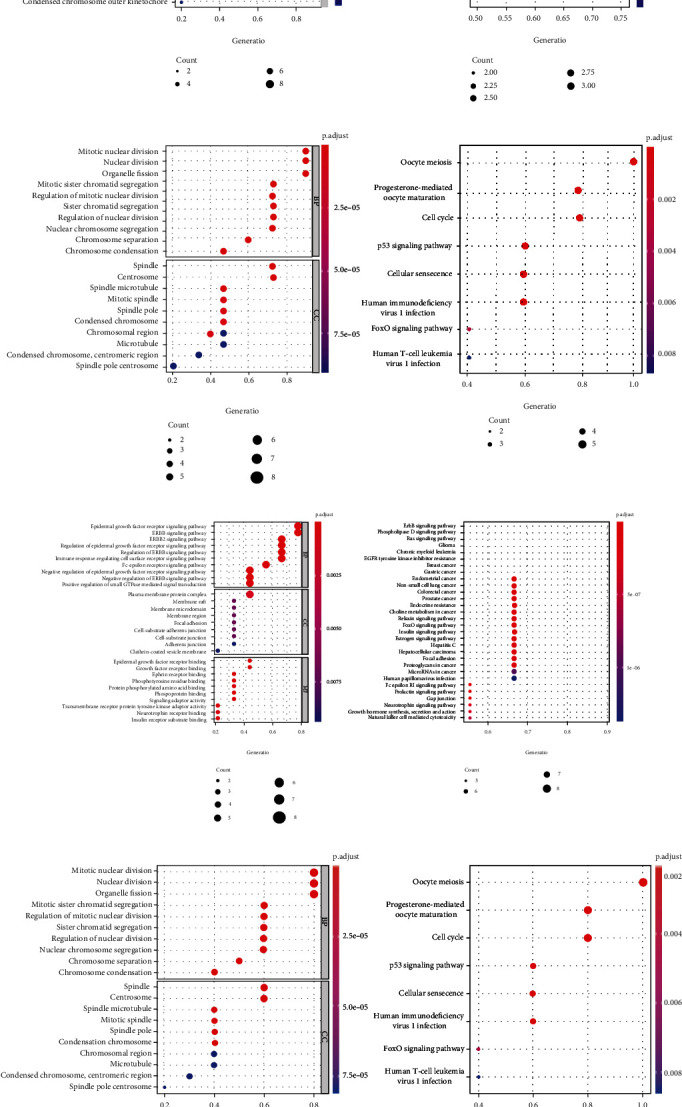
Enrichment analysis of key genes. (a) Enrichment result of key module 1. (b) Enrichment result of key module 2. (c) Enrichment result of key module 3. (d) Enrichment result of key module 4. The left side is the result of GO enrichment, and the right side is the result of KEGG enrichment.

Gene Ontology is used to measure gene function in terms of biological processes (BP), molecular functions (MF), and cellular components (CC). Gene function is comprehensively defined from three different perspectives. Among them, the biological process shows the involvement of genes in different biological processes, the molecular function shows the function of the gene, and the cellular component shows the distribution of the gene in the cell. In this study, GO and KEGG enrichment analysis of the key genes obtained by 4 groups of module analysis was performed to systematically elucidate the multiple mechanisms of YZHG in the treatment of inflammation-cancer transformation of hepar (Figure 6). The results indicated that key module network 1 (Figure 6(a)) was highly correlated with the biological process, and the key targets were enriched in the nuclear division, organelle fission, mitotic nuclear fission, condensed chromosomes, spindles and chromosome regions, progesterone-mediated oocyte maturation pathway, oocyte meiosis pathway, and p53 signaling pathway. Key module network 2 (Figure 6(b)) was closely associated with mitotic nuclear division, nuclear division, organelle fission, spindle, centrosome, spindle microtubules, oocyte meiosis signaling pathway, progesterone-mediated oocyte maturation signaling pathway, and cell cycle signaling pathway. Key module network 3 (Figure 6(c)) was highly associated with the epidermal growth factor receptor signaling pathway, ERBB signaling pathway, ERBB2 signaling pathway, plasma membrane protein complexes, membrane rafts, membrane microdomains, epidermal growth factor receptor binding, growth factor receptor binding, ephrin receptor binding, ErbB signaling pathway, phospholipase D signaling pathway, and Ras signaling pathway. Key module network 4 (Figure 6(d)) was closely linked to mitotic nuclear division, nuclear division, organ fission, spindle, centrosome, spindle microtubules, oocyte meiosis signaling pathway, progesterone-mediated oocyte maturation signaling pathway, and cell cycle pathway.

### 3.6. Molecular Docking Results

**Figure 7:**
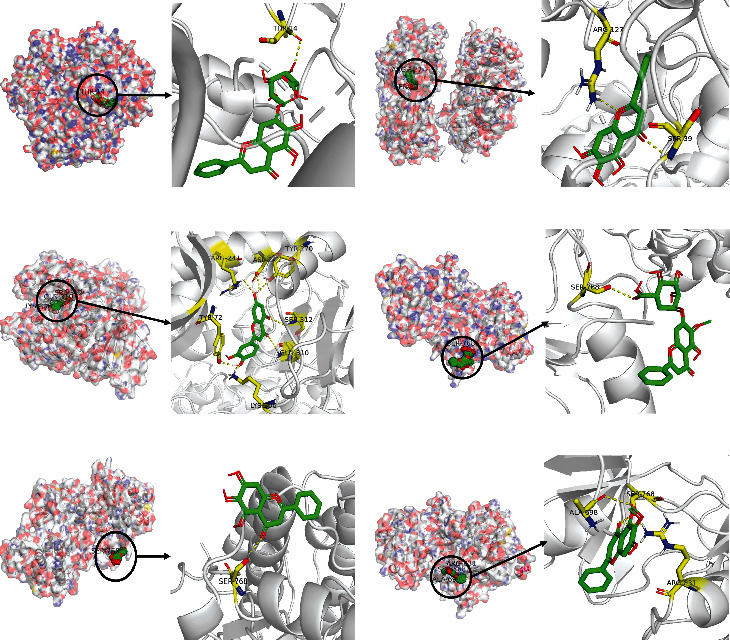
Molecular docking results. (a) CDK1-baicalin; (b) CDK1-baicalein; (c) TOP2A-luteolin; (d) EGFR-oroxylin A-7-O-*β*-D-glucuronide; (e) EGFR-baicalein; (f) EGFR-oroxylin A.

The mechanism of YZHG in the treatment of inflammation-cancer transformation of hepar is reflected in the interaction between compounds and targets. Through the construction of the PPI network and module analysis, the directly related targets in the key modules, namely, CDK1, TOP2A, EGFR, and CCNB2, were selected to investigate the interaction of other corresponding compounds. The three-dimensional crystal structures of 4 targets were derived from the PDB database as their PDB codes. For CCNB2, the corresponding PDB code was not found. AutoDock Vina was applied to dock the above 3 targets and their corresponding small-molecule drug structural ligands. As shown in Supplementary Table S10, 7 groups of molecular docking results were obtained. The highest affinity docking results were luteolin and TOP2A. In addition, the positive drug entecavir was selected as a control group for HBV-positive liver disease, and the baseline of HBV-positive entecavir was established. The positive drug ribavirin was selected as the control group for HCV-positive liver disease, and the baseline of HCV-positive ribavirin was established. The results showed that only the docking result of caffeic acid and EGFR was lower than that of the positive control group, and the molecular docking affinity results of the other groups were more significant than those of the positive control group. The docking combination of the 6 groups with higher docking results than those of the positive control is shown in Figure 7.

## 4. Discussion

YZHG is a commonly used drug in the clinic for the treatment of liver diseases [39–41]. However, its mechanism of action in the adjuvant treatment of acute and chronic liver disease has not been fully elucidated. This study focuses on the effective active ingredients and the mechanism of YZHG in inhibiting the inflammation-cancer transformation of hepar and expends the scope of YZHG. The aim is to reveal the targeting of YZHG in the treatment of hepatitis and to provide a scientific and reasonable basis for the adjuvant medicinal use of inflammation-cancer transformation of hepar. Chronic viral hepatitis affects 500 million people worldwide and leads to cirrhosis, cancer, and liver failure 1. Due to the difficulty of viral clearance in chronic viral hepatitis, liver damage is associated with the body's immune function. Liver protection is usually recommended as the primary treatment. In patients with hepatitis B and hepatitis C, antiviral therapy should be taken actively. For patients with liver cancer due to hepatitis and cirrhosis, we should pay attention to the importance of antiviral therapy, and early antiviral therapy can effectively control the progression of liver cancer. In the present study, the network pharmacology method was used to explore the active components and targets of YZHG and the common targets of YZHG in the process of inflammation-cancer transformation of hepar by module analysis, enrichment analysis, and molecular docking to understand and predict the potential mechanism of YZHG in inhibiting the inflammation-cancer transformation of hepar.

According to the YZHG-disease association network, the hepatitis B and hepatitis C PPI data in the normal group had a common target, MMP2, with the predicted target of YZHG. The data of normal group-hepatitis B, hepatitis B-hepatitis B-related hepatocellular carcinoma, and hepatitis C-related cirrhosis-hepatitis C-related hepatocellular carcinoma all had two common targets CDK1 and TOP2A with YZHG putative targets.

Matrix metalloproteinase-2 (MMP2), a member of the matrix metalloproteinase (MMP) gene family, is a zinc-dependent enzyme that can cleave extracellular matrix components and participate in signal transduction. The protein encoded by the gene is gelatinase A, type IV collagenase, and its catalytic site contains 3 fibronectin type II repeats that bind denatured type IV collagen and type V collagen to elastin. Unlike most members of the MMP family, activation of this protein can occur at the cell membrane. It can inhibit cell migration and adhesion, activate mitochondrial-nuclear stress signaling, induce the activation signal of nuclear transcription factor B, and activate T nuclear factor interferon regulatory factor, promoting vascular remodeling and angiogenesis to form tumor invasion tissues [42]. Yang et al. found that MMP2/MMP9-mediated CD100 shedding is important for the induction of intrahepatic anti-HBV CD8 T-cell response and HBV clearance [43]. The expression of mCD100 on T-cells was higher in patients with chronic hepatitis B, and the serum sCD100 level was lower than that in the healthy control group. Therapeutic sCD100 treatment led to activation of dendritic cells and hepatic sinusoidal endothelial cells, enhanced HBV-specific CD8 T-cell response, and accelerated HBV clearance, while blockade of its receptor CD72 attenuated intrahepatic anti-HBV CD8 T-cell response. Together with MMP9, MMP2 mediates shedding of mCD100 from the surface of T-cells. Serum MMP2 levels in patients with chronic hepatitis B were significantly decreased, which was positively correlated with serum levels of the soluble intercellular adhesion molecule D100. Inhibition of MMP2/MMP9 activity resulted in reduced anti-HBV T-cell response and delayed HBV clearance in mice. In chronic HCV infection, pathological accumulation of the extracellular matrix is the main feature of liver fibrosis. Degradation of connective tissue proteins was shown to reduce the increase in matrix synthesis. Matrix metalloproteinases (MMPs) play a crucial role in extracellular matrix remodeling. Abdel-Latif studied plasma MMP2 in 15 cases of liver fibrosis with HCV RNA detection, 10 cases of liver cirrhosis with HCV, and 15 age-matched and gender-matched control subjects and found that MMP2 could be used as a prognostic marker for liver fibrosis [44]. Therefore, MMP2 plays an important role in HBV- and HCV-related hepatitis in the published studies.

CDK (cyclin-dependent kinases) is a Ser/Thr kinase system that corresponds to cell cycle progression. Activation of CDK1 can phosphorylate the target protein and cause corresponding physiological effects, such as nuclear fiber layer protein phosphorylation leading to nuclear fiber layer disintegration, nuclear membrane disappearance, and H1 phosphorylation leading to chromosome condensation. The end results of these effects make the cell cycle continuous. Hepatitis B virus X protein (HBx) is a multifunctional regulatory protein known to be involved in viral proliferation, transcriptional activation, and cell growth control [45]. Cheng et al. further studied the effect of HBx on cell growth *in vitro* and *in vivo*. HBx can inhibit the growth of hepatocellular carcinoma (HCC) cells and induce G2/M phase arrest in vitro. HBx continuously activates cyclin B1-CDK1 kinase. *In vivo*, HBx inhibits tumor cell growth and induces apoptosis and inhibits the vascular endothelial cell growth [46].

In summary, HBx induces G2/M arrest and apoptosis through continuous activation of cyclin B1-CDK1 kinase and negatively regulates cell growth in vitro and in vivo. HCV core proteins disrupt the G1/S and G2/M phases by regulating the expression and activity of several cell cycle regulators [46]. Viral proteins increase the activity of the cyclin B1-CDK1 complex via p38 MAPK and JNK pathways. CDK1 plays an essential role in HCV-related liver diseases.

TOP2A encodes a DNA topoisomerase that controls and alters the topological state of DNA during transcription. The ribozyme is involved in chromosome condensation, chromatid separation, and the reduction of torsional stress during DNA transcription and replication [47]. It catalyzes the fast breaking and rebinding of the two strands of DNA, making the two strands pass through each other, thus altering the topological structure of DNA. Genes encoding this enzyme act as targets for various anticancer drugs, and multiple mutations in this gene have been linked to the development of drug resistance. Panvichian et al. found that TOP2A overexpression was significantly associated with HCC tumor tissue (*P* < 0.001), serum HBsAg (*P* = 0.004), and Ki-67 (*P* = 0.038). Ignat et al. used quantitative RT-PCR to verify the coexpression of linking genes in a group of normal liver tissues (*n* = 8), chronic liver diseases (*n* = 7), and hepatocellular carcinoma (*n* = 7) induced by hepatitis C virus (*n* = 9). TOP2A was significantly upregulated in dedifferentiated hepatocellular carcinoma and hepatocellular carcinoma with loss of chromosome 13q [48, 49].

Molecular docking results showed that the binding ability of CDK1, TOP2A, and EGFR and their corresponding compounds was greater than that of the positive control group. Epidermal growth factor receptor (EGFR) is the receptor for cell proliferation and signal transduction of epithelial growth factor (EGF). Studies have shown that EGFR is highly or abnormally expressed in many solid tumors. EGFR is associated with tumor cell proliferation, angiogenesis, tumor invasion, metastasis, and inhibition of apoptosis. Wen et al. and Lo et al. found that EGFR has the most significant antitumor effect in treating liver cancer [50, 51].

YZHG is used in clinical application for acute and chronic hepatitis and severe hepatitis caused by the damp-heat toxin and can also be used for comprehensive treatment of other types of severe hepatitis [52–54]. In this study, network pharmacology and bioinformatics methods were used to explore and predict the key targets and potential mechanisms of YZHG in inhibiting the inflammation-cancer transformation of hepar. There is a common target MMP2 for hepatitis B and hepatitis C, which plays an important role in treating hepatitis with YZHG. Also, CDK1 and TOP2A may play an essential role in the inhibition of YZHG in the inflammation-cancer transformation of hepatitis B. KEGG pathway enrichment showed that key genes were mainly enriched in the progesterone-mediated oocyte maturation and oocyte meiosis pathway in modules 1, 2, and 4.

## 5. Conclusion

In conclusion, the present study explored and predicted the key targets and potential mechanisms of YZHG in inhibiting the inflammation-cancer transformation of hepar by network pharmacology and bioinformatics. It is hoped that this study will lay a good foundation for further experimental research and contribute to the application of network pharmacology in exploring the potential mechanism of complex diseases. Since this study is based on data analysis, further *in vitro* and *in vivo* data are needed to validate these findings and optimize the method.

## Figures and Tables

**Table 1:** Four groups of disease data retrieval.

Record	PMID	Platform		Group	Note
GSE83148	28328162 30466390 31282064	GPL570	[HG-U133_Plus_2] Affymetrix Human Genome U133 Plus 2.0 Array	Normal/HBV (6/122)	—
GSE121248	17975138	GPL570	[HG-U133_Plus_2] Affymetrix Human Genome U133 Plus 2.0 Array	HBV/HBV-HCC (37/70)	—
—	—	—		HCV	TTD, PharmGKB
GSE17548	23691139	GPL570	[HG-U133_Plus_2] Affymetrix Human Genome U133 Plus 2.0 Array	HCV-LC/HCV-HCC (3/3)	—

## Data Availability

The data used to support the findings of this study are included within the supplementary information files.

## Abbreviations

HCC: Hepatocellular carcinoma
HBV: Hepatitis B virus
HCV: Hepatitis C virus
DEGs: Differentially expressed genes
MMP2: Matrix metalloproteinase-2
CDK1: Cyclin-dependent kinase 1
TOP2A: DNA topoisomerase 2-alpha
EGFR: Epidermal growth factor receptor
EGF: Epithelial growth factor
CCNB2: G2/mitotic-specific cyclin-B2.

## Supplementary Materials

Supplementary Materials Supplementary materials contain eight tables. Supplementary Table S1: the information of differentially expressed genes in GSE83148. Supplementary Table S2: the information of differentially expressed genes in GSE121248. Supplementary Table S3: the information of targets in the PPI network of hepatitis C. Supplementary Table S4: the information of differentially expressed genes in GSE17548. Supplementary Table S5: the information of 25 compounds in YZHG. Supplementary Table S6: relationship between network points of target nodes of YZHG. Supplementary Table S7: relationship between network points of target edges of YZHG. Supplementary Table S8: the information of 4-group disease data. Supplementary Table S9: the information of the drug-disease association network. Supplementary Table S10: the molecular docking result analysis.
