# Supplementary material for: Exploring the Multicomponent Synergy Mechanism of Yinzhihuang Granule in Inhibiting Inflammation-Cancer Transformation of Hepar Based on Integrated Bioinformatics and Network Pharmacology
Source: Biomed Res Int. 2022 Mar 18;2022:6213865. doi: 10.1155/2022/6213865 (PMC8956385; doi:10.1155/2022/6213865)
Supplement: Supplementary Materials — contain eight tables. Supplementary Table S1: the information of differentially expressed genes in GSE83148. Supplementary Table S2: the information of differentially expressed genes in GSE121248. Supplementary Table S3: the information of targets in the PPI network of hepatitis C. Supplementary Table S4: the information of differentially expressed genes in GSE17548. Supplementary Table S5: the information of 25 compounds in YZHG. Supplementary Table S6: relationship between network points of target nodes of YZHG. Supplementary Table S7: relationship between network points of target edges of YZHG. Supplementary Table S8: the information of 4-group disease data. Supplementary Table S9: the information of the drug-disease association network. Supplementary Table S10: the molecular docking result analysis. [file 6213865.f1.zip › Supplement Table S1.pdf]

id	logFC	AveExpr	t	P.Value	adj.P.Val	B
KNCN	-1.573966	3.18045327	-13.69492	5.48E-27	1.11E-22	50.3332057
RBM42	1.20564342	7.45026154	13.2085755	8.58E-26	8.65E-22	47.6814119
HDAC6	1.23669037	9.40519336	12.7463164	1.19E-24	6.00E-21	45.1437997
AKT1	1.30742706	7.68787215	12.0235957	7.42E-23	2.14E-19	41.1497472
RHOB	2.08585129	10.8405295	11.9300593	1.27E-22	3.20E-19	40.6309873
UBA1	1.52093767	8.66323063	11.7167318	4.31E-22	9.67E-19	39.44669
YARS2	-1.0457077	5.20684917	-11.163304	1.04E-20	1.61E-17	36.3692129
LINC00663	-1.49756	4.89854167	-11.124002	1.30E-20	1.87E-17	36.1505218
GLYATL1	-1.3137912	2.48108258	-10.611967	2.46E-19	3.10E-16	33.3020295
AKIRIN2	1.01724656	8.26372482	10.0077859	7.81E-18	7.50E-15	29.9493901
BANF1	1.18301049	7.84775981	9.61572476	7.29E-17	6.12E-14	27.7843828
PEX19	1.09750317	8.37598727	9.58548898	8.65E-17	6.71E-14	27.6179003
ACTN4	1.03323715	8.72195314	9.38252771	2.73E-16	2.04E-13	26.5024905
TIE1	1.18121629	5.94669775	8.85293564	5.38E-15	3.10E-12	23.6129755
FKBP1A	1.26384661	8.40361711	8.79632271	7.38E-15	4.14E-12	23.306209
JUND	1.30733271	9.05608709	8.78407541	7.90E-15	4.31E-12	23.2399054
AP2M1	1.11559233	8.41486643	8.64332459	1.73E-14	8.73E-12	22.4795231
SUZ12P1	-1.246992	3.70703241	-8.4977955	3.88E-14	1.67E-11	21.6966027
CFL1	1.0367096	11.6884713	8.32237519	1.02E-13	3.62E-11	20.7576655
PLD3	1.39626155	7.1436445	8.28210601	1.28E-13	4.32E-11	20.5429093
ILF3-DT	-1.3355798	5.63600892	-8.2806972	1.29E-13	4.32E-11	20.5354015
PFN1	1.04549689	10.4875561	8.25413181	1.49E-13	4.84E-11	20.3939017
IGFBP4	1.38352397	9.4350764	8.23790456	1.63E-13	5.21E-11	20.3075337
SDHAF3	-1.1427064	6.31620889	-8.094814	3.56E-13	1.06E-10	19.5481815
CST3	1.02223879	10.0839956	8.09142235	3.63E-13	1.06E-10	19.5302327
C1RL-AS1	-1.1289726	4.94841081	-7.9286096	8.81E-13	2.19E-10	18.6714603
LRRC25	1.17176655	5.27901098	7.867297	1.23E-12	2.95E-10	18.3495547
SDC3	1.25393865	7.79347684	7.67822143	3.40E-12	7.01E-10	17.3623216
CIDEB	1.0166196	10.8483053	7.67125747	3.53E-12	7.20E-10	17.3261233
ZNF224	-1.2883079	5.44632904	-7.4459868	1.18E-11	1.98E-09	16.1617887
GPX3	1.42525609	10.9328647	7.15229343	5.53E-11	7.97E-09	14.6644241
ADAMTSL4	1.01416828	5.3586772	7.10294597	7.15E-11	1.02E-08	14.415288
NUDT16	-1.2493985	7.00138899	-7.093018	7.53E-11	1.06E-08	14.3652543
LENG8	1.31768619	6.25071562	6.97255978	1.41E-10	1.87E-08	13.7606165
WBP2	1.2738217	7.27501593	6.95659581	1.53E-10	1.99E-08	13.6808281
TAPBP	1.0533134	8.48667035	6.94931723	1.59E-10	2.05E-08	13.6444765
PLTP	1.42333899	6.48183004	6.93706925	1.69E-10	2.16E-08	13.5833446
CPLANE2	-1.2023068	5.02340233	-6.8816681	2.25E-10	2.75E-08	13.307434
CD74	1.80282723	10.4918569	6.8009649	3.41E-10	4.00E-08	12.9073211
LOC1001299	-1.030996	2.8548693	-6.6960149	5.82E-10	6.38E-08	12.3902913
SGMS1	-1.0688722	6.53493114	-6.5547932	1.19E-09	1.17E-07	11.7006617
SH3BGRL3	1.20792663	8.04265876	6.53563679	1.31E-09	1.27E-07	11.6076689
UBXN2A	-1.1006695	8.05730403	-6.5298829	1.35E-09	1.28E-07	11.5797636
TNFAIP2	1.25250721	7.24832925	6.50417818	1.53E-09	1.43E-07	11.455249

PPP4R4	-1.2110538	3.01003555	-6.4939724	1.61E-09	1.47E-07	11.4058798
PARVG	1.02304159	6.4269862	6.47406405	1.78E-09	1.59E-07	11.3096872
FXYD5	1.10061727	7.07579181	6.4580237	1.93E-09	1.70E-07	11.2322919
LOC1002874	1.21876361	4.55602707	6.29742104	4.29E-09	3.44E-07	10.4627957
IL32	2.42916304	8.13650113	6.25989708	5.17E-09	4.06E-07	10.2844581
NCAM2	-1.6146352	2.6906696	-6.2551512	5.29E-09	4.09E-07	10.2619424
CCDC125	-1.1913635	5.21568851	-6.1855552	7.45E-09	5.46E-07	9.9328061
RTN1	1.28518156	4.944373	6.14796037	8.95E-09	6.31E-07	9.75583174
METTL7B	1.07707083	10.1966914	6.07645367	1.27E-08	8.50E-07	9.42083623
PLEKHA6	-1.3264163	5.84361575	-6.0190847	1.67E-08	1.06E-06	9.15362978
SLC22A3	-1.1678481	6.78272197	-5.9848287	1.97E-08	1.22E-06	8.99474752
DCAF17	1.09084495	5.9617348	5.95818132	2.24E-08	1.38E-06	8.87150542
ZNF160	-1.1893236	7.64160713	-5.9501279	2.33E-08	1.42E-06	8.83431986
GRTP1	-1.1609974	8.17646116	-5.916434	2.74E-08	1.63E-06	8.67905058
GEN1	-1.1399849	6.11514585	-5.8950954	3.04E-08	1.78E-06	8.58097617
PTGR2	-1.1719819	3.58357624	-5.854954	3.68E-08	2.07E-06	8.39703038
ZNF721	-1.1559678	8.45783442	-5.8290148	4.16E-08	2.27E-06	8.27854935
KANSL1L	-1.1221637	5.58455733	-5.7467645	6.14E-08	3.10E-06	7.90488005
PARVB	1.28848661	5.38273844	5.70788542	7.37E-08	3.57E-06	7.72933318
MINDY1	-1.0672295	7.79340475	-5.6826372	8.29E-08	3.90E-06	7.61570969
THAP5	-1.0303289	6.40075418	-5.6772325	8.51E-08	3.99E-06	7.59142623
MMD	1.06849158	7.79175993	5.62464791	1.09E-07	4.92E-06	7.35588062
BTG2	1.22564494	6.43081229	5.60182266	1.21E-07	5.41E-06	7.25404761
C8orf44	-1.0940891	4.16606545	-5.567664	1.42E-07	6.12E-06	7.1021191
HCLS1	1.08092182	8.55631327	5.51638248	1.80E-07	7.51E-06	6.87509553
ARRDC3	-1.3347786	7.79103613	-5.5091602	1.86E-07	7.74E-06	6.84322584
SULT1E1	-1.5270109	7.44486724	-5.4625429	2.30E-07	9.19E-06	6.63813629
NETO2	1.02619961	3.56860931	5.46040621	2.32E-07	9.24E-06	6.62876195
TXNIP	1.04942427	9.78049123	5.44149078	2.53E-07	9.90E-06	6.5458727
HCK	1.11845536	6.60731645	5.43248257	2.64E-07	1.03E-05	6.50646069
PDIA3	-1.0901123	9.90394347	-5.4128388	2.89E-07	1.11E-05	6.4206581
PGF	-1.0040054	8.25902995	-5.3551143	3.75E-07	1.39E-05	6.16964986
ZNF814	-1.2808451	3.87961699	-5.3386374	4.04E-07	1.49E-05	6.09831357
CTSLP8	-1.1985263	4.53628033	-5.3091185	4.62E-07	1.64E-05	5.9708608
RCSD1	1.00317076	6.50812906	5.23106718	6.56E-07	2.22E-05	5.63604001
LCP2	1.10033362	7.09537617	5.22765002	6.66E-07	2.24E-05	5.62145415
P2RY13	1.18036987	6.8614367	5.18943764	7.89E-07	2.59E-05	5.45876829
FCER1G	1.21832228	9.05952288	5.15866952	9.05E-07	2.91E-05	5.32834013
TGFB1	1.22495026	5.2746308	5.151779	9.33E-07	2.99E-05	5.29920017
GPR37	-1.2449291	2.94116307	-5.1450578	9.61E-07	3.06E-05	5.27080072
PRG4	-1.2459595	6.90069822	-5.0830709	1.26E-06	3.81E-05	5.0100356
HAUS2	-1.0916882	8.10325875	-5.0367937	1.55E-06	4.49E-05	4.81672345
HPR	-1.4667953	9.9375896	-5.0356098	1.55E-06	4.50E-05	4.8117932
FGF14-AS2	-1.000289	5.32288722	-4.9650957	2.11E-06	5.84E-05	4.51955919
MMP2	1.47099076	6.29339287	4.95704675	2.19E-06	6.00E-05	4.48637822

ABO	1.11959064	4.63501107	4.93935396	2.36E-06	6.39E-05	4.41356943
SLITRK3	-1.1504303	4.06612384	-4.9323524	2.43E-06	6.54E-05	4.38480545
ITPR2	-1.0139953	8.63025472	-4.9233791	2.53E-06	6.76E-05	4.34798204
SASH3	1.1282654	6.46691285	4.92322286	2.53E-06	6.76E-05	4.34734116
SLCO4C1	-1.3895346	5.61598218	-4.9220099	2.54E-06	6.79E-05	4.3423672
ZBTB10	-1.0289696	6.34019276	-4.9070042	2.71E-06	7.14E-05	4.28090179
BARD1	1.15803768	4.62984457	4.89365357	2.87E-06	7.48E-05	4.22632373
MAB21L2	1.5577654	5.037935	4.86193282	3.29E-06	8.39E-05	4.09705531
PTPRE	1.05000904	6.01446031	4.86098481	3.31E-06	8.41E-05	4.09320085
SLC25A47	1.18667949	9.32604748	4.84120661	3.60E-06	9.04E-05	4.01290367
PDE4B	1.18950571	5.78538129	4.84085357	3.60E-06	9.04E-05	4.01147244
TRHDE	-1.287271	3.00512134	-4.8297572	3.78E-06	9.35E-05	3.96652351
S100A11	1.27610235	6.99803304	4.74422534	5.43E-06	0.00012409	3.62245573
TPX2	1.22619194	5.04377979	4.73551905	5.63E-06	0.00012728	3.58767346
DEPDC1	1.15209969	3.59234192	4.70321882	6.44E-06	0.00014177	3.45902338
PVRIG	1.05876598	5.04949966	4.68387111	6.99E-06	0.00015108	3.38225928
PLP2	1.08955744	6.47912159	4.65369723	7.92E-06	0.00016615	3.26298767
LOC1027244	-1.3579136	5.54141234	-4.623128	8.99E-06	0.00018403	3.14271122
ADA2	1.28973288	8.7753967	4.61759664	9.20E-06	0.00018715	3.12100808
SLC25A43	-1.0175321	6.18782611	-4.5542785	1.19E-05	0.00023147	2.87389462
OSBPL3	1.26679864	4.56498995	4.5214327	1.37E-05	0.00025625	2.74667382
LAIR2	1.02825296	3.9439162	4.50664416	1.45E-05	0.00026882	2.68961106
MAML2	1.01663881	5.70219481	4.46110446	1.75E-05	0.00031084	2.51474588
KLF4	1.00613026	6.8728068	4.45574604	1.78E-05	0.0003157	2.49425547
CD44	1.19253723	8.28731484	4.45344218	1.80E-05	0.00031805	2.48545113
SUSD3	-1.0254837	6.40110364	-4.4481218	1.84E-05	0.00032246	2.46513149
MAP2K6	-1.1152511	6.1639278	-4.4420139	1.89E-05	0.00032823	2.44182639
CCND2	1.37879627	6.06603539	4.44147495	1.89E-05	0.00032837	2.43977116
NR1D2	-1.5723706	6.41172844	-4.3118089	3.17E-05	0.00050392	1.95063926
CAPN2	1.23989441	8.78190419	4.29798282	3.35E-05	0.00052765	1.89911887
HLA-DOA	1.18195744	6.24854259	4.27304596	3.70E-05	0.00057229	1.80650858
AFMID	-1.1622826	5.73860065	-4.2635915	3.84E-05	0.00059033	1.77150218
ESF1	-1.018519	6.39140705	-4.2596565	3.90E-05	0.00059906	1.75694942
TYMS	2.05298441	7.52462164	4.25285486	4.00E-05	0.00061292	1.73181901
FCRL3	1.3904265	5.28448441	4.24640769	4.10E-05	0.0006248	1.70802601
KCNJ3	-1.1959608	4.53373615	-4.2339123	4.31E-05	0.00064972	1.66198963
CNKSR2	-1.3442201	5.10753074	-4.2243544	4.47E-05	0.00066999	1.62684504
ZFPM2	1.26219708	5.48959928	4.18433223	5.23E-05	0.00075761	1.48033366
ITK	1.41289268	5.32769905	4.18003327	5.31E-05	0.00076743	1.46465907
NOL4	-1.0492181	5.5280601	-4.1455253	6.07E-05	0.00084834	1.33928226
BCHE	-1.1540891	10.6261606	-4.1407428	6.19E-05	0.00085935	1.32196862
OAS2	1.23007318	6.75663102	4.13050948	6.43E-05	0.00088592	1.28497299
TM6SF1	1.18808545	4.884924	4.13026232	6.44E-05	0.00088616	1.28408029
CCNA2	1.24675857	4.80421966	4.08298722	7.72E-05	0.0010283	1.11408893
CXCL9	2.64804963	8.5945649	4.0725134	8.03E-05	0.00106454	1.07663062

CD2	1.38361494	6.02445103	4.0516031	8.70E-05	0.00113176	1.00206926
JAZF1	1.17064695	6.38441084	4.04470299	8.93E-05	0.0011521	0.97753
SPDYE2	-1.080956	5.52876705	-4.03138	9.39E-05	0.00120093	0.93024022
IGLC1	2.11273133	10.5087595	4.00748097	0.00010273	0.0012829	0.84571372
MTHFD2	1.4820668	5.60981608	4.00221714	0.00010479	0.00130094	0.82714891
EGR2	1.22872409	3.86067153	3.99932647	0.00010594	0.00131227	0.81696199
CDKN1A	1.0960707	8.48139385	3.99025147	0.00010961	0.00135162	0.7850182
MOGAT1	-1.151219	6.41888679	-3.9870894	0.00011092	0.00136442	0.77390099
CCNE2	1.2389573	4.15331926	3.93380569	0.00013534	0.00159764	0.58760191
SSPN	1.42542111	4.59815756	3.90916624	0.00014829	0.00172133	0.50211734
SAMD9L	1.08832426	6.56351741	3.88422741	0.0001626	0.00185749	0.41602422
CXCL10	2.52415259	8.56141254	3.87065977	0.00017093	0.00193507	0.36936886
FUS	-1.0096724	7.32678922	-3.8656477	0.00017411	0.00196004	0.35216634
STK39	1.02536245	4.62403998	3.86049383	0.00017743	0.00198678	0.33449549
ENPP2	1.32485265	8.56996114	3.85530462	0.00018084	0.00201472	0.31672231
ZNF738	1.05474917	4.68482989	3.83383113	0.00019562	0.002146	0.24337641
TTPA	-1.0850651	6.21860366	-3.831482	0.00019731	0.00216096	0.23537231
MEIOC	-1.0645453	4.7647784	-3.8225309	0.00020386	0.0022087	0.20490943
DTL	1.65896788	4.7442733	3.8220273	0.00020423	0.00221014	0.20319707
PHLDA1	1.05921162	6.46590462	3.81158907	0.00021214	0.00227404	0.16774814
HLA-DMA	1.18736357	8.3879634	3.78874261	0.00023048	0.0024205	0.09042947
CD8A	1.50939547	6.92193305	3.78570003	0.00023304	0.00243633	0.08016052
LOC1019269	-1.4567881	2.60623861	-3.7760705	0.0002413	0.00250407	0.04770344
JAKMIP2	-1.0395457	5.87447871	-3.7723378	0.00024457	0.00252735	0.0351397
TOX	1.41560172	3.73424193	3.76931794	0.00024725	0.00254494	0.02498281
ADAMTS17	-1.0459652	6.04610838	-3.7508834	0.00026423	0.00268276	-0.0368791
BCL2A1	1.24926966	4.57278374	3.74253066	0.00027229	0.00274655	-0.0648287
RGS1	1.27133445	5.43544805	3.73698242	0.00027776	0.00278509	-0.0833665
LCK	1.15136912	6.25153639	3.73130314	0.00028348	0.00282833	-0.1023193
IGKC	1.59219487	8.89604551	3.72759376	0.00028727	0.00285627	-0.1146856
CD69	1.28763931	3.82294714	3.72540178	0.00028953	0.0028717	-0.1219886
IGLL3P	1.39618227	7.11931799	3.72509281	0.00028985	0.00287347	-0.1230177
TRIM22	1.13315578	9.18790482	3.70917093	0.00030683	0.00300482	-0.1759571
NPAS2	1.15498678	6.07771742	3.68254233	0.00033734	0.00322963	-0.2640873
CPEB3	-1.0128367	7.16182431	-3.6787602	0.0003419	0.00326581	-0.276563
PTPN22	1.0839409	4.70765804	3.65465434	0.00037235	0.00348255	-0.3558353
GPR18	1.15721705	5.20303983	3.65027827	0.00037815	0.00351235	-0.3701807
PNMA2	1.13144498	4.8127611	3.63918924	0.00039323	0.00361847	-0.4064699
LONRF2	1.29960705	4.3500313	3.6337191	0.00040087	0.00367098	-0.4243381
P4HA1	1.12266441	7.70444481	3.61700394	0.00042511	0.00385965	-0.4788026
LINC01554	-2.4238402	7.38795297	-3.6105774	0.00043479	0.00393516	-0.4996884
GNL3L	-1.0355818	5.76749969	-3.5999758	0.00045122	0.00405474	-0.5340765
CD27	1.03270156	6.01175492	3.58432141	0.00047655	0.00423336	-0.5847037
GSN	1.54917165	8.82526104	3.5832376	0.00047835	0.00424471	-0.5882021
GPNMB	1.48785824	9.34960762	3.5774574	0.00048808	0.00431107	-0.6068454

NEK2	1.01611369	3.14340238	3.57137571	0.00049851	0.0043802	-0.6264345
PTTG1	1.0186777	6.85263395	3.53167028	0.00057196	0.00487274	-0.7536541
APCDD1	1.4492516	4.91987137	3.5305633	0.00057414	0.00488724	-0.7571843
RRM2	1.93653197	6.08732869	3.5264353	0.00058237	0.0049385	-0.7703403
SERPINE2	1.08694274	7.50631119	3.52547082	0.00058431	0.00494869	-0.7734123
SLC7A1	1.13499085	5.63785798	3.49775113	0.00064266	0.00532796	-0.8614075
GPR88	-2.075069	5.28408215	-3.4954389	0.00064776	0.00536233	-0.8687217
ABCB11	-1.3235051	5.7906281	-3.4902116	0.00065945	0.00544345	-0.8852425
TRAC	1.07029868	6.55306459	3.48988079	0.0006602	0.00544738	-0.8862872
TRBC1	1.16695202	8.05240175	3.46482427	0.00071909	0.00579581	-0.9651873
MYOM1	1.22013589	5.74092937	3.45943847	0.00073238	0.00588176	-0.9820851
COL4A4	1.1944428	5.04052088	3.45809366	0.00073573	0.00589812	-0.986301
CNTN3	-1.4320758	5.87259506	-3.4403976	0.00078123	0.00616365	-1.0416508
TSPAN13	-1.3937103	7.79602185	-3.4345104	0.00079694	0.00625094	-1.0600123
NUSAP1	1.33482772	6.23769795	3.40428295	0.00088239	0.00675315	-1.1538773
CSF2RB	1.01985607	6.18869753	3.39253743	0.00091785	0.00696643	-1.1901639
COL6A2	1.10517698	6.36378854	3.3813299	0.00095294	0.0071692	-1.2246909
SOX4	1.54906615	7.09919258	3.37482459	0.00097387	0.00728741	-1.244688
CACNA2D1	1.02908773	4.67095398	3.36529935	0.0010053	0.00745625	-1.2739102
PLXNC1	1.37410165	5.14063887	3.35264227	0.00104854	0.00768931	-1.3126334
SPATA18	1.1464598	4.37178784	3.34953415	0.00105942	0.00774963	-1.3221237
SLFN12	1.0047092	4.18916342	3.34952456	0.00105946	0.00774963	-1.322153
HINT3	-1.0057555	7.45239742	-3.3085385	0.00121319	0.00853675	-1.4466076
TRAF5	1.02763624	4.95354494	3.2953401	0.00126697	0.00884426	-1.4864102
KCNK5	-1.0139084	7.17768372	-3.274408	0.00135686	0.00930115	-1.54926
EFEMP1	1.94813747	6.4093961	3.26597524	0.00139472	0.00948977	-1.5744843
LEPR	1.1075054	5.09274091	3.21993456	0.00161943	0.01068709	-1.71123
LOC1001909	-1.1695574	6.79323201	-3.2127709	0.00165729	0.01086228	-1.7323586
MROH2A	-1.7961498	6.35611332	-3.2003513	0.00172487	0.01119252	-1.7688945
LINC01410	1.07070873	4.64463196	3.19979131	0.00172798	0.01120547	-1.770539
CCL4	1.03083242	7.37212208	3.18668207	0.00180221	0.0116011	-1.808967
TMEM154	1.45819994	5.78750477	3.18394206	0.0018181	0.01169505	-1.816982
CDK1	1.31659469	5.14694238	3.16904825	0.00190675	0.01207371	-1.860446
GALK1	-1.1088102	7.20530937	-3.1619227	0.00195057	0.01225121	-1.8811786
GZMA	1.11011274	6.44399742	3.16021796	0.00196119	0.01227981	-1.8861328
ASB9	-1.1186221	6.10050769	-3.1520829	0.00201262	0.01248929	-1.9097432
GOLM1	1.36621827	6.45884899	3.12884463	0.00216651	0.01313727	-1.9769
RGS4	1.34654339	5.08109043	3.12169986	0.00221597	0.01337672	-1.9974623
TFEC	1.15191211	4.81523953	3.11327079	0.00227567	0.01362701	-2.0216688
RASGRP1	1.20324316	5.00222008	3.11227684	0.00228281	0.01364949	-2.0245195
STK17B	1.00230691	6.54991477	3.09929618	0.00237794	0.01409714	-2.061677
HPSE	1.02567736	5.76087306	3.09330419	0.00242308	0.01432686	-2.0787842
COL4A3	1.0026633	3.8050978	3.09112215	0.00243972	0.01439867	-2.0850068
PAX8-AS1	-1.081701	5.1502303	-3.0681148	0.00262166	0.01521996	-2.1503881
LOC1005073	-1.059374	10.0405082	-3.0573948	0.00271065	0.01556194	-2.1807078

CCR2	1.03740795	5.73455557	3.038288	0.00287622	0.01627168	-2.2345209
LAMP3	1.17783932	4.68926628	3.02282238	0.00301702	0.01688359	-2.277865
CCL21	1.4741851	6.73789584	3.01805578	0.00306168	0.01708612	-2.2911853
CCL8	1.28978903	5.34298451	3.00306042	0.00320619	0.01769054	-2.332971
MAD2L1	1.2180505	4.26064471	3.00168553	0.00321975	0.01774905	-2.3367932
CCL5	1.04829979	7.83039247	2.99144841	0.00332238	0.01817894	-2.3652046
GZMK	1.0251653	6.8561331	2.97254158	0.0035199	0.01900828	-2.4174554
CXCL8	1.73065207	4.85342113	2.96773735	0.00357179	0.01921013	-2.4306864
ZWINT	1.34169412	6.0604706	2.9574128	0.00368567	0.01970185	-2.4590575
KBTBD11	-1.0152753	6.10788226	-2.9558853	0.0037028	0.01976203	-2.4632476
CHST4	1.22939023	5.83638087	2.91120039	0.00423751	0.02187503	-2.5849872
POU2AF1	1.34583407	4.59207906	2.91059836	0.00424518	0.0219073	-2.5866163
SOX9	1.41946439	6.31959158	2.90219132	0.00435355	0.02231983	-2.609335
ZMAT3	1.03018553	8.03525829	2.89421745	0.00445868	0.02273745	-2.6308299
PCLAF	1.10172024	6.52608613	2.86875832	0.0048101	0.02409706	-2.6991117
DKK3	1.20876487	7.07234525	2.86571934	0.0048537	0.02426727	-2.7072269
PTGDS	1.24458676	8.19278938	2.86008822	0.00493544	0.02454217	-2.7222441
FABP5	1.44352498	6.58925938	2.84814846	0.00511295	0.02521356	-2.7539992
ADAMDEC1	1.23495161	4.26775266	2.83661171	0.00529	0.02586534	-2.7845714
TOP2A	1.34422972	4.05377739	2.77163698	0.00639597	0.02985474	-2.9547052
EHF	1.34542265	6.11987197	2.76669334	0.00648818	0.03016192	-2.967507
CCL19	1.7223512	7.11327999	2.7596778	0.00662112	0.03058753	-2.9856393
HKDC1	1.91162491	5.75235182	2.75961133	0.00662239	0.03058753	-2.9858109
KCNN2	-1.6829795	7.63043773	-2.7468218	0.00687118	0.03139732	-3.0187607
NIBAN1	1.05608389	5.39659801	2.74555774	0.00689623	0.03148324	-3.02201
PLEK2	1.16532431	7.1785457	2.73584862	0.00709137	0.03213419	-3.0469228
SLAMF7	1.19433299	5.77143227	2.73430055	0.00712295	0.03224829	-3.0508877
RCAN2	1.1262703	5.29858341	2.7184335	0.00745402	0.0333431	-3.0914116
LPA	-1.1426644	7.94005955	-2.7106859	0.00762069	0.03393509	-3.1111224
COL5A1	1.02263375	6.83059946	2.70242744	0.00780207	0.03451506	-3.1320777
CLDN10	1.3174063	6.03874776	2.68531989	0.0081903	0.03577982	-3.1753054
ND6	-1.0523206	6.54640562	-2.6474313	0.00911307	0.03879177	-3.2701691
CUX2	-1.1630793	7.61112196	-2.6151984	0.00997091	0.04139806	-3.3499212
RGCC	1.03270647	4.32017323	2.613277	0.01002427	0.04156828	-3.3546475
MGP	1.30525155	6.85607838	2.61154148	0.01007269	0.041724	-3.3589139
PDGFD	1.0102223	6.07989397	2.59820303	0.01045193	0.04290075	-3.3916185
SMOC2	1.16331947	4.16052644	2.58523712	0.01083283	0.04420225	-3.4232652
UHRF1	1.02136554	4.3744011	2.55262655	0.0118466	0.04735355	-3.5022289
CXCL11	1.86990425	4.50551124	2.54210941	0.01219125	0.04838926	-3.5275023

change
down
up
up
up
up
up
down
down
down
up
up
up
up
up
up
up
up
down
up
up
down
up
up
down
up
down
up
up
up
down
up
up
down
up
up
up
up
down
up
down
down
up
down
up

down
up
up
up
up
down
down
up
up
down
down
up
down
down
down
down
down
down
up
down
down
up
up
down
up
down
down
up
up
up
down
down
down
down
up
up
up
up
up
down
down
down
down
down
up

up
down
down
up
down
down
up
up
up
up
up
down
up
up
up
up
up
down
up
down
up
up
up
up
up
down
down
up
down
up
up
down
down
up
up
down
down
up
up
down
down
up
up
up
up

up
up
down
up
up
up
up
down
up
up
up
up
down
up
up
up
down
down
up
up
up
up
down
down
up
down
up
up
up
up
up
up
up
up
down
up
up
up
up
up
down
down
up
up
up

up
up
up
up
up
up
down
down
up
up
up
up
down
down
up
up
up
up
up
up
up
up
down
up
down
up
up
down
down
up
up
up
up
down
up
down
up
up
up
up
up
up
up
down
down

up
up
up
up
up
up
up
up
up
down
up
up
up
up
up
up
up
up
up
up
up
up
up
down
up
up
up
up
down
up
up
down
down
up
up
up
up
up
up
