# Supplementary material for: Exploring the Multicomponent Synergy Mechanism of Yinzhihuang Granule in Inhibiting Inflammation-Cancer Transformation of Hepar Based on Integrated Bioinformatics and Network Pharmacology
Source: Biomed Res Int. 2022 Mar 18;2022:6213865. doi: 10.1155/2022/6213865 (PMC8956385; doi:10.1155/2022/6213865)
Supplement: Supplementary Materials — contain eight tables. Supplementary Table S1: the information of differentially expressed genes in GSE83148. Supplementary Table S2: the information of differentially expressed genes in GSE121248. Supplementary Table S3: the information of targets in the PPI network of hepatitis C. Supplementary Table S4: the information of differentially expressed genes in GSE17548. Supplementary Table S5: the information of 25 compounds in YZHG. Supplementary Table S6: relationship between network points of target nodes of YZHG. Supplementary Table S7: relationship between network points of target edges of YZHG. Supplementary Table S8: the information of 4-group disease data. Supplementary Table S9: the information of the drug-disease association network. Supplementary Table S10: the molecular docking result analysis. [file 6213865.f1.zip › Supplement Table S10 (1).pdf]

Target	Compound	PDB ID	Docking Score	Entecavir baseline(HBV+)	Ribeirin baseline(HCV+)
CDK1	Baicalin	4yc6	-8	-6.3	-6.1
CDK1	Baicalein	4yc6	-7	-6.3	-6.1
TOP2A	luteolin	1zxm	-9.5	-8.8	-8.2
EGFR	Oroxylin A-[illegible]	5d41	-4.5	-4.2	-4.1
EGFR	Baicalein	5d41	-4.6	-4.2	-4.1
EGFR	oroxylin A	5d41	-4.7	-4.2	-4.1
EGFR	Caffeic acid	5d41	-3.9	-4.2	-4.1
CCNB2	luteolin	无蛋白结构	None	None	None
