# Supplementary material for: Exploring the Multicomponent Synergy Mechanism of Yinzhihuang Granule in Inhibiting Inflammation-Cancer Transformation of Hepar Based on Integrated Bioinformatics and Network Pharmacology
Source: Biomed Res Int. 2022 Mar 18;2022:6213865. doi: 10.1155/2022/6213865 (PMC8956385; doi:10.1155/2022/6213865)
Supplement: Supplementary Materials — contain eight tables. Supplementary Table S1: the information of differentially expressed genes in GSE83148. Supplementary Table S2: the information of differentially expressed genes in GSE121248. Supplementary Table S3: the information of targets in the PPI network of hepatitis C. Supplementary Table S4: the information of differentially expressed genes in GSE17548. Supplementary Table S5: the information of 25 compounds in YZHG. Supplementary Table S6: relationship between network points of target nodes of YZHG. Supplementary Table S7: relationship between network points of target edges of YZHG. Supplementary Table S8: the information of 4-group disease data. Supplementary Table S9: the information of the drug-disease association network. Supplementary Table S10: the molecular docking result analysis. [file 6213865.f1.zip › Supplement Table S2.pdf]

id	logFC	AveExpr	t	P.Value	adj.P.Val	B	change
CXCL14	-4.01281	7.240085	-17.383	5.48E-33	1.11E-28	64.24857	down
ANGPTL6	-1.70028	7.407515	-16.6695	1.48E-31	7.99E-28	61.04037	down
VIPR1	-1.40339	7.59019	-16.6615	1.53E-31	7.99E-28	61.00419	down
IGFALS	-2.08697	7.083233	-16.6547	1.58E-31	7.99E-28	60.97307	down
CLEC1B	-3.16985	7.214871	-16.1494	1.70E-30	6.85E-27	58.65909	down
HHIP	-2.84283	6.498016	-15.5602	2.81E-29	9.45E-26	55.91902	down
CDHR2	-2.48948	8.28253	-15.3629	7.26E-29	2.09E-25	54.9912	down
ECM1	-1.71829	7.876044	-15.1049	2.53E-28	6.38E-25	53.7708	down
KCNN2	-4.03311	6.626172	-14.9737	4.79E-28	1.07E-24	53.14698	down
ADAMTS13	-1.67102	7.578449	-14.7331	1.55E-27	3.13E-24	51.99774	down
OIT3	-3.27895	6.973972	-14.5473	3.86E-27	7.08E-24	51.105	down
FCN2	-3.67975	6.706401	-14.4062	7.73E-27	1.30E-23	50.4246	down
CLEC4M	-2.90553	6.684988	-14.3504	1.02E-26	1.58E-23	50.15495	down
CLEC4G	-3.20472	7.009734	-14.2883	1.38E-26	1.99E-23	49.85438	down
PLVAP	1.325803	7.541448	14.1624	2.58E-26	3.47E-23	49.24324	up
ZGPAT	-2.22408	9.653998	-14.1081	3.38E-26	4.27E-23	48.97909	down
CAP2	2.4624	8.268728	14.08	3.89E-26	4.62E-23	48.84239	up
STAB2	-1.71759	6.216714	-13.7147	2.41E-25	2.70E-22	47.05612	down
CRHBP	-3.03987	6.913945	-13.631	3.66E-25	3.89E-22	46.64508	down
LINC01093	-4.19988	7.645695	-13.5585	5.27E-25	5.32E-22	46.2877	down
CYP26A1	-2.75074	7.018521	-13.5408	5.76E-25	5.53E-22	46.20087	down
ACADS	-1.34049	8.473085	-13.5145	6.58E-25	6.03E-22	46.07087	down
ANKRD55	-1.14809	4.652445	-13.4779	7.91E-25	6.93E-22	45.89054	down
KDM8	-1.92801	7.736944	-13.3426	1.56E-24	1.31E-21	45.22194	down
PAMR1	-1.1369	6.896914	-13.2	3.21E-24	2.59E-21	44.51499	down
COLEC10	-1.26487	5.956204	-13.1018	5.29E-24	4.01E-21	44.02705	down
TOP2A	3.266608	7.048017	13.09864	5.37E-24	4.01E-21	44.0114	up
PTH1R	-1.31329	6.994273	-13.0651	6.37E-24	4.59E-21	43.84462	down
NXF3	-1.08279	5.370026	-12.9836	9.63E-24	6.70E-21	43.43879	down
FCN3	-3.34854	8.635042	-12.8525	1.88E-23	1.26E-20	42.78446	down
RIPOR3	-2.52175	7.106669	-12.8158	2.26E-23	1.47E-20	42.60061	down
DBH	-1.01379	6.343319	-12.7482	3.19E-23	2.01E-20	42.2625	down
LCAT	-2.54752	8.883132	-12.691	4.27E-23	2.61E-20	41.9761	down
CNDP1	-3.76002	7.930552	-12.6495	5.28E-23	3.14E-20	41.76775	down
RACGAP1	2.011327	7.475049	12.61417	6.33E-23	3.65E-20	41.59068	up
ASPM	2.863928	6.635216	12.37957	2.11E-22	1.18E-19	40.41095	up
CCBE1	-1.79177	5.84188	-12.3352	2.64E-22	1.44E-19	40.18722	down
RSPO3	-1.93617	5.985339	-12.3223	2.82E-22	1.50E-19	40.12242	down
BMPER	-1.23169	3.950931	-12.3164	2.91E-22	1.51E-19	40.09238	down
ADGRG7	-2.26206	6.315682	-12.1689	6.21E-22	3.13E-19	39.34793	down
IDO2	-3.34742	6.973739	-12.1189	8.04E-22	3.96E-19	39.09478	down
COL15A1	3.001358	6.841314	12.09256	9.21E-22	4.42E-19	38.96159	up
PLAC8	-2.11345	6.88806	-11.9842	1.61E-21	7.55E-19	38.41297	down
CFP	-1.66904	7.276597	-11.9676	1.75E-21	8.05E-19	38.32848	down
CSRNP1	-1.51174	8.182374	-11.8882	2.64E-21	1.19E-18	37.92597	down
PSMD4	1.006978	10.79133	11.77182	4.83E-21	2.12E-18	37.3347	up
HMMR	2.227064	6.979643	11.7553	5.26E-21	2.26E-18	37.2507	up

PITPNM3	-1.36889	4.754883	-11.6945	7.20E-21	2.97E-18	36.9414	down
TBXA2R	-1.11351	7.346546	-11.5965	1.20E-20	4.74E-18	36.4423	down
SLITRK6	-1.26315	5.109531	-11.5924	1.22E-20	4.74E-18	36.42155	down
CLTRN	-3.45568	7.36212	-11.5507	1.52E-20	5.78E-18	36.20928	down
TTC36	-2.96751	9.126175	-11.5198	1.78E-20	6.66E-18	36.0516	down
MSH2	1.452755	6.552897	11.46258	2.40E-20	8.80E-18	35.75985	up
MAP2K1	-1.01636	9.04653	-11.3377	4.59E-20	1.62E-17	35.12233	down
CDC37L1	-1.34905	9.08663	-11.3204	5.02E-20	1.75E-17	35.0339	down
FAHD2A	-1.08596	9.206658	-11.2742	6.38E-20	2.16E-17	34.79767	down
NUSAP1	1.875616	8.153857	11.2727	6.43E-20	2.16E-17	34.79021	up
TCTEX1D1	-1.44741	4.348333	-11.2057	9.12E-20	2.97E-17	34.44745	down
EPHA2	-1.46543	7.709844	-11.1892	9.93E-20	3.18E-17	34.36313	down
PZP	-1.60189	7.512405	-11.1792	1.05E-19	3.30E-17	34.31194	down
CDKN3	2.004518	6.80927	11.12129	1.41E-19	4.39E-17	34.01581	up
EZH2	1.655039	6.680617	11.11412	1.47E-19	4.49E-17	33.97911	up
PRC1	2.100249	6.972134	11.11108	1.49E-19	4.49E-17	33.96352	up
ECT2	2.058832	6.43369	11.08947	1.67E-19	4.95E-17	33.85291	up
CYP1A2	-3.37066	11.05442	-11.0767	1.78E-19	5.22E-17	33.78779	down
HAND2-AS	-1.41538	5.554613	-11.0553	1.99E-19	5.75E-17	33.67817	down
LIFR	-2.44975	8.247521	-11.0472	2.08E-19	5.91E-17	33.63651	down
ANLN	2.513043	5.830543	11.03291	2.24E-19	6.28E-17	33.56328	up
CENPW	1.660973	7.47368	10.95563	3.35E-19	9.27E-17	33.16739	up
GPR146	-1.11201	6.626358	-10.948	3.49E-19	9.51E-17	33.12854	down
MASP1	-1.83905	8.548333	-10.9235	3.97E-19	1.07E-16	33.00259	down
SHBG	-1.30328	7.338296	-10.8963	4.57E-19	1.21E-16	32.86339	down
CETP	-1.95149	6.908228	-10.8606	5.51E-19	1.44E-16	32.68043	down
SRPX	-2.86257	6.939256	-10.8282	6.52E-19	1.66E-16	32.51422	down
SLC25A47	-2.21191	9.148939	-10.8267	6.57E-19	1.66E-16	32.5064	down
OLFML3	-2.03909	7.273013	-10.7351	1.06E-18	2.63E-16	32.03631	down
ITGA6	1.329834	8.460536	10.69262	1.32E-18	3.22E-16	31.81852	up
HGFAC	-2.87034	7.997057	-10.6885	1.35E-18	3.25E-16	31.79748	down
UROC1	-1.20083	5.973738	-10.636	1.78E-18	4.22E-16	31.52816	down
TBC1D16	1.065095	7.409313	10.60861	2.05E-18	4.81E-16	31.38731	up
NEK2	2.332401	6.701927	10.59066	2.25E-18	5.17E-16	31.29516	up
LYVE1	-1.53822	6.611626	-10.5871	2.30E-18	5.20E-16	31.27702	down
DTL	2.215946	6.767123	10.55537	2.71E-18	6.01E-16	31.11394	up
MSRA	-1.32293	9.158688	-10.5448	2.86E-18	6.28E-16	31.05968	down
RRM2	2.247165	7.978724	10.52637	3.15E-18	6.70E-16	30.96502	up
HGF	-2.77891	6.712208	-10.5078	3.47E-18	7.23E-16	30.86954	down
GGTLC1	-1.04947	7.538685	-10.5025	3.57E-18	7.35E-16	30.84247	down
NGFR	-1.00731	6.492709	-10.4852	3.91E-18	7.97E-16	30.75377	down
ENO3	-2.22449	8.452001	-10.4819	3.98E-18	8.02E-16	30.73679	down
GJC1	1.52593	6.021165	10.44397	4.85E-18	9.69E-16	30.5418	up
IGFBP3	-2.04611	11.48131	-10.4398	4.96E-18	9.78E-16	30.52031	down
KAZN	-1.65219	7.116797	-10.4384	4.99E-18	9.78E-16	30.51331	down
RCAN1	-1.65162	10.58514	-10.4229	5.42E-18	1.04E-15	30.43338	down
RND3	-1.85481	9.957052	-10.4205	5.48E-18	1.04E-15	30.42137	down
NDC80	1.81936	5.860835	10.42034	5.49E-18	1.04E-15	30.4204	up

NAT2	-2.52333	8.683449	-10.4176	5.57E-18	1.04E-15	30.40611	down
ASS1	-1.53442	12.3098	-10.3576	7.62E-18	1.41E-15	30.09797	down
MRO	-1.49278	6.500647	-10.3419	8.27E-18	1.52E-15	30.0172	down
BUB1B	2.067398	7.011766	10.33863	8.41E-18	1.53E-15	30.00062	up
IRAK1	1.092058	9.406697	10.31993	9.28E-18	1.67E-15	29.90455	up
TPPP2	-1.39279	6.211163	-10.2882	1.09E-17	1.95E-15	29.74148	down
ZBED8	1.137725	4.719429	10.28086	1.14E-17	2.01E-15	29.70377	up
FANCC	-1.04902	7.781197	-10.2775	1.16E-17	2.03E-15	29.68633	down
CPEB3	-1.75898	7.207229	-10.2762	1.17E-17	2.03E-15	29.6796	down
CKAP2	1.401451	6.114348	10.24184	1.40E-17	2.39E-15	29.50328	up
HAMP	-4.13768	10.12131	-10.2373	1.43E-17	2.42E-15	29.48013	down
ZG16	-2.17879	8.556776	-10.2235	1.54E-17	2.56E-15	29.40896	down
STMN1	1.285926	7.453775	10.22179	1.55E-17	2.56E-15	29.40027	up
PLPP3	-1.05159	10.62617	-10.1811	1.92E-17	3.12E-15	29.19113	down
TDRKH	1.374414	5.899421	10.17824	1.95E-17	3.14E-15	29.17647	up
RFX5	1.108719	8.294972	10.17578	1.97E-17	3.16E-15	29.16384	up
SSR2	1.04154	11.07515	10.14702	2.29E-17	3.58E-15	29.0161	up
SRD5A2	-2.06404	7.789912	-10.1426	2.34E-17	3.61E-15	28.99357	down
FLVCR1	1.338567	8.673924	10.1417	2.36E-17	3.61E-15	28.98873	up
NOCT	-1.50977	5.993654	-10.1413	2.36E-17	3.61E-15	28.98665	down
UBE2T	1.686175	6.972662	10.11502	2.71E-17	4.11E-15	28.85166	up
BDH2	-1.46599	10.02972	-10.0991	2.94E-17	4.38E-15	28.77009	down
IGHM	-2.32053	8.333036	-10.0983	2.95E-17	4.38E-15	28.76577	down
FAM13A	-1.93122	9.871056	-10.0871	3.13E-17	4.61E-15	28.70815	down
JDP2	-1.34801	7.347686	-10.0827	3.21E-17	4.69E-15	28.68575	down
CD5L	-2.10328	7.416539	-10.0737	3.36E-17	4.84E-15	28.63935	down
CCNB1	2.063289	6.974535	10.05498	3.71E-17	5.27E-15	28.54315	up
AADAT	-1.7499	7.377633	-10.0111	4.66E-17	6.53E-15	28.31746	down
CXCL12	-2.79148	9.261648	-9.98553	5.33E-17	7.31E-15	28.18636	down
GSTZ1	-1.82527	9.467097	-9.9777	5.55E-17	7.57E-15	28.14612	down
SIGIRR	-1.46654	9.428297	-9.91654	7.64E-17	1.03E-14	27.83193	down
ESM1	1.726772	6.10492	9.903478	8.18E-17	1.08E-14	27.76486	up
CLRN3	-3.16318	7.742185	-9.90219	8.24E-17	1.08E-14	27.75827	down
KANK4	-2.25255	6.332806	-9.8975	8.44E-17	1.10E-14	27.73416	down
CDK1	2.046422	7.151874	9.883175	9.10E-17	1.16E-14	27.66058	up
FOXO1	-1.10983	8.80695	-9.881	9.20E-17	1.16E-14	27.64938	down
MARCO	-1.72872	7.442409	-9.87412	9.54E-17	1.18E-14	27.61408	down
TRIB1	-1.20634	11.64834	-9.86684	9.91E-17	1.22E-14	27.57668	down
CCDC34	1.424529	6.553179	9.860574	1.02E-16	1.25E-14	27.54451	up
SLC28A1	-1.1263	7.255275	-9.84922	1.09E-16	1.32E-14	27.48622	down
PODXL	1.32607	8.149624	9.844725	1.11E-16	1.34E-14	27.46311	up
ILF2	1.12739	9.550994	9.838265	1.15E-16	1.37E-14	27.42994	up
SLC9B2	-1.37023	8.084221	-9.81667	1.29E-16	1.52E-14	27.31902	down
FEZ1	-1.28351	7.329647	-9.80928	1.34E-16	1.56E-14	27.2811	down
GINS1	1.939629	6.77345	9.779163	1.57E-16	1.81E-14	27.12648	up
DNMT3L	-1.38738	7.268056	-9.77786	1.58E-16	1.81E-14	27.1198	down
RAD51AP1	1.654303	6.074613	9.759702	1.73E-16	1.97E-14	27.02657	up
PBK	2.241638	5.97886	9.757587	1.75E-16	1.98E-14	27.01571	up

PDE7B	-1.18395	6.879656	-9.74463	1.88E-16	2.08E-14	26.94918	down
CENPF	1.710729	7.207733	9.726134	2.07E-16	2.27E-14	26.85426	up
NPY1R	-1.70886	5.315339	-9.72137	2.12E-16	2.31E-14	26.82979	down
MELK	1.482846	7.361219	9.714137	2.20E-16	2.39E-14	26.79268	up
LRRN3	-1.01429	5.522124	-9.68839	2.52E-16	2.72E-14	26.66054	down
FRMD4B	-1.15261	8.745742	-9.68527	2.56E-16	2.74E-14	26.64454	down
CCNB2	1.78052	7.142028	9.682232	2.60E-16	2.77E-14	26.62895	up
UHRF1	1.877469	6.416039	9.670897	2.76E-16	2.93E-14	26.57078	up
GPD1	-1.77697	8.35364	-9.66843	2.79E-16	2.95E-14	26.5581	down
PHGDH	-1.54969	8.43669	-9.66447	2.85E-16	2.95E-14	26.53781	down
CCT3	1.013007	10.96904	9.65688	2.97E-16	3.05E-14	26.49886	up
PALM3	-1.46359	8.015279	-9.60573	3.87E-16	3.93E-14	26.23643	down
CENPU	1.828241	6.630946	9.573986	4.57E-16	4.54E-14	26.07365	up
PTTG1	1.491771	8.531507	9.565968	4.77E-16	4.70E-14	26.03253	up
STEAP3	-1.53565	9.397711	-9.56585	4.77E-16	4.70E-14	26.03192	down
SPRYD4	-1.13841	8.38589	-9.56376	4.82E-16	4.72E-14	26.02123	down
SYNE1	-1.04361	8.267131	-9.53287	5.67E-16	5.44E-14	25.86283	down
SLC19A3	-1.29018	6.757538	-9.51127	6.34E-16	6.04E-14	25.75209	down
GDPD1	1.585191	5.54865	9.498844	6.77E-16	6.38E-14	25.68841	up
KIF20A	1.904812	6.578834	9.48482	7.28E-16	6.83E-14	25.61653	up
FAM83D	1.9319	7.019946	9.472045	7.78E-16	7.20E-14	25.55107	up
CDH19	-2.20115	5.813687	-9.45281	8.60E-16	7.89E-14	25.45252	down
LY6E	-1.44457	8.215638	-9.45123	8.68E-16	7.92E-14	25.44443	down
BCO2	-2.37504	8.129761	-9.42719	9.83E-16	8.82E-14	25.32127	down
NCAPG	1.843863	6.553141	9.410165	1.07E-15	9.51E-14	25.23405	up
PCDH9	-1.9034	6.406335	-9.40785	1.09E-15	9.58E-14	25.22221	down
BBOX1	-3.34013	7.607629	-9.4048	1.11E-15	9.62E-14	25.20659	down
BIRC5	1.658474	6.838769	9.404623	1.11E-15	9.62E-14	25.20567	up
DNASE1L3	-2.25636	9.01392	-9.38781	1.21E-15	1.04E-13	25.11958	down
CHST4	-1.2604	5.706868	-9.36475	1.36E-15	1.17E-13	25.00151	down
SFRP1	-1.1557	5.703574	-9.3471	1.49E-15	1.27E-13	24.91113	down
GPC3	3.876212	9.151773	9.34083	1.54E-15	1.30E-13	24.87904	up
SGO2	1.46709	6.682541	9.328375	1.65E-15	1.38E-13	24.8153	up
MAD2L1	1.805441	6.486458	9.302618	1.88E-15	1.58E-13	24.68349	up
DIRAS3	-1.84103	5.681494	-9.29613	1.95E-15	1.62E-13	24.65029	down
DNAJC6	1.872647	6.272197	9.292307	1.99E-15	1.64E-13	24.63073	up
RFC4	1.162548	7.671337	9.272473	2.20E-15	1.79E-13	24.52927	up
ESR1	-2.32809	7.601885	-9.26255	2.32E-15	1.86E-13	24.47851	down
KPNA2	1.160234	10.35846	9.258924	2.36E-15	1.89E-13	24.45998	up
C3orf85	-2.76894	7.260176	-9.25614	2.40E-15	1.91E-13	24.44574	down
KMO	-2.14701	8.752757	-9.25344	2.43E-15	1.93E-13	24.43193	down
TCF21	-1.06056	5.80872	-9.25166	2.45E-15	1.94E-13	24.42284	down
KIF4A	1.731545	6.268776	9.245378	2.54E-15	1.99E-13	24.3907	up
FOSB	-2.46366	8.496157	-9.23041	2.74E-15	2.13E-13	24.31416	down
TAF1A	1.111895	5.457773	9.216793	2.94E-15	2.27E-13	24.24456	up
COL4A1	1.505184	10.20208	9.209026	3.06E-15	2.34E-13	24.20485	up
ASPA	-1.96584	6.903928	-9.20218	3.17E-15	2.41E-13	24.16988	down
RAB26	-1.52855	8.086368	-9.17893	3.58E-15	2.68E-13	24.05106	down

CDKN2C	1.353602	7.717217	9.117729	4.93E-15	3.57E-13	23.73843	up
LPA	-2.4278	7.865763	-9.10899	5.15E-15	3.73E-13	23.69382	down
PLIN1	-1.31608	7.548427	-9.10421	5.28E-15	3.79E-13	23.66943	down
ALDH1B1	-1.21831	7.742261	-9.10155	5.36E-15	3.83E-13	23.65584	down
ZWINT	1.709196	7.683409	9.081169	5.96E-15	4.22E-13	23.5518	up
TCIM	-1.72085	9.853106	-9.05052	6.98E-15	4.86E-13	23.3954	down
SPRY2	-1.02311	7.52833	-9.0446	7.20E-15	4.98E-13	23.36522	down
MOGAT2	-1.96957	8.587711	-9.04072	7.35E-15	5.06E-13	23.3454	down
STIL	1.156504	6.165747	9.010541	8.60E-15	5.86E-13	23.19151	up
PCLAF	1.650724	8.363797	9.002007	8.98E-15	6.10E-13	23.148	up
FOLH1B	-2.16642	7.313009	-8.99713	9.21E-15	6.20E-13	23.12312	down
DUXAP10	1.528856	7.670908	8.994147	9.36E-15	6.27E-13	23.10793	up
TRIP13	1.204998	6.374518	8.993096	9.41E-15	6.29E-13	23.10257	up
APOF	-2.72868	8.628396	-8.98531	9.80E-15	6.50E-13	23.06288	down
C1orf112	1.233774	5.936468	8.977325	1.02E-14	6.73E-13	23.02219	up
JCAD	1.25765	5.481118	8.975863	1.03E-14	6.76E-13	23.01474	up
ROBO1	1.992625	8.958863	8.949912	1.18E-14	7.66E-13	22.88251	up
CENPK	1.625443	5.420846	8.943708	1.22E-14	7.89E-13	22.8509	up
GPR158	1.630714	5.928855	8.929485	1.31E-14	8.41E-13	22.77846	up
PROZ	-1.58361	8.005305	-8.89927	1.53E-14	9.65E-13	22.62461	down
ACSM3	-2.07619	7.791137	-8.89712	1.55E-14	9.70E-13	22.61367	down
MCM3	1.004584	8.286981	8.877241	1.72E-14	1.07E-12	22.5125	up
KBTBD11	-1.8178	6.313227	-8.85665	1.91E-14	1.17E-12	22.40776	down
ENAH	1.166539	9.217081	8.842434	2.06E-14	1.25E-12	22.33543	up
RCL1	-1.52621	9.477861	-8.83617	2.12E-14	1.29E-12	22.30359	down
TBC1D31	1.078559	6.13463	8.816704	2.35E-14	1.41E-12	22.2046	up
MFSD2A	-2.35967	8.668911	-8.80208	2.53E-14	1.50E-12	22.13027	down
CENPL	1.219474	5.755098	8.77536	2.91E-14	1.70E-12	21.99449	up
LPAL2	-1.12919	5.984411	-8.76703	3.04E-14	1.76E-12	21.9522	down
FOS	-2.21084	9.570166	-8.76499	3.07E-14	1.77E-12	21.9418	down
HOTS	-1.20708	6.437551	-8.74802	3.35E-14	1.89E-12	21.85564	down
CNIH4	1.035036	10.34289	8.747684	3.36E-14	1.89E-12	21.85393	up
TM6SF2	-1.0766	7.647796	-8.73461	3.59E-14	2.02E-12	21.78757	down
TBX15	-1.66707	6.828385	-8.72753	3.72E-14	2.08E-12	21.75162	down
TKFC	-1.15558	8.340968	-8.72733	3.73E-14	2.08E-12	21.75061	down
RNF165	-1.08936	5.71995	-8.69844	4.33E-14	2.39E-12	21.60399	down
ID1	-1.89724	9.430089	-8.69363	4.44E-14	2.44E-12	21.57961	down
TUBE1	-1.49392	8.525034	-8.68796	4.57E-14	2.50E-12	21.55082	down
RAP2A	1.043296	8.594057	8.684608	4.65E-14	2.53E-12	21.53384	up
SARDH	-1.0675	7.890338	-8.67205	4.96E-14	2.68E-12	21.47017	down
TCF19	1.104463	6.124716	8.662156	5.22E-14	2.82E-12	21.41999	up
NPC1L1	-1.79999	7.923644	-8.63193	6.10E-14	3.25E-12	21.26682	down
SLC39A5	-1.38832	9.553119	-8.63002	6.16E-14	3.27E-12	21.2571	down
FCGR2B	-1.97881	6.633591	-8.61808	6.55E-14	3.43E-12	21.19666	down
TTK	1.69326	5.722221	8.597228	7.30E-14	3.78E-12	21.09103	up
ADRA1A	-1.01303	6.261202	-8.58948	7.60E-14	3.91E-12	21.05178	down
RGS5	1.452687	9.587305	8.588427	7.64E-14	3.92E-12	21.04647	up
ITGA9	-1.72644	7.7265	-8.58784	7.66E-14	3.92E-12	21.0435	down

INMT	-1.48297	7.557965	-8.56636	8.56E-14	4.32E-12	20.93476	down
HOGA1	-1.79943	8.286606	-8.565	8.62E-14	4.34E-12	20.92793	down
HELLS	1.713151	6.366602	8.554792	9.08E-14	4.54E-12	20.87626	up
STXBP6	1.38089	6.936036	8.544927	9.56E-14	4.74E-12	20.82636	up
SUCLG2	-1.02945	11.04727	-8.5298	1.03E-13	5.06E-12	20.74988	down
NAPSB	-1.20372	7.58035	-8.51212	1.13E-13	5.50E-12	20.6605	down
NUF2	1.942967	5.221726	8.50408	1.18E-13	5.69E-12	20.61985	up
CCNA2	1.203113	6.329917	8.49036	1.27E-13	6.07E-12	20.55053	up
ADIRF	-1.83311	8.54628	-8.4613	1.47E-13	6.93E-12	20.40377	down
DPT	-1.71062	7.479222	-8.45864	1.49E-13	7.01E-12	20.39036	down
PBLD	-1.7865	11.34509	-8.4422	1.62E-13	7.56E-12	20.30733	down
PLCB1	1.723687	7.864453	8.434429	1.69E-13	7.83E-12	20.26815	up
ACSL4	2.840156	7.790538	8.420001	1.82E-13	8.35E-12	20.19536	up
GCH1	-1.57443	10.24246	-8.40103	2.00E-13	9.08E-12	20.09968	down
IGF1	-2.37537	9.069163	-8.39547	2.06E-13	9.31E-12	20.07166	down
C7	-2.91485	8.790481	-8.38581	2.17E-13	9.65E-12	20.02299	down
EBF1	1.353458	6.476173	8.366367	2.39E-13	1.06E-11	19.925	up
E2F8	1.584044	5.271612	8.361781	2.45E-13	1.08E-11	19.9019	up
TTPAL	-1.20665	8.640791	-8.3517	2.58E-13	1.13E-11	19.8511	down
ANK3	-1.52921	7.57991	-8.34899	2.62E-13	1.14E-11	19.83749	down
PRIM1	1.106966	7.218306	8.338494	2.76E-13	1.19E-11	19.78463	up
JCHAIN	-3.03069	7.466859	-8.33348	2.83E-13	1.22E-11	19.75941	down
CDC20	1.617304	6.796484	8.318297	3.06E-13	1.29E-11	19.68298	up
AKR7A3	-1.43901	9.819516	-8.31821	3.07E-13	1.29E-11	19.68256	down
KLKB1	-1.71018	10.16137	-8.30029	3.36E-13	1.41E-11	19.59241	down
SULT1E1	-2.16522	6.759293	-8.28183	3.69E-13	1.53E-11	19.49957	down
PNP	-1.02881	8.603525	-8.25689	4.20E-13	1.70E-11	19.37425	down
NAAA	-1.09913	8.076218	-8.24969	4.35E-13	1.76E-11	19.33808	down
MAN1C1	-1.52753	7.659219	-8.24731	4.41E-13	1.78E-11	19.32613	down
MND1	1.276968	4.856372	8.236524	4.66E-13	1.87E-11	19.27194	up
MCM6	1.075645	8.653756	8.217063	5.15E-13	2.03E-11	19.17424	up
CRNDE	2.669921	6.115556	8.191427	5.87E-13	2.28E-11	19.04562	up
UBE2S	1.25905	8.234481	8.186176	6.03E-13	2.33E-11	19.01928	up
FANCD2	1.272077	6.065425	8.159708	6.90E-13	2.62E-11	18.8866	up
ST6GAL2	-1.46185	4.546158	-8.15561	7.05E-13	2.67E-11	18.86608	down
CYP39A1	-2.69831	8.234376	-8.13792	7.71E-13	2.90E-11	18.77746	down
CYP2C19	-1.76804	7.334271	-8.12557	8.21E-13	3.07E-11	18.71562	down
CPED1	-1.4441	8.391764	-8.1226	8.34E-13	3.10E-11	18.70076	down
IRF8	-1.04641	8.68557	-8.11467	8.68E-13	3.21E-11	18.66105	down
GCDH	-1.11975	9.362366	-8.11163	8.82E-13	3.26E-11	18.64583	down
ZFP36	-1.03434	10.77155	-8.10504	9.12E-13	3.35E-11	18.61287	down
CDCA3	1.202865	7.305189	8.099463	9.38E-13	3.43E-11	18.58496	up
THY1	1.202528	7.906224	8.085685	1.01E-12	3.65E-11	18.51605	up
EDNRB	-1.09906	8.161857	-8.07797	1.05E-12	3.78E-11	18.47747	down
TIGD1	1.129257	6.040967	8.073014	1.07E-12	3.86E-11	18.4527	up
ADAMTSL2	-1.11698	6.277304	-8.07038	1.09E-12	3.91E-11	18.43954	down
SLC35D1	-1.00156	10.35178	-8.06659	1.11E-12	3.98E-11	18.4206	down
MRC1	-1.80828	8.848743	-8.05203	1.20E-12	4.25E-11	18.34785	down

SULF2	-1.63646	8.619373	-8.05039	1.21E-12	4.28E-11	18.33965	down
FXYD1	-1.77366	8.854071	-8.04767	1.22E-12	4.31E-11	18.32607	down
HAO2	-2.63137	9.614931	-8.02776	1.35E-12	4.74E-11	18.22664	down
DLGAP5	1.558863	5.286892	8.002294	1.54E-12	5.32E-11	18.09954	up
PLSCR4	-1.11181	8.588802	-7.99406	1.61E-12	5.50E-11	18.05847	down
SMIM24	-1.972	7.397664	-7.99236	1.62E-12	5.52E-11	18.04998	down
TP53I3	1.264717	8.341486	7.987113	1.66E-12	5.65E-11	18.02383	up
CXCL2	-2.06003	10.76279	-7.98402	1.69E-12	5.70E-11	18.00841	down
CENPH	1.006003	6.056844	7.968897	1.82E-12	6.11E-11	17.93303	up
FAM151A	-1.40935	6.110142	-7.95103	2.00E-12	6.64E-11	17.84401	down
CD200	1.091105	6.283719	7.946719	2.04E-12	6.76E-11	17.82255	up
EPB41L4A	-1.59476	7.163492	-7.94056	2.11E-12	6.96E-11	17.79187	down
ST3GAL6	-1.25731	8.355624	-7.94002	2.11E-12	6.96E-11	17.7892	down
IER2	-1.06659	10.63289	-7.93473	2.17E-12	7.13E-11	17.76286	down
RBMS3	-1.64569	7.39686	-7.92857	2.24E-12	7.32E-11	17.73219	down
AURKA	1.377488	7.270655	7.911232	2.44E-12	7.88E-11	17.64593	up
LHX2	-1.55119	5.962852	-7.91082	2.45E-12	7.88E-11	17.64387	down
CIDEB	-1.30609	9.915539	-7.90732	2.49E-12	8.01E-11	17.62647	down
CYP2B6	-2.46656	9.239932	-7.90061	2.58E-12	8.24E-11	17.5931	down
RBM24	2.135607	6.423097	7.899823	2.59E-12	8.26E-11	17.58919	up
TOMM40L	1.090157	7.866738	7.899463	2.60E-12	8.26E-11	17.58739	up
ZFP1	-1.2019	7.485464	-7.88733	2.76E-12	8.71E-11	17.52706	down
STEAP4	-1.80851	7.834413	-7.87959	2.87E-12	9.03E-11	17.48861	down
CDC7	1.216368	6.0716	7.863025	3.12E-12	9.69E-11	17.40632	up
MTFR2	1.212391	5.314975	7.859105	3.18E-12	9.85E-11	17.38685	up
ACACB	-1.42375	9.90281	-7.84907	3.35E-12	1.03E-10	17.33704	down
MAMDC4	-1.11613	7.409506	-7.8159	3.96E-12	1.20E-10	17.17248	down
SOCS2	-1.65034	9.272938	-7.81434	3.99E-12	1.21E-10	17.16471	down
OTUD6B	1.07665	7.043299	7.797777	4.34E-12	1.30E-10	17.08262	up
CDC25C	1.047827	5.686945	7.789237	4.54E-12	1.35E-10	17.0403	up
ETFDH	-1.27081	8.950296	-7.78674	4.59E-12	1.36E-10	17.02792	down
LINC00598	-1.07367	6.026922	-7.78011	4.75E-12	1.40E-10	16.9951	down
RETREG1	-2.39292	8.095133	-7.76738	5.06E-12	1.49E-10	16.93205	down
LDHD	-1.21267	7.829362	-7.75323	5.44E-12	1.58E-10	16.86203	down
AGL	-1.00381	9.866446	-7.72597	6.24E-12	1.79E-10	16.72722	down
GMNN	1.26565	9.25587	7.719036	6.46E-12	1.85E-10	16.69294	up
CCNE2	1.438762	6.303214	7.707762	6.84E-12	1.93E-10	16.63724	up
FOXM1	1.151051	6.368864	7.694583	7.31E-12	2.04E-10	16.57215	up
MS4A6A	-1.30086	9.0734	-7.6876	7.57E-12	2.10E-10	16.53765	down
HSPB1	1.070354	11.05408	7.687537	7.58E-12	2.10E-10	16.53736	up
MT1G	-1.90895	12.35774	-7.67555	8.05E-12	2.22E-10	16.4782	down
LDLR	-1.18933	9.939767	-7.67045	8.26E-12	2.27E-10	16.45302	down
C1RL	-1.05404	10.21299	-7.65055	9.13E-12	2.49E-10	16.35489	down
GLS2	-2.4509	8.811603	-7.64903	9.20E-12	2.50E-10	16.34739	down
CENPE	1.095042	5.363508	7.620769	1.06E-11	2.81E-10	16.20818	up
KIF11	1.393856	4.991139	7.616194	1.08E-11	2.86E-10	16.18565	up
ANXA2	1.155929	11.60364	7.611339	1.11E-11	2.92E-10	16.16175	up
SLC38A6	1.128385	7.534712	7.608429	1.13E-11	2.96E-10	16.14742	up

IL13RA2	-1.88332	5.668598	-7.58502	1.27E-11	3.28E-10	16.03227	down
EGR1	-1.73369	10.82027	-7.56818	1.38E-11	3.53E-10	15.94949	down
ACAA1	-1.01403	11.93945	-7.55845	1.45E-11	3.69E-10	15.90169	down
FBP1	-1.80911	10.75967	-7.55665	1.46E-11	3.71E-10	15.89284	down
CYFIP2	-1.52816	8.868056	-7.54512	1.55E-11	3.90E-10	15.83623	down
OLFML2B	1.085814	5.990709	7.54351	1.56E-11	3.93E-10	15.8283	up
MT1F	-1.78263	11.88268	-7.53872	1.60E-11	4.01E-10	15.80478	down
PPID	-1.21946	8.659835	-7.53152	1.66E-11	4.15E-10	15.76945	down
FREM2	-2.37212	4.871715	-7.52811	1.69E-11	4.21E-10	15.75273	down
SLC3A1	-1.85263	6.817121	-7.51703	1.78E-11	4.42E-10	15.69838	down
MAGI2-AS	-1.43788	7.115572	-7.51669	1.79E-11	4.42E-10	15.69669	down
TEK	-1.05256	6.72173	-7.51622	1.79E-11	4.42E-10	15.6944	down
PLCXD3	-1.19816	5.996454	-7.50917	1.86E-11	4.55E-10	15.65984	down
EML6	1.213172	6.764158	7.504347	1.90E-11	4.65E-10	15.63619	up
EPHX2	-1.43582	9.037896	-7.49517	1.99E-11	4.84E-10	15.59121	down
KCNMA1	-1.2793	8.221518	-7.48873	2.06E-11	4.98E-10	15.55966	down
DCN	-2.48822	10.1829	-7.48351	2.11E-11	5.09E-10	15.53408	down
DEPDC1B	1.458145	4.797497	7.480106	2.15E-11	5.17E-10	15.51742	up
PGLYRP2	-2.19238	9.686297	-7.47432	2.21E-11	5.29E-10	15.48911	down
CYP2A7	-2.03384	10.69836	-7.45228	2.47E-11	5.81E-10	15.38123	down
EGR2	-1.62175	7.405471	-7.44103	2.61E-11	6.11E-10	15.32624	down
GHR	-2.29795	10.24882	-7.43341	2.71E-11	6.32E-10	15.28895	down
POLE2	1.378164	7.088564	7.416143	2.95E-11	6.83E-10	15.20461	up
SLC16A4	-1.73424	6.169043	-7.39646	3.26E-11	7.45E-10	15.10851	down
KIF18B	1.26274	6.672842	7.373902	3.65E-11	8.28E-10	14.99849	up
OIP5	1.089507	6.138887	7.372227	3.68E-11	8.34E-10	14.99033	up
LOC38983	1.019334	7.266422	7.354666	4.01E-11	9.06E-10	14.90475	up
PHYHD1	-1.39663	7.973933	-7.33255	4.48E-11	1.00E-09	14.79708	down
CCDC3	-1.00977	8.567128	-7.33099	4.51E-11	1.01E-09	14.78947	down
UBE2C	1.22198	8.153917	7.327459	4.59E-11	1.02E-09	14.7723	up
PRSS8	-1.31685	6.505713	-7.31971	4.77E-11	1.06E-09	14.73463	down
NR1I2	-1.84414	8.741342	-7.31659	4.85E-11	1.07E-09	14.71944	down
RBP7	1.110822	7.673833	7.308173	5.06E-11	1.11E-09	14.67851	up
GBA3	-2.48525	10.22367	-7.26623	6.23E-11	1.34E-09	14.47481	down
PANK1	-1.19692	9.572586	-7.26539	6.25E-11	1.34E-09	14.47076	down
SLC17A1	-1.21465	6.857015	-7.26316	6.32E-11	1.35E-09	14.45992	down
GABARAPL	-1.12542	9.037381	-7.26189	6.36E-11	1.36E-09	14.45375	down
TACSTD2	-2.23492	6.479401	-7.25686	6.52E-11	1.39E-09	14.42938	down
ZIC2	2.073573	5.216843	7.256623	6.53E-11	1.39E-09	14.42821	up
PDGFRA	-2.40714	8.287161	-7.25534	6.57E-11	1.40E-09	14.42199	down
SLC41A2	-1.28084	8.783234	-7.23665	7.21E-11	1.52E-09	14.33138	down
SMYD3	1.329861	7.624891	7.229618	7.46E-11	1.57E-09	14.29733	up
CYP4V2	-1.19739	9.735582	-7.22371	7.69E-11	1.61E-09	14.26874	down
HMGB2	1.012843	9.713542	7.222547	7.73E-11	1.62E-09	14.26309	up
TENM1	-2.44318	7.227001	-7.2225	7.73E-11	1.62E-09	14.26287	down
SERPINI1	1.236481	6.366948	7.210756	8.20E-11	1.70E-09	14.20601	up
MT1H	-1.64141	12.19467	-7.20687	8.35E-11	1.73E-09	14.1872	down
GCKR	-1.12624	9.475975	-7.20629	8.38E-11	1.73E-09	14.18441	down

CA2	-1.28541	9.80094	-7.20361	8.49E-11	1.75E-09	14.17145	down
PPP1R3B	-1.58326	10.05943	-7.18911	9.12E-11	1.87E-09	14.10129	down
GREM2	-1.71815	6.512178	-7.18579	9.27E-11	1.89E-09	14.08526	down
DUSP6	-1.02188	10.82428	-7.18257	9.42E-11	1.92E-09	14.0697	down
NR0B2	-1.14746	9.770149	-7.1777	9.65E-11	1.96E-09	14.04618	down
CDC6	1.050241	5.182184	7.160371	1.05E-10	2.11E-09	13.96247	up
NR3C2	-1.01693	7.237405	-7.13893	1.17E-10	2.32E-09	13.85901	down
IGKC	-1.92917	9.008365	-7.12957	1.22E-10	2.42E-09	13.81389	down
ATF5	-1.83769	10.51887	-7.12852	1.23E-10	2.43E-09	13.8088	down
SKAP1	-1.11382	7.874429	-7.11743	1.30E-10	2.55E-09	13.75537	down
AKR1C3	1.110889	12.19626	7.113778	1.32E-10	2.59E-09	13.73778	up
KIF2C	1.101314	6.577433	7.108583	1.36E-10	2.65E-09	13.71276	up
GPR180	-1.03627	6.549322	-7.09905	1.42E-10	2.76E-09	13.66684	down
CDKN2B	1.190526	5.351238	7.093851	1.46E-10	2.82E-09	13.64184	up
ANXA10	-1.83253	8.922676	-7.09362	1.46E-10	2.82E-09	13.6407	down
LAMC1	1.086938	9.639194	7.077835	1.58E-10	3.02E-09	13.5648	up
GLDC	-1.32881	10.61695	-7.05536	1.76E-10	3.35E-09	13.45679	down
SQLE	1.715208	8.578501	7.052715	1.79E-10	3.38E-09	13.44409	up
ATF3	-1.27945	9.434082	-7.0381	1.92E-10	3.60E-09	13.37396	down
FTCD	-1.34607	10.29511	-7.02876	2.01E-10	3.76E-09	13.32912	down
PROM1	-1.64562	5.264112	-7.01351	2.16E-10	4.03E-09	13.25601	down
GADD45B	-1.27325	10.46968	-7.01239	2.18E-10	4.05E-09	13.25065	down
NR4A3	-1.41796	6.966459	-7.00849	2.22E-10	4.10E-09	13.23196	down
GLYAT	-2.20737	9.984517	-7.00558	2.25E-10	4.15E-09	13.21803	down
MT1M	-2.85969	8.830849	-7.00179	2.29E-10	4.22E-09	13.19988	down
ST8SIA6-A	1.642787	6.662355	6.986548	2.47E-10	4.52E-09	13.12689	up
ECRG4	-1.03652	5.572871	-6.98269	2.52E-10	4.59E-09	13.10843	down
PLIN2	-1.13802	11.48652	-6.98265	2.52E-10	4.59E-09	13.10823	down
KNL1	1.004174	8.050094	6.953929	2.90E-10	5.19E-09	12.97092	up
FAM149A	-1.36029	8.192823	-6.94972	2.96E-10	5.28E-09	12.95082	down
DLG5	1.076164	8.239015	6.942245	3.07E-10	5.45E-09	12.91512	up
TMEM45A	-1.67436	9.540957	-6.93146	3.23E-10	5.72E-09	12.86361	down
SYTL5	-1.14757	6.346595	-6.92434	3.35E-10	5.91E-09	12.82966	down
APOBEC3B	1.640361	5.587079	6.921987	3.39E-10	5.97E-09	12.81844	up
C3P1	-2.09606	10.29701	-6.91806	3.45E-10	6.06E-09	12.79973	down
CD1D	-1.42905	7.017914	-6.91499	3.51E-10	6.15E-09	12.78508	down
IGLC1	-2.29213	10.70001	-6.91393	3.52E-10	6.17E-09	12.78004	down
AGXT2	-1.52539	9.101881	-6.89462	3.87E-10	6.67E-09	12.688	down
PSAT1	-1.20495	10.90812	-6.88613	4.03E-10	6.92E-09	12.64761	down
C1orf162	-1.03776	7.948825	-6.88403	4.08E-10	6.94E-09	12.63761	down
CYP4A11	-1.93618	10.11178	-6.87785	4.20E-10	7.12E-09	12.60822	down
EDIL3	1.865624	7.469627	6.87526	4.25E-10	7.19E-09	12.59588	up
MNS1	1.49397	5.583463	6.873231	4.30E-10	7.24E-09	12.58622	up
SLC17A3	-1.44521	8.136045	-6.85447	4.71E-10	7.84E-09	12.49701	down
MT1HL1	-1.27849	12.85295	-6.85159	4.77E-10	7.93E-09	12.48333	down
SLCO1B3	-2.93968	9.014517	-6.84905	4.83E-10	8.01E-09	12.47128	down
IL1RAP	-1.18685	9.310568	-6.83628	5.14E-10	8.46E-09	12.41066	down
IGLL3P	-1.36025	8.434302	-6.82953	5.31E-10	8.68E-09	12.37862	down

EPB41L4B	-1.10863	10.32495	-6.82497	5.43E-10	8.82E-09	12.35699	down
SERPINE1	-1.59966	9.178807	-6.81865	5.60E-10	9.06E-09	12.32698	down
SPC25	1.276979	4.847638	6.81639	5.66E-10	9.13E-09	12.31628	up
CEP55	1.247873	4.955928	6.816384	5.66E-10	9.13E-09	12.31625	up
OSBPL3	1.029041	6.405553	6.814125	5.73E-10	9.22E-09	12.30555	up
SPINK1	3.679947	9.411207	6.79759	6.20E-10	9.88E-09	12.2272	up
TRPM8	-1.9318	8.725854	-6.796	6.25E-10	9.94E-09	12.21965	down
FBLN5	-1.2572	7.26034	-6.78694	6.53E-10	1.03E-08	12.17677	down
SULT1A2	-1.02518	10.12692	-6.7797	6.77E-10	1.07E-08	12.14248	down
RNF125	-1.2667	8.107884	-6.77517	6.92E-10	1.08E-08	12.12106	down
PIK3C2G	-1.25544	6.838102	-6.76982	7.10E-10	1.11E-08	12.09577	down
MT1E	-1.55819	12.4882	-6.75845	7.50E-10	1.17E-08	12.042	down
CNTN4	-1.12459	5.561351	-6.7454	7.99E-10	1.23E-08	11.98037	down
ANGPTL1	-1.3815	6.106231	-6.73762	8.29E-10	1.27E-08	11.94367	down
AQP3	-1.12388	9.874926	-6.73506	8.40E-10	1.28E-08	11.93157	down
CHML	1.261188	7.672814	6.722017	8.94E-10	1.35E-08	11.87004	up
SERPINB9	-1.02038	8.327506	-6.72132	8.97E-10	1.35E-08	11.86674	down
SLC7A2	-1.4544	10.76464	-6.71639	9.19E-10	1.38E-08	11.84352	down
CA5A	-1.0776	8.270856	-6.69688	1.01E-09	1.51E-08	11.75158	down
CTHRC1	2.386531	6.590453	6.692295	1.03E-09	1.54E-08	11.73001	up
DHRS1	-1.09462	10.43164	-6.67771	1.11E-09	1.64E-08	11.66141	down
EXOC3L4	-1.1497	8.044948	-6.6729	1.13E-09	1.67E-08	11.63875	down
COMT	-1.07224	10.13586	-6.6718	1.14E-09	1.67E-08	11.63361	down
CDKN1C	-1.00295	7.264796	-6.66694	1.17E-09	1.71E-08	11.61076	down
FGL2	-1.07216	8.813553	-6.65779	1.22E-09	1.77E-08	11.56778	down
THRSP	-2.99185	9.402669	-6.64897	1.27E-09	1.84E-08	11.52633	down
PPBP	-1.04224	4.243037	-6.63472	1.36E-09	1.96E-08	11.45948	down
FAS	-1.09494	7.574808	-6.63118	1.39E-09	1.99E-08	11.44289	down
GAS2L3	1.234272	7.464925	6.629438	1.40E-09	2.00E-08	11.43471	up
OGDHL	-1.62699	9.250756	-6.62937	1.40E-09	2.00E-08	11.43441	down
RDH16	-1.95359	10.49473	-6.62404	1.43E-09	2.04E-08	11.40942	down
COL4A2	1.099837	10.21563	6.621508	1.45E-09	2.06E-08	11.39754	up
SLC22A1	-2.54777	10.24182	-6.60808	1.55E-09	2.18E-08	11.33465	down
CCN1	-1.52519	10.21635	-6.60474	1.57E-09	2.21E-08	11.31899	down
OAT	-1.70303	9.952962	-6.60274	1.59E-09	2.23E-08	11.30962	down
MT1X	-1.46542	12.79888	-6.59256	1.67E-09	2.33E-08	11.26199	down
AGBL3	1.075192	4.609943	6.588755	1.70E-09	2.37E-08	11.2442	up
KCND3	-1.72074	7.672886	-6.58778	1.71E-09	2.38E-08	11.23963	down
TFPI2	-1.57287	5.985037	-6.58614	1.72E-09	2.39E-08	11.23198	down
SERPINA4	-1.49786	10.47055	-6.58462	1.73E-09	2.41E-08	11.22484	down
NNMT	-1.91193	12.24155	-6.57525	1.81E-09	2.51E-08	11.18105	down
SIPA1L2	1.241043	8.061329	6.573446	1.83E-09	2.52E-08	11.17263	up
TREH	-1.07378	5.832889	-6.5671	1.88E-09	2.59E-08	11.14298	down
SHCBP1	1.099577	5.153766	6.560127	1.95E-09	2.66E-08	11.11042	up
KCNJ16	-1.3923	4.248021	-6.55038	2.04E-09	2.76E-08	11.06492	down
LRRC1	1.290854	6.501865	6.54762	2.07E-09	2.79E-08	11.05206	up
CLYBL	-1.09235	8.74772	-6.52926	2.26E-09	3.02E-08	10.96649	down
N4BP2L1	-1.04581	9.997155	-6.51774	2.39E-09	3.17E-08	10.91283	down

IGF2BP3	2.209101	5.994749	6.512839	2.44E-09	3.23E-08	10.89	up
CYP2A6	-2.33829	11.32218	-6.49732	2.63E-09	3.44E-08	10.81781	down
MCC	-1.46221	8.481781	-6.47396	2.94E-09	3.79E-08	10.70928	down
ZFPM2	-1.16783	5.522304	-6.46245	3.10E-09	3.98E-08	10.65589	down
MT2A	-1.17717	13.20669	-6.46234	3.11E-09	3.98E-08	10.65534	down
ATAD2	1.076787	7.742338	6.455329	3.21E-09	4.10E-08	10.62284	up
MYO10	-1.22819	7.668864	-6.4544	3.23E-09	4.12E-08	10.61854	down
GADD45A	-1.01925	10.8957	-6.44942	3.30E-09	4.20E-08	10.59547	down
AKR1D1	-2.5679	10.29882	-6.44638	3.35E-09	4.26E-08	10.58138	down
CYP2C18	-1.61145	9.733813	-6.44085	3.44E-09	4.36E-08	10.55575	down
DEPDC1	1.08902	5.508575	6.436185	3.52E-09	4.45E-08	10.53414	up
C1R	-1.18191	12.02424	-6.43514	3.53E-09	4.47E-08	10.52932	down
DEPDC7	-1.3883	9.179885	-6.41049	3.97E-09	4.97E-08	10.41526	down
PRODH2	-1.05199	9.849821	-6.40467	4.08E-09	5.10E-08	10.38837	down
FABP5	1.318668	8.435437	6.395754	4.26E-09	5.28E-08	10.34719	up
TXNRD1	1.063992	10.08226	6.39564	4.26E-09	5.28E-08	10.34666	up
TPX2	1.063656	6.92237	6.376177	4.67E-09	5.72E-08	10.25685	up
AKR1B10	3.041232	9.858827	6.37607	4.68E-09	5.72E-08	10.25635	up
CNTN3	-2.46521	6.288584	-6.37503	4.70E-09	5.74E-08	10.25157	down
GSPT2	-1.5525	7.207061	-6.37435	4.72E-09	5.75E-08	10.24843	down
PTGS2	-1.5944	5.056729	-6.36457	4.94E-09	5.98E-08	10.20332	down
SOX6	-1.18008	7.426835	-6.36234	4.99E-09	6.03E-08	10.19306	down
TKT	1.01299	9.84749	6.353687	5.20E-09	6.25E-08	10.15322	up
MCM4	1.055132	7.549891	6.33403	5.70E-09	6.83E-08	10.06278	up
WDR72	-2.04564	8.960091	-6.32511	5.95E-09	7.09E-08	10.02178	down
SERPINF2	-1.05788	10.71182	-6.32168	6.05E-09	7.20E-08	10.00602	down
PTGIS	-1.41017	7.521514	-6.32058	6.08E-09	7.23E-08	10.00096	down
CLDN10	-1.64799	6.097081	-6.30477	6.55E-09	7.71E-08	9.928382	down
LIPC	-1.21138	9.950073	-6.29869	6.74E-09	7.91E-08	9.900511	down
ACADL	-1.57386	7.47091	-6.29788	6.76E-09	7.93E-08	9.896769	down
PHLDA1	-1.38777	9.67854	-6.29332	6.91E-09	8.09E-08	9.875877	down
PYROXD2	-1.15302	7.422093	-6.29116	6.98E-09	8.16E-08	9.865982	down
F11	-1.17499	9.540742	-6.28022	7.35E-09	8.55E-08	9.815868	down
CD109	1.466121	6.792129	6.275885	7.50E-09	8.70E-08	9.795995	up
PRG4	-1.8168	6.933193	-6.27354	7.59E-09	8.79E-08	9.78524	down
HBB	-1.38047	10.69965	-6.25814	8.15E-09	9.37E-08	9.714805	down
C8A	-1.52372	10.72935	-6.25143	8.41E-09	9.66E-08	9.684127	down
CYP3A43	-1.24783	8.780817	-6.25084	8.44E-09	9.67E-08	9.681416	down
IYD	-1.32644	6.425593	-6.24852	8.53E-09	9.76E-08	9.670825	down
BCHE	-2.28617	9.713246	-6.24551	8.65E-09	9.88E-08	9.657084	down
GBP1	-1.07971	10.17854	-6.24461	8.69E-09	9.91E-08	9.65296	down
CYP2J2	-1.01414	9.507806	-6.2443	8.70E-09	9.92E-08	9.651559	down
CYP2C9	-1.77563	12.19088	-6.22165	9.68E-09	1.09E-07	9.548155	down
CCL2	-1.59208	8.598531	-6.20859	1.03E-08	1.15E-07	9.488645	down
CALHM6	-1.36721	8.069133	-6.20858	1.03E-08	1.15E-07	9.488581	down
NAMPT	-1.30087	10.57642	-6.19832	1.08E-08	1.20E-07	9.441865	down
APOA5	-1.63347	10.17005	-6.19793	1.08E-08	1.20E-07	9.440104	down
RBP5	-1.24098	9.224798	-6.19378	1.10E-08	1.22E-07	9.421199	down

FOXP2	-1.19814	6.853936	-6.19071	1.12E-08	1.23E-07	9.407222	down
GYS2	-2.33833	9.065077	-6.18133	1.17E-08	1.28E-07	9.364571	down
TRIM16	1.396131	6.730226	6.172127	1.22E-08	1.33E-07	9.322745	up
GRAMD1C	-1.50627	7.930375	-6.16715	1.25E-08	1.36E-07	9.300138	down
MIR99AHG	-1.10085	6.337409	-6.16341	1.27E-08	1.37E-07	9.283157	down
BAG2	1.084022	7.387402	6.157601	1.31E-08	1.41E-07	9.256782	up
LINC01554	-1.71745	7.334985	-6.1458	1.38E-08	1.48E-07	9.203273	down
LOC10192{	-1.19736	7.108929	-6.13383	1.46E-08	1.55E-07	9.149023	down
LEF1	1.379587	7.089391	6.122379	1.54E-08	1.63E-07	9.09715	up
VNN1	-2.26002	10.29925	-6.08178	1.86E-08	1.93E-07	8.913696	down
ABI3BP	-1.72321	6.534693	-6.05341	2.12E-08	2.18E-07	8.785865	down
AFM	-1.86582	10.27624	-6.03688	2.29E-08	2.33E-07	8.711475	down
BASP1	-1.21068	8.042121	-6.03416	2.32E-08	2.35E-07	8.699265	down
DAO	-1.18081	8.822031	-6.03055	2.35E-08	2.39E-07	8.683036	down
FYB2	-1.34968	8.505347	-6.03053	2.35E-08	2.39E-07	8.682966	down
HABP2	-1.27668	10.65956	-6.02759	2.39E-08	2.41E-07	8.669714	down
GPT2	-1.18868	8.350786	-6.01664	2.51E-08	2.52E-07	8.620588	down
SLC46A3	-1.44267	9.518321	-6.01599	2.52E-08	2.53E-07	8.617639	down
COLEC11	-1.42971	9.833672	-6.00463	2.65E-08	2.65E-07	8.566682	down
TSPAN12	-1.11056	8.2628	-5.99218	2.81E-08	2.80E-07	8.510894	down
PRKAA2	1.4068	7.165765	5.981171	2.96E-08	2.93E-07	8.461612	up
CCDC198	-1.23869	8.390997	-5.96374	3.20E-08	3.15E-07	8.383666	down
CYP3A4	-1.86978	12.09135	-5.96314	3.21E-08	3.15E-07	8.380976	down
NMRAL2P	1.730905	5.571333	5.953388	3.36E-08	3.29E-07	8.337429	up
ACSL1	-1.07715	11.79469	-5.93854	3.60E-08	3.50E-07	8.27116	down
ID4	-1.5725	7.288458	-5.93308	3.69E-08	3.58E-07	8.246829	down
CFTR	-1.52625	6.111648	-5.92201	3.88E-08	3.73E-07	8.197518	down
LINC01146	-1.11526	6.138785	-5.92161	3.89E-08	3.73E-07	8.195735	down
CPN2	-1.29167	8.73779	-5.92042	3.91E-08	3.75E-07	8.190422	down
SLC22A4	1.068244	5.214036	5.908803	4.12E-08	3.93E-07	8.138741	up
SDS	-1.82348	10.32987	-5.90129	4.27E-08	4.05E-07	8.105323	down
GABRP	-1.13013	4.7969	-5.89741	4.34E-08	4.11E-07	8.08811	down
SFN	1.030972	8.885283	5.887769	4.54E-08	4.27E-07	8.045265	up
SLC25A18	-1.42285	9.624016	-5.87363	4.84E-08	4.52E-07	7.982528	down
CDA	-1.20429	7.395343	-5.87167	4.88E-08	4.55E-07	7.97385	down
SULT1C2	2.123553	6.75024	5.867078	4.99E-08	4.64E-07	7.95348	up
PDE11A	-1.46464	6.659751	-5.84984	5.39E-08	4.96E-07	7.877134	down
SSX1	1.92535	6.363357	5.843456	5.55E-08	5.09E-07	7.848897	up
CCL4	-1.04611	8.245631	-5.84333	5.56E-08	5.09E-07	7.84834	down
DUSP5	-1.04985	8.309497	-5.83475	5.78E-08	5.29E-07	7.810387	down
IL33	-1.07582	6.11727	-5.82348	6.08E-08	5.52E-07	7.760643	down
PRKAR2B	-1.1421	5.603262	-5.81795	6.24E-08	5.65E-07	7.736224	down
TDO2	-1.53152	11.82564	-5.80792	6.53E-08	5.88E-07	7.691987	down
CCL5	-1.26317	8.076532	-5.79774	6.84E-08	6.12E-07	7.647119	down
MBL2	-1.97892	9.762608	-5.782	7.34E-08	6.53E-07	7.577806	down
CRISPLD2	-1.37968	7.877572	-5.7766	7.52E-08	6.67E-07	7.554059	down
SEMA6D	-1.00936	6.122493	-5.77643	7.53E-08	6.67E-07	7.553325	down
VSIG4	-1.00547	7.811564	-5.76824	7.81E-08	6.91E-07	7.517347	down

KYNU	-1.20694	9.503677	-5.75365	8.35E-08	7.33E-07	7.453252	down
SAMD5	-1.42675	7.188309	-5.73646	9.02E-08	7.85E-07	7.377897	down
SLC17A2	-1.48078	9.18823	-5.73445	9.10E-08	7.91E-07	7.369119	down
ADAMTSL3	-1.09911	7.144255	-5.72638	9.44E-08	8.17E-07	7.333784	down
BGN	-1.43454	9.073187	-5.72211	9.63E-08	8.30E-07	7.315069	down
THBS1	-1.57667	9.932535	-5.71866	9.78E-08	8.41E-07	7.299975	down
TMEM154	-1.17046	6.194709	-5.69828	1.07E-07	9.11E-07	7.210929	down
C9	-2.78745	10.81303	-5.68814	1.12E-07	9.47E-07	7.166691	down
ALDH8A1	-1.54626	11.26873	-5.68395	1.14E-07	9.64E-07	7.148433	down
ADAM9	1.01892	8.409735	5.669675	1.22E-07	1.02E-06	7.086243	up
SPIDR	-1.11004	8.139749	-5.6693	1.22E-07	1.02E-06	7.084616	down
CCL21	-1.00394	7.427032	-5.66733	1.23E-07	1.03E-06	7.076054	down
FXYD2	-1.35608	7.733912	-5.656	1.30E-07	1.08E-06	7.026748	down
PLAG1	1.001455	4.897075	5.637189	1.41E-07	1.16E-06	6.945028	up
MAP2	1.613589	8.095155	5.635785	1.42E-07	1.16E-06	6.938935	up
ARRDC4	-1.15648	8.668879	-5.62763	1.47E-07	1.20E-06	6.903541	down
HRCT1	1.050513	7.569931	5.626997	1.48E-07	1.21E-06	6.900814	up
NR4A2	-1.37819	7.942389	-5.60622	1.62E-07	1.31E-06	6.81083	down
ADAMTS1	-1.00593	8.609453	-5.59967	1.67E-07	1.35E-06	6.782494	down
PLGLB2	-1.05571	6.474053	-5.58706	1.76E-07	1.42E-06	6.727968	down
CYP2B7P	-1.81907	8.29625	-5.57314	1.88E-07	1.50E-06	6.667885	down
GJB2	-1.10304	8.296602	-5.56239	1.97E-07	1.56E-06	6.621542	down
FAM110C	-1.47965	8.61122	-5.54959	2.08E-07	1.65E-06	6.566425	down
SLC25A15	-1.02414	10.32282	-5.54705	2.11E-07	1.67E-06	6.555486	down
SOCS3	-1.44807	8.101696	-5.54603	2.12E-07	1.67E-06	6.551103	down
ADH6	-1.31474	11.17846	-5.54389	2.14E-07	1.69E-06	6.541911	down
CTH	-1.66343	10.74174	-5.5355	2.22E-07	1.74E-06	6.505859	down
HYAL1	-1.1189	9.738799	-5.51242	2.46E-07	1.91E-06	6.406753	down
CYP4F2	-1.25514	10.15273	-5.51105	2.47E-07	1.92E-06	6.40091	down
KLF4	-1.30347	8.460124	-5.50562	2.53E-07	1.96E-06	6.377621	down
DAB1	-1.01583	6.456187	-5.50439	2.55E-07	1.97E-06	6.372364	down
MXRA5	-1.25838	7.757369	-5.49993	2.60E-07	2.00E-06	6.353238	down
DNAJC12	-1.54564	8.758612	-5.49153	2.69E-07	2.07E-06	6.317288	down
SPP2	-1.71299	10.34359	-5.48378	2.79E-07	2.13E-06	6.28414	down
SORL1	-1.1031	10.57519	-5.46323	3.05E-07	2.32E-06	6.196367	down
XDH	-1.53807	10.19303	-5.44997	3.24E-07	2.44E-06	6.139871	down
THBS4	1.125988	6.828093	5.445517	3.30E-07	2.48E-06	6.120891	up
CD163	-1.05062	8.970101	-5.42909	3.55E-07	2.65E-06	6.050988	down
CCL19	-1.45165	7.476425	-5.42625	3.59E-07	2.68E-06	6.038908	down
DTNA	1.18887	7.2322	5.422309	3.66E-07	2.72E-06	6.022179	up
TAT	-1.31588	9.372645	-5.42219	3.66E-07	2.72E-06	6.021679	down
ART4	-1.34295	8.186777	-5.4043	3.96E-07	2.91E-06	5.945758	down
PCK2	-1.04457	10.87416	-5.38249	4.35E-07	3.16E-06	5.853388	down
AVPR1A	-1.97115	7.928875	-5.37573	4.48E-07	3.25E-06	5.82479	down
C11orf96	-1.30076	9.793625	-5.37261	4.54E-07	3.29E-06	5.81161	down
C6	-1.58954	11.20616	-5.36484	4.70E-07	3.38E-06	5.778763	down
LINC00844	-2.39202	9.003674	-5.34986	5.02E-07	3.59E-06	5.715575	down
ACADSB	-1.07954	10.18498	-5.34449	5.14E-07	3.66E-06	5.692963	down

ABCA8	-1.66154	9.102866	-5.33804	5.28E-07	3.76E-06	5.665759	down
SRD5A1	-1.02175	9.507895	-5.33147	5.44E-07	3.86E-06	5.638112	down
CD69	-1.27292	6.185822	-5.29434	6.39E-07	4.47E-06	5.48221	down
SLC6A12	-1.07006	8.172755	-5.2915	6.47E-07	4.52E-06	5.470336	down
LECT2	-1.77889	11.30348	-5.28195	6.74E-07	4.70E-06	5.430336	down
CFHR3	-1.83619	10.45821	-5.27867	6.84E-07	4.76E-06	5.416609	down
UGT2B28	-1.0888	12.19061	-5.2699	7.10E-07	4.93E-06	5.379922	down
ISX	1.189621	5.584868	5.266256	7.22E-07	4.99E-06	5.364716	up
TSLP	-1.35362	6.179611	-5.25869	7.46E-07	5.15E-06	5.333105	down
ACOT12	-1.71016	8.763913	-5.25814	7.47E-07	5.15E-06	5.330833	down
ANG	-1.11688	12.17591	-5.25207	7.67E-07	5.27E-06	5.305506	down
POU2AF1	-1.27886	6.271588	-5.2403	8.07E-07	5.53E-06	5.256445	down
GIPC2	-1.19297	7.491641	-5.22911	8.47E-07	5.77E-06	5.209881	down
SPP1	2.070422	10.17735	5.21949	8.83E-07	5.98E-06	5.169864	up
PLXNC1	1.205497	7.205273	5.210663	9.17E-07	6.19E-06	5.133201	up
ANO1	-1.53516	7.976767	-5.1903	1.00E-06	6.68E-06	5.048748	down
CCL20	1.954948	8.479533	5.170452	1.09E-06	7.19E-06	4.96665	up
ALKAL2	-1.27592	5.134844	-5.15428	1.17E-06	7.65E-06	4.899896	down
ANKRD29	1.188324	7.042638	5.143518	1.22E-06	7.97E-06	4.855526	up
FGF13	1.368003	6.626381	5.113648	1.39E-06	8.93E-06	4.732692	up
STK39	1.207285	6.637629	5.108052	1.42E-06	9.09E-06	4.709729	up
EVA1A	-1.00522	9.731539	-5.09952	1.47E-06	9.39E-06	4.674733	down
LINC01419	2.06828	4.591202	5.078949	1.61E-06	1.02E-05	4.590543	up
ZSCAN31	1.038782	6.683613	5.074707	1.64E-06	1.03E-05	4.573207	up
PLA1A	-1.08156	8.959937	-5.07204	1.66E-06	1.04E-05	4.562327	down
PTPRD	-1.06832	7.037488	-5.04697	1.84E-06	1.15E-05	4.460047	down
ADH4	-2.13327	11.92622	-5.04252	1.88E-06	1.16E-05	4.441959	down
SLC27A5	-1.29843	11.09667	-5.04163	1.89E-06	1.17E-05	4.43832	down
LAMA3	1.179355	7.195258	5.037218	1.92E-06	1.19E-05	4.42037	up
LCN2	1.841043	8.344517	5.031888	1.96E-06	1.21E-05	4.398697	up
GAS1	-1.30512	6.449812	-5.01425	2.12E-06	1.29E-05	4.327065	down
LOC15727:	-1.05009	5.544056	-5.00694	2.18E-06	1.33E-05	4.297433	down
GNAL	1.002582	5.98366	4.982379	2.42E-06	1.45E-05	4.198057	up
ARMCX3	-1.13192	8.00384	-4.9816	2.43E-06	1.46E-05	4.194902	down
COX7B2	1.774069	5.351459	4.968046	2.57E-06	1.54E-05	4.140202	up
CP	-1.03262	12.25518	-4.96628	2.59E-06	1.55E-05	4.133075	down
GNMT	-1.60173	9.806529	-4.95677	2.69E-06	1.60E-05	4.094769	down
SLC4A4	-1.29768	8.485759	-4.95645	2.70E-06	1.60E-05	4.093482	down
C8B	-1.121	11.18643	-4.94553	2.82E-06	1.67E-05	4.049506	down
SMPX	1.30817	5.677075	4.922317	3.11E-06	1.81E-05	3.956311	up
PPARGC1A	-1.17379	8.195611	-4.90408	3.36E-06	1.95E-05	3.883269	down
SLC16A14	-1.10895	6.4314	-4.88115	3.69E-06	2.12E-05	3.791668	down
EGR3	-1.0256	6.086738	-4.85891	4.05E-06	2.30E-05	3.703103	down
HAL	-1.9208	10.09853	-4.79831	5.19E-06	2.86E-05	3.463044	down
SH3YL1	-1.0875	8.506394	-4.79601	5.24E-06	2.89E-05	3.453983	down
B3GNT5	1.051441	7.178866	4.788064	5.42E-06	2.97E-05	3.422655	up
RMST	1.136096	4.926449	4.786509	5.45E-06	2.98E-05	3.41653	up
MAGEA12	1.578721	4.962152	4.785023	5.49E-06	3.00E-05	3.41068	up

PPP1R1A	-1.46083	9.145146	-4.77245	5.78E-06	3.14E-05	3.361187	down
CYTIP	-1.06238	6.48216	-4.76638	5.92E-06	3.21E-05	3.337336	down
FAM133A	1.447395	5.36351	4.765818	5.94E-06	3.21E-05	3.335143	up
BHMT	-1.45405	11.70157	-4.75865	6.11E-06	3.29E-05	3.307007	down
EP300-AS1	-1.04312	7.385265	-4.75178	6.29E-06	3.38E-05	3.28006	down
TRIM22	-1.00225	9.250467	-4.74237	6.53E-06	3.50E-05	3.243171	down
GREB1	1.20899	6.241025	4.737804	6.65E-06	3.56E-05	3.225312	up
REEP6	-1.09939	10.26416	-4.7347	6.74E-06	3.60E-05	3.21316	down
GEM	-1.18812	7.407626	-4.72674	6.96E-06	3.72E-05	3.182072	down
HSD17B2	-1.17756	10.17898	-4.7264	6.97E-06	3.72E-05	3.180738	down
CDH1	-1.11032	10.12035	-4.7083	7.50E-06	3.97E-05	3.110091	down
MFAP3L	-1.33625	8.511426	-4.70621	7.57E-06	4.00E-05	3.101939	down
MLIP	-1.13002	8.120532	-4.70477	7.61E-06	4.01E-05	3.09636	down
XK	1.3831	5.171208	4.703585	7.65E-06	4.03E-05	3.091729	up
FNDC5	-1.31404	8.295094	-4.69402	7.95E-06	4.17E-05	3.05449	down
S100A8	-1.36588	7.934962	-4.69346	7.97E-06	4.17E-05	3.052328	down
MAGEA1	1.424296	6.409588	4.692221	8.01E-06	4.19E-05	3.047508	up
MYH4	1.022117	3.95198	4.688578	8.13E-06	4.24E-05	3.033346	up
TRIM71	1.343812	5.403698	4.679393	8.44E-06	4.39E-05	2.997672	up
MAGEA6	2.188459	5.669019	4.673106	8.65E-06	4.48E-05	2.973281	up
COL8A1	1.165716	5.613976	4.661185	9.08E-06	4.68E-05	2.927092	up
LINC01831	-1.11918	6.498633	-4.66087	9.09E-06	4.68E-05	2.925861	down
ETNPPL	-1.59824	10.27279	-4.64913	9.53E-06	4.88E-05	2.880452	down
COL4A3	-1.02732	5.629917	-4.64895	9.54E-06	4.89E-05	2.879749	down
PCDHB14	1.063599	5.076745	4.641266	9.84E-06	5.03E-05	2.850087	up
INHBE	-1.36007	9.315739	-4.63964	9.91E-06	5.06E-05	2.843794	down
SLC22A7	-1.51529	10.72142	-4.61969	1.07E-05	5.44E-05	2.766925	down
SPARCL1	1.07469	9.661952	4.619589	1.07E-05	5.44E-05	2.766532	up
CFHR4	-1.54737	11.08358	-4.59434	1.19E-05	5.96E-05	2.66954	down
ITIH4	-1.09546	12.19371	-4.58244	1.25E-05	6.21E-05	2.623954	down
FAM169A	1.010989	8.11993	4.575651	1.28E-05	6.36E-05	2.597975	up
PWWP3B	-1.09762	5.460252	-4.57318	1.29E-05	6.42E-05	2.588516	down
ARHGEF26	-1.00104	9.178397	-4.5721	1.30E-05	6.44E-05	2.584399	down
CYP2C8	-1.69561	12.22852	-4.55934	1.37E-05	6.74E-05	2.535661	down
COL14A1	-1.2827	6.657319	-4.53382	1.51E-05	7.38E-05	2.438521	down
PDZRN3	-1.06989	5.169303	-4.52918	1.54E-05	7.50E-05	2.420878	down
PIGR	-1.39711	9.433177	-4.52686	1.56E-05	7.57E-05	2.412064	down
DMGDH	-1.20258	9.064274	-4.52116	1.59E-05	7.71E-05	2.390449	down
ADH1A	-1.40173	12.10421	-4.51951	1.60E-05	7.76E-05	2.384179	down
SLC7A11	1.235729	6.007896	4.518377	1.61E-05	7.78E-05	2.379878	up
CXCL6	-1.65204	5.986747	-4.50618	1.69E-05	8.12E-05	2.333686	down
HPGD	-1.7742	9.151589	-4.50346	1.71E-05	8.19E-05	2.323376	down
SLC27A2	-1.15797	10.23841	-4.4884	1.81E-05	8.64E-05	2.266486	down
LINC02241	1.002516	4.33146	4.478372	1.88E-05	8.96E-05	2.228646	up
RGS2	-1.20775	9.316481	-4.47366	1.92E-05	9.10E-05	2.210897	down
EHHADH	-1.02992	10.67619	-4.47339	1.92E-05	9.11E-05	2.209862	down
ACSM5	-1.05433	10.13005	-4.47109	1.94E-05	9.18E-05	2.20123	down
SLCO4C1	-1.10598	5.558204	-4.46642	1.97E-05	9.33E-05	2.183642	down

SLCO1B1	-1.0297	9.601288	-4.44429	2.15E-05	0.0001	2.100537	down
CTNNA2	1.168205	5.595887	4.417119	2.40E-05	0.00011	1.998874	up
FZD6	1.081531	6.207777	4.415352	2.41E-05	0.000111	1.992281	up
HAO1	-1.2247	10.9783	-4.41306	2.43E-05	0.000112	1.983709	down
F9	-1.53246	11.9819	-4.40858	2.48E-05	0.000114	1.967015	down
NQO1	1.440516	7.236253	4.395736	2.61E-05	0.000119	1.919175	up
SLC13A5	-1.3732	9.781289	-4.3893	2.67E-05	0.000121	1.895239	down
MROH2A	-1.06259	6.34598	-4.36477	2.94E-05	0.000132	1.804249	down
PFKFB3	-1.00413	8.515998	-4.36071	2.99E-05	0.000134	1.789196	down
DSEL	-1.03811	5.897354	-4.35716	3.03E-05	0.000136	1.776089	down
ASPN	-1.23927	8.475406	-4.35362	3.07E-05	0.000138	1.762997	down
LUM	-2.01648	8.086675	-4.34935	3.12E-05	0.00014	1.747206	down
ACMSD	-1.08395	9.922591	-4.34004	3.23E-05	0.000144	1.712863	down
UNC93A	-1.1686	7.650674	-4.31667	3.54E-05	0.000156	1.626834	down
PAGE4	1.440554	5.983869	4.315463	3.56E-05	0.000157	1.622383	up
FGFR2	-1.42193	8.680705	-4.30021	3.77E-05	0.000166	1.566412	down
DPYS	-1.2883	11.53264	-4.27701	4.12E-05	0.000179	1.481564	down
SLC10A1	-1.4885	10.88556	-4.27571	4.14E-05	0.00018	1.476836	down
FHL2	-1.00799	7.905797	-4.26975	4.24E-05	0.000183	1.455069	down
CLGN	1.309439	5.894618	4.265357	4.31E-05	0.000186	1.439071	up
A1BG	-1.11727	12.30704	-4.26149	4.38E-05	0.000188	1.424982	down
C12orf75	1.031639	7.018783	4.258002	4.43E-05	0.00019	1.412289	up
ZNF595	1.233284	4.960928	4.242714	4.70E-05	0.0002	1.356729	up
UGT2A3	-1.0805	7.122263	-4.24037	4.74E-05	0.000202	1.348218	down
TOX3	1.099463	8.010222	4.237617	4.79E-05	0.000204	1.338237	up
PPM1E	1.00299	5.149173	4.220608	5.11E-05	0.000215	1.27664	up
PEG10	2.032716	8.281787	4.193163	5.67E-05	0.000236	1.177622	up
EPHA3	-1.14243	5.632172	-4.18991	5.75E-05	0.000239	1.165924	down
LYZ	1.098493	11.16124	4.186465	5.82E-05	0.000242	1.153526	up
FETUB	-1.5224	8.781648	-4.16923	6.21E-05	0.000256	1.09164	down
GDA	-1.06593	9.437309	-4.16702	6.26E-05	0.000258	1.083749	down
UGT3A1	-1.19413	9.213434	-4.13979	6.94E-05	0.000282	0.986376	down
PKHD1	-1.01258	6.073946	-4.11588	7.59E-05	0.000305	0.901276	down
CXCL11	1.148336	5.146765	4.085584	8.50E-05	0.000338	0.793983	up
ADH1C	-1.47354	11.66511	-4.08154	8.63E-05	0.000343	0.779714	down
ACSM1	1.038928	6.52365	4.068762	9.05E-05	0.000357	0.734648	up
PDZK1IP1	1.380529	6.814877	4.068339	9.06E-05	0.000358	0.733159	up
OTC	-1.40772	9.586321	-4.05811	9.41E-05	0.00037	0.697172	down
LOX	1.000065	5.32776	4.047761	9.78E-05	0.000383	0.660824	up
ITGB8	-1.16807	4.621629	-4.04589	9.85E-05	0.000385	0.654266	down
MTTP	-1.05554	10.60704	-4.00143	0.000116	0.000446	0.498964	down
GTSF1	1.156745	6.449369	3.980301	0.000125	0.000478	0.425584	up
DIO1	-1.2984	10.53524	-3.95566	0.000137	0.000519	0.340384	down
NRCAM	1.186842	6.066239	3.952196	0.000139	0.000524	0.328445	up
PCK1	-1.47161	11.87107	-3.94629	0.000142	0.000534	0.30808	down
REG3A	1.997009	6.456436	3.945648	0.000142	0.000535	0.305885	up
CYP8B1	-1.49501	10.94229	-3.94067	0.000145	0.000543	0.288753	down
S100P	1.636449	7.022874	3.93638	0.000147	0.000551	0.274002	up

PTGDS	-1.06825	9.286912	-3.89122	0.000173	0.000638	0.119469	down
HPD	-1.7277	11.46115	-3.87208	0.000186	0.000678	0.054342	down
LOC10050!	-1.04725	6.95467	-3.85434	0.000198	0.000718	-0.00575	down
TNFRSF19	1.29223	6.006611	3.828166	0.000217	0.00078	-0.09409	up
SLC51A	-1.18222	9.444337	-3.82734	0.000218	0.000782	-0.09686	down
ADGRG2	1.001887	5.897341	3.807735	0.000234	0.000834	-0.16272	up
DKK1	1.704653	5.815915	3.744655	0.000292	0.001018	-0.37283	up
ALDH3A1	1.380246	6.425607	3.713451	0.000326	0.001121	-0.47578	up
CPS1	-1.26232	12.51349	-3.68718	0.000358	0.001215	-0.56194	down
FABP1	-1.30807	12.40299	-3.64012	0.000421	0.001401	-0.71507	down
SLC26A3	1.058492	4.309986	3.612481	0.000463	0.001525	-0.80431	up
CYP2E1	-1.27002	13.23124	-3.60381	0.000477	0.001564	-0.83219	down
SERPINA7	-1.12797	10.57074	-3.59843	0.000486	0.00159	-0.84949	down
LGSN	-1.11642	5.37638	-3.55067	0.000572	0.001836	-1.00194	down
NR1I3	-1.03878	8.904149	-3.51591	0.000643	0.002031	-1.11189	down
APCS	-1.03902	12.50434	-3.47717	0.000733	0.002278	-1.23344	down
MMP12	1.194382	5.158712	3.428331	0.000862	0.002631	-1.38513	up
DEFB1	-1.24695	10.36792	-3.40864	0.00092	0.00279	-1.4458	down
ADH1B	-1.12982	12.61992	-3.40049	0.000945	0.002858	-1.47083	down
RELN	-1.09249	9.252797	-3.37609	0.001025	0.003074	-1.54548	down
DCAF4L2	1.087983	4.046772	3.358104	0.001087	0.003233	-1.60023	up
SULT2A1	-1.06519	11.35716	-3.35419	0.001101	0.003269	-1.6121	down
AKR1C4	-1.01607	10.82466	-3.31951	0.001232	0.003612	-1.71691	down
C15orf48	1.004439	6.310673	3.266895	0.00146	0.004193	-1.87421	up
PON1	-1.09235	11.82488	-3.22694	0.001659	0.004696	-1.99229	down
RTP3	-1.05877	8.834906	-3.20313	0.00179	0.005014	-2.06208	down
ARG1	-1.0279	12.14971	-3.142	0.002169	0.005924	-2.23935	down
NPW	-1.04719	6.015032	-3.00802	0.003276	0.008529	-2.61782	down
HSD11B1	-1.0642	11.25	-2.94634	0.003944	0.010009	-2.7874	down
CRP	-1.48627	10.0836	-2.92831	0.004162	0.010474	-2.83642	down
PEG3	-1.05022	6.756366	-2.90489	0.004462	0.011119	-2.89969	down
