# Supplementary material for: Exploring the Multicomponent Synergy Mechanism of Yinzhihuang Granule in Inhibiting Inflammation-Cancer Transformation of Hepar Based on Integrated Bioinformatics and Network Pharmacology
Source: Biomed Res Int. 2022 Mar 18;2022:6213865. doi: 10.1155/2022/6213865 (PMC8956385; doi:10.1155/2022/6213865)
Supplement: Supplementary Materials — contain eight tables. Supplementary Table S1: the information of differentially expressed genes in GSE83148. Supplementary Table S2: the information of differentially expressed genes in GSE121248. Supplementary Table S3: the information of targets in the PPI network of hepatitis C. Supplementary Table S4: the information of differentially expressed genes in GSE17548. Supplementary Table S5: the information of 25 compounds in YZHG. Supplementary Table S6: relationship between network points of target nodes of YZHG. Supplementary Table S7: relationship between network points of target edges of YZHG. Supplementary Table S8: the information of 4-group disease data. Supplementary Table S9: the information of the drug-disease association network. Supplementary Table S10: the molecular docking result analysis. [file 6213865.f1.zip › Supplement Table S8 (1).pdf]

H-HBV	HBV-HBV、HCC	HCV（data-PPI）	HCV、LC-HCV、HCC
KNCN	CXCL14	B2M	GPR158
RBM42	ANGPTL6	BCL2	ASPM
HDAC6	VIPR1	CASP1	TTK
AKT1	IGFALS	CBL	CENPW
RHOB	CLEC1B	CXCL10	PKHD1
UBA1	HHIP	CYP24A1	LY6E
YARS2	CDHR2	CYP27B1	KIF18B
LINC00663	ECM1	CYP2R1	STEAP4
GLYATL1	KCNN2	EGF	NGFR
AKIRIN2	ADAMTS13	EGFR	IRF8
BANF1	OIT3	EREG	KNL1
PEX19	FCN2	FGF1	AVL9
ACTN4	CLEC4M	FGF10	UHMK1
TIE1	CLEC4G	FGF2	PTPRS
FKBP1A	PLVAP	FGFR1	NDC80
JUND	ZGPAT	FGFR2	CENPU
AP2M1	CAP2	GAB2	SHBG
SUZ12P1	STAB2	GRB2	SFRP5
CFL1	CRHBP	HLA-A	RBBP5
PLD3	LINC01093	HLA-B	BMP5
ILF3-DT	CYP26A1	HLA-C	CXCL14
PFN1	ACADS	HLA-E	RACGAP1
IGFBP4	ANKRD55	HLA-F	MET
SDHAF3	KDM8	HLA-G	TOP2A
CST3	PAMR1	HRAS	AKT1S1
C1RL-AS1	COLEC10	IDO1	CENPF
LRRC25	TOP2A	IFIT1	CDKN2C
SDC3	PTH1R	IFNA2	CD300A
CIDEB	NXF3	IFNA5	CCNA2
ZNF224	FCN3	IFNA8	ANGPTL6
GPX3	RIPOR3	IFNAR1	STOM
ADAMTSL4	DBH	IFNAR2	KIF2C
NUDT16	LCAT	IFNL3	BAIAP2L1
LENG8	CNDP1	IFNW1	CCNE2
WBP2	RACGAP1	IL18	CERS2
TAPBP	ASPM	IL6	KCNJ16
PLTP	CCBE1	IL6R	NECAB3
CPLANE2	RSPO3	ISG15	FAM83D
CD74	BMPER	ITPA	PZP
LOC100129[illegible]	ADGRG7	JAK2	PLOD3
SGMS1	IDO2	KRAS	SUV39H1
SH3BGRL3	COL15A1	LDLR	RCAN1
UBXN2A	PLAC8	MLLT4	NUF2
TNFAIP2	CFP	MMP1	CYGB

PPP4R4	CSRNP1	MMP13	RAD54B
PARVG	PSMD4	MMP2	CENPE
FXYD5	HMMR	MMP9	KIF14
LOC1002874	PITPNM3	MPL	JDP2
IL32	TBXA2R	MX1	C1QTNF1
NCAM2	SLITRK6	NTRK1	DLGAP5
CCDC125	CLTRN	OASL	CEBPG
RTN1	TTC36	PCSK9	RMI1
METTL7B	MSH2	PDGFRB	PPP2R5A
PLEKHA6	MAP2K1	PIK3CA	CX3CL1
SLC22A3	CDC37L1	PIK3CG	BRIP1
DCAF17	FAHD2A	PLCG1	PCLAF
ZNF160	NUSAP1	RAF1	CHST4
GRTP1	TCTEX1D1	RALGDS	PHF14
GEN1	EPHA2	SCARB1	PPP1R35
PTGR2	PZP	SHC1	CST7
ZNF721	CDKN3	SOS 1	PRKCA
KANSL1L	EZH2	SPRY2	CCN1
PARVB	PRC1	SSTR4	MKRN1
MINDY1	ECT2	STAT3	EFEMP2
THAP5	CYP1A2	SYK	NCAPG
MMD	HAND2-AS1	TGFA	KIF20A
BTG2	LIFR	TIMP2	SATB2
C8orf44	ANLN	TLR7	EIF2AK1
HCLS1	CENPW	TLR9	DHX35
ARRDC3	GPR146	TYK2	CFTR
SULT1E1	MASP1	VDR	MYO10
NETO2	SHBG	ZAP70	FXYD6
TXNIP	CETP		SPNS2
HCK	SRPX		MANEAL
PDIA3	SLC25A47		CD8A
PGF	OLFML3		SPOCK1
ZNF814	ITGA6		KCTD6
CTSLP8	HGFAC		MIS18A
RCSD1	UROC1		MCM8
LCP2	TBC1D16		ADAMTSL2
P2RY13	NEK2		NKD2
FCER1G	LYVE1		CTNNA2
TGFB1	DTL		CCDC3
GPR37	MSRA		IL2RB
PRG4	RRM2		DBH
HAUS2	HGF		NEK2
HPR	GGTLC1		HGFAC
FGF14-AS2	NGFR		CDK1
MMP2	ENO3		TNFRSF1B

ABO	GJC1	FEZ1
SLITRK3	IGFBP3	PDE4A
ITPR2	KAZN	CDK5
SASH3	RCAN1	NR2F6
SLCO4C1	RND3	IPP
ZBTB10	NDC80	TWF1
BARD1	NAT2	ECM1
MAB21L2	ASS1	NUSAP1
PTPRE	MRO	PLPP4
SLC25A47	BUB1B	PLEKHA2
PDE4B	IRAK1	HJURP
TRHDE	TPPP2	LILRB2
S100A11	ZBED8	FANCI
TPX2	FANCC	PTGS2
DEPDC1	CPEB3	MELK
PVRIG	CKAP2	C17orf58
PLP2	HAMP	CAP2
LOC102724	ZG16	E2F8
ADA2	STMN1	TBXA2R
SLC25A43	PLPP3	JAML
OSBPL3	TDRKH	SPAG7
LAIR2	RFX5	IFITM1
MAML2	SSR2	BUB1B
KLF4	SRD5A2	ZKSCAN8
CD44	FLVCR1	VPS54
SUSD3	NOCT	CDC20
MAP2K6	UBE2T	CCNB2
CCND2	BDH2	DNAH14
NR1D2	IGHM	IL4R
CAPN2	FAM13A	STAP1
HLA-DOA	JDP2	CENPL
AFMID	CD5L	ATAD2
ESF1	CCNB1	DUXAP10
TYMS	AADAT	MND1
FCRL3	CXCL12	LOC101926944
KCNJ3	GSTZ1	ZNF831
CNKSR2	SIGIRR	PRF1
ZFPM2	ESM1	QSOX1
ITK	CLRN3	MATK
NOL4	KANK4	EOMES
BCHE	CDK1	ARIH2
OAS2	FOXO1	DDX41
TM6SF1	MARCO	COPA
CCNA2	TRIB1	CDCA5
CXCL9	CCDC34	IGLJ3

CD2	SLC28A1	SPC25
JAZF1	PODXL	JADE3
SPDYE2	ILF2	CCL4
IGLC1	SLC9B2	C11orf96
MTHFD2	FEZ1	KIF4A
EGR2	GINS1	C1orf162
CDKN1A	DNMT3L	CCL5
MOGAT1	RAD51AP1	ECT2
CCNE2	PBK	RRM2
SSPN	PDE7B	NDRG2
SAMD9L	CENPF	ADGRB3
CXCL10	NPY1R	SRPX
FUS	MELK	SULF2
STK39	LRRN3	UBE2C
ENPP2	FRMD4B	PTPRN2
ZNF738	CCNB2	CNTN4
TTPA	UHRF1	PLCB1
MEIOC	GPD1	CEP55
DTL	PHGDH	TPBG
PHLDA1	CCT3	RASD1
HLA-DMA	PALM3	SGO2
CD8A	CENPU	CLTRN
LOC101926[illegible]	PTTG1	IGLL3P
JAKMIP2	STEAP3	GPSM2
TOX	SPRYD4	NR4A3
ADAMTS17	SYNE1	SERPINB9
BCL2A1	SLC19A3	PODN
RGS1	GDPD1	ILDR2
LCK	KIF20A	ALKAL2
IGKC	FAM83D	KIF11
CD69	CDH19	EZH2
IGLL3P	LY6E	SOWAHA
TRIM22	BCO2	LOC730101
NPAS2	NCAPG	TACSTD2
CPEB3	PCDH9	CH25H
PTPN22	BBOX1	PWWP3B
GPR18	BIRC5	COLEC10
PNMA2	DNASE1L3	COL4A3
LONRF2	CHST4	OIP5
P4HA1	SFRP1	AKAP7
LINC01554	GPC3	TRIP13
GNL3L	SGO2	TMEM209
CD27	MAD2L1	GGT5
GSN	DIRAS3	SKA1
GPNMB	DNAJC6	FOXM1

NEK2	RFC4	MZB1
PTTG1	ESR1	IGFBP3
APCDD1	KPNA2	LCP2
RRM2	C3orf85	ADAMTS13
SERPINE2	KMO	COX7B2
SLC7A1	TCF21	EPB41L4A
GPR88	KIF4A	MAGEA6
ABCB11	FOSB	SSX2IP
TRAC	TAF1A	THEMIS2
TRBC1	COL4A1	PRC1
MYOM1	ASPA	KIF15
COL4A4	RAB26	IGFBP6
CNTN3	CDKN2C	BTG2
TSPAN13	LPA	PLAC8
NUSAP1	PLIN1	CCBE1
CSF2RB	ALDH1B1	PRKAR2B
COL6A2	ZWINT	CDH19
SOX4	TCIM	LTBP4
CACNA2D1	SPRY2	CDHR2
PLXNC1	MOGAT2	DPT
SPATA18	STIL	CYS1
SLFN12	PCLAF	ROBO1
HINT3	FOLH1B	LCAT
TRAF5	DUXAP10	COLEC11
KCNK5	TRIP13	AKR1C3
EFEMP1	APOF	GINS1
LEPR	C1orf112	RASSF5
LOC100190[illegible]	JCAD	DEPDC1
MROH2A	ROBO1	INMT
LINC01410	CENPK	TRIM22
CCL4	GPR158	SH3YL1
TMEM154	PROZ	PLCXD3
CDK1	ACSM3	GZMK
GALK1	MCM3	SLAMF7
GZMA	KBTBD11	EPHA2
ASB9	ENAH	DTNA
GOLM1	RCL1	CLIC6
RGS4	TBC1D31	ZWINT
TFEC	MFSD2A	JCHAIN
RASGRP1	CENPL	TMC8
STK17B	LPAL2	EML6
HPSE	FOS	USP31
COL4A3	HOTS	GUSBP11
PAX8-AS1	CNIH4	RIPOR3
LOC100507[illegible]	TM6SF2	RAD51AP1

CCR2	TBX15	CENPK
LAMP3	TKFC	SEMA4D
CCL21	RNF165	IER3
CCL8	ID1	PBK
MAD2L1	TUBE1	ZBTB41
CCL5	RAP2A	DUSP5
GZMK	SARDH	IGHD
CXCL8	TCF19	PHLDA1
ZWINT	NPC1L1	MAD2L1
KBTBD11	SLC39A5	CLDN10
CHST4	FCGR2B	CRHBP
POU2AF1	TTK	UBE2T
SOX9	ADRA1A	CCNB1
ZMAT3	RGS5	VTCN1
PCLAF	ITGA9	SMIM3
DKK3	INMT	ANTXR2
PTGDS	HOGA1	OLFM1
FABP5	HELLS	APOA5
ADAMDEC1	STXBP6	DCAF4L2
TOP2A	SUCLG2	CCL2
EHF	NAPSB	ONECUT2
CCL19	NUF2	LPA
HKDC1	CCNA2	TFPI2
KCNN2	ADIRF	CDKN1C
NIBAN1	DPT	HMMR
PLEK2	PBLD	DCN
SLAMF7	PLCB1	RSPO3
RCAN2	ACSL4	IGK
LPA	GCH1	EGR2
COL5A1	IGF1	TPSAB1
CLDN10	C7	FCN3
ND6	EBF1	GABRP
CUX2	E2F8	NR4A1
RGCC	TTPAL	CHRM3
MGP	ANK3	SPINT2
PDGFD	PRIM1	TPX2
SMOC2	JCHAIN	LDOC1
UHRF1	CDC20	CXCL12
CXCL11	AKR7A3	LCK
	KLKB1	CSGALNACT1
	SULT1E1	POU2AF1
	PNP	IGLV1-44
	NAAA	GNA14
	MAN1C1	GMFG
	MND1	KLF2

MCM6
CRNDE
UBE2S
FANCD2
ST6GAL2
CYP39A1
CYP2C19
CPED1
IRF8
GCDH
ZFP36
CDCA3
THY1
EDNRB
TIGD1
ADAMTSL2
SLC35D1
MRC1
SULF2
FXYD1
HAO2
DLGAP5
PLSCR4
SMIM24
TP53I3
CXCL2
CENPH
FAM151A
CD200
EPB41L4A
ST3GAL6
IER2
RBMS3
AURKA
LHX2
CIDEB
CYP2B6
RBM24
TOMM40L
ZFP1
STEAP4
CDC7
MTFR2
ACACB
MAMDC4
LIF
ENAH
PTPN22
C1orf112
C7
ST8SIA6-AS1
HGF
CD163
ITGB8
HELLS
CYP1A2
CFP
AURKA
CDKN3
OLFML3
KAZN
CRISPLD2
NPY1R
CCL21
DNM3OS
RBM24
CDKN2B
F3
PDGFRA
FGFR2
TMEM154
ADAMTS1
BASP1
RND3
SLC25A47
CRTAM
MASP1
SMPX
TAGAP
PROM1
MAP2
DNASE1L3
SPATA18
FAM169A
RFC3
CLEC1B
KLF4
MFAP4
DSE
HCLS1

SOCS2
OTUD6B
CDC25C
ETFDH
LINC00598
RETREG1
LDHD
AGL
GMNN
CCNE2
FOXM1
MS4A6A
HSPB1
MT1G
LDLR
C1RL
GLS2
CENPE
KIF11
ANXA2
SLC38A6
IL13RA2
EGR1
ACAA1
FBP1
CYFIP2
OLFML2B
MT1F
PPID
FREM2
SLC3A1
MAGI2-AS3
TEK
PLCXD3
EML6
EPHX2
KCNMA1
DCN
DEPDC1B
PGLYRP2
CYP2A7
EGR2
GHR
POLE2
SLC16A4
IGLC1
EDNRB
LIFR
GREM2
SYT1
CLEC4G
SVEP1
PLIN2
FCN2
TENT5C
LOC105373150
TPSB2
MXRA5
EGR3
RCAN2
ADGRE5
PRICKLE1
ZFP36
EMILIN1
IL10RA
FGGY
BGN
IGKC
ATF5
PCDH9
GPR65
ZSCAN18
LINC01093
TUBB6
IGHM
BMS1P20
PRSS8
MEIS3P1
WNK3
PTPRC
RGS4
STAB2
FOS
GSTZ1
TRBC1
RBMS3
FOXF1
SSX1
CPA3
RGS2

KIF18B
OIP5
LOC389834
PHYHD1
CCDC3
UBE2C
PRSS8
NR1I2
RBP7
GBA3
PANK1
SLC17A1
GABARAPL1
TACSTD2
ZIC2
PDGFRA
SLC41A2
SMYD3
CYP4V2
HMGB2
TENM1
SERPINI1
MT1H
GCKR
CA2
PPP1R3B
GREM2
DUSP6
NR0B2
CDC6
NR3C2
IGKC
ATF5
SKAP1
AKR1C3
KIF2C
GPR180
CDKN2B
ANXA10
LAMC1
GLDC
SQLE
ATF3
FTCD
PROM1
CYTIP
TNFRSF17
CCL19
RAC2
SHCBP1
HSPB6
TRIM71
TXNRD1
KBTBD11
MAGEA12
GAS1
MPEG1
PLA2G5
JUNB
CD1D
LAMA2
ITK
CD2
ANXA3
MAMDC2
SRGN
S100A8
ZFPM2
DTL
GPM6A
LHX2
MAGEA1
HAS2
THBD
SP5
THBS1
GUCY1A1
APOF
ARL4C
AMIGO2
ID4
ITGA9
PGLYRP2
EVI2B
CETP
COL4A4
CYP17A1
IFI44
PTN
KRT19

GADD45B
NR4A3
GLYAT
MT1M
ST8SIA6-AS1
ECRG4
PLIN2
KNL1
FAM149A
DLG5
TMEM45A
SYTL5
APOBEC3B
C3P1
CD1D
IGLC1
AGXT2
PSAT1
C1orf162
CYP4A11
EDIL3
MNS1
SLC17A3
MT1HL1
SLCO1B3
IL1RAP
IGLL3P
EPB41L4B
SERPINE1
SPC25
CEP55
OSBPL3
SPINK1
TRPM8
FBLN5
SULT1A2
RNF125
PIK3C2G
MT1E
CNTN4
ANGPTL1
AQP3
CHML
SERPINB9
SLC7A2
SDC3
CD53
ADA2
FCER1A
MAGEC2
CYBA
HBB
PFKFB3
ID1
TRAC
GZMA
PDGFD
TCIM
MNS1
WFDC1
FGL2
PAGE4
CD69
TMEM45A
DEPDC1B
PDE4B
TMEM100
ENG
EGR1
PTGIS
MFSD2A
AXL
OIT3
PTPN13
KLRB1
GPR171
RHOH
VSIG4
CLEC2B
NFASC
HAND2-AS1
MARCO
ISLR
DKK1
NALCN
MT1M
PLAAT4
COL24A1
CMAHP
KDM8

CA5A
CTHRC1
DHRS1
EXOC3L4
COMT
CDKN1C
FGL2
THRSP
PPBP
FAS
GAS2L3
OGDHL
RDH16
COL4A2
SLC22A1
CCN1
OAT
MT1X
AGBL3
KCND3
TFPI2
SERPINA4
NNMT
SIPA1L2
TREH
SHCBP1
KCNJ16
LRRC1
CLYBL
N4BP2L1
IGF2BP3
CYP2A6
MCC
ZFPM2
MT2A
ATAD2
MYO10
GADD45A
AKR1D1
CYP2C18
DEPDC1
C1R
DEPDC7
PRODH2
FABP5
APOBEC3B
IGF2BP3
C1QA
SLCO1B3
GNG2
ENO3
TCF21
IL6
NMRAL2P
CALHM6
RASGRP1
STK39
CD48
ITGB2
AQP1
CRNDE
LAMA3
CLDN11
CORO1A
IRX3
FILIP1L
NAT2
AFM
EPO
WIPF1
LINC02241
ANK3
SLC7A11
FBLN5
GHR
ZNF331
CD52
CKS2
ZIC2
EPCAM
FHL2
OTUD6B
PCOLCE2
MRC1
C16orf54
CNKSR2
PRKAA2
THRSP
EPHA3
PLA2G4A

TXNRD1
TPX2
AKR1B10
CNTN3
GSPT2
PTGS2
SOX6
TKT
MCM4
WDR72
SERPINF2
PTGIS
CLDN10
LIPC
ACADL
PHLDA1
PYROXD2
F11
CD109
PRG4
HBB
C8A
CYP3A43
IYD
BCHE
GBP1
CYP2J2
CYP2C9
CCL2
CALHM6
NAMPT
APOA5
RBP5
FOXP2
GYS2
TRIM16
GRAMD1C
MIR99AHG
BAG2
LINC01554
LOC101928505
LEF1
VNN1
ABI3BP
AFM
IL7R
CYP7A1
ASPN
CNDP1
SCML4
ACSM1
METRNL
ALOX5
HLA-DPB1
TTC36
LDHB
KMO
RGCC
CCDC150
TAGLN
ALOX5AP
PLAGL1
S100A4
NR4A2
ARHGAP30
CLGN
F5
FXYD2
RBP1
ENDOD1
SOCS3
SULT1C2
KCNU1
PIK3CG
RAB25
DNAJC6
COL6A2
CCND2
DOCK8
FZD1
EVI2A
LOXL4
ANGPTL1
COL14A1
ADGRG7
LXN
TSPYL5
MTHFD2
CLEC4M
PDE1A

BASP1
DAO
FYB2
HABP2
GPT2
SLC46A3
COLEC11
TSPAN12
PRKAA2
CCDC198
CYP3A4
NMRAL2P
ACSL1
ID4
CFTR
LINC01146
CPN2
SLC22A4
SDS
GABRP
SFN
SLC25A18
CDA
SULT1C2
PDE11A
SSX1
CCL4
DUSP5
IL33
PRKAR2B
TDO2
CCL5
MBL2
CRISPLD2
SEMA6D
VSIG4
KYNU
SAMD5
SLC17A2
ADAMTSL3
BGN
THBS1
TMEM154
C9
ALDH8A1
CYBRD1
CXCL6
CRIP1
CRP
KRT7
RNASE6
LUM
MYC
CELF2
ANTXR1
C9
TMEM64
DACT1
PLAT
AEBP1
PMP22
CXCL1
MT1F
CSF2RB
IL33
MS4A4A
TIMP2
LBH
TYROBP
MT1X
ACSM3
CXCL8
CD44
SCN7A
GABRB3
HHIP
SPINK1
INHBE
C1QC
SSPN
FOSB
PAGE2B
CRACD
CPS1-IT1
CYP2C8
PTGDS
MT1G
C1QB
COTL1
LIN28B

ADAM9
SPIDR
CCL21
FXYD2
PLAG1
MAP2
ARRDC4
HRCT1
NR4A2
ADAMTS1
PLGLB2
CYP2B7P
GJB2
FAM110C
SLC25A15
SOCS3
ADH6
CTH
HYAL1
CYP4F2
KLF4
DAB1
MXRA5
DNAJC12
SPP2
SORL1
XDH
THBS4
CD163
CCL19
DTNA
TAT
ART4
PCK2
AVPR1A
C11orf96
C6
LINC00844
ACADSB
ABCA8
SRD5A1
CD69
SLC6A12
LECT2
CFHR3
S100A6
LINC01419
AFAP1-AS1
ALPK2
CD37
CA2
FREM2
PAPLN
RGS1
GEM
MT1E
FSTL5
MS4A1
ANK2
BICC1
FAM149A
MOXD1
CCN2
HAMP
RUNX2
ID3
VCAM1
MYL9
FAM13A
ARMCX3
ADAMTS2
HLA-DRB4
FMO2
CXCR4
ZEB2
SEL1L3
DSEL
CNTN3
RCSD1
MMP7
BCO2
NTS
LHFPL6
WDR72
TNFAIP8
PDZRN3
MS4A7
EHF
NPW
C1QTNF7

UGT2B28
ISX
TSLP
ACOT12
ANG
POU2AF1
GIPC2
SPP1
PLXNC1
ANO1
CCL20
ALKAL2
ANKRD29
FGF13
STK39
EVA1A
LINC01419
ZSCAN31
PLA1A
PTPRD
ADH4
SLC27A5
LAMA3
LCN2
GAS1
LOC157273
GNAL
ARMCX3
COX7B2
CP
GNMT
SLC4A4
C8B
SMPX
PPARGC1A
SLC16A14
EGR3
HAL
SH3YL1
B3GNT5
RMST
MAGEA12
PPP1R1A
CYTIP
FAM133A
ESR1
BEX4
SAMSN1
LOXL1
ITGBL1
AFP
IGF1
HLA-DRA
ABI3BP
TRIM16
ANXA1
NNMT
SQLE
FBP1
MMRN1
MGP
ALDH8A1
GPR88
ADH1C
SLC3A1
EFEMP1
SOCS2
COL3A1
CLSTN2
PTGER4
PTH2R
VIM
GLS2
RETREG1
EDIL3
DKK3
GBA3
TENM1
PCK1
HPGD
AKR1B10
SLC22A1
REG3A
NQO1
PEG10
APCDD1
TDO2
GPC3
GSTT1
PLA2G2A

BHMT	XIST
EP300-AS1	
TRIM22	
GREB1	
REEP6	
GEM	
HSD17B2	
CDH1	
MFAP3L	
MLIP	
XK	
FNDC5	
S100A8	
MAGEA1	
MYH4	
TRIM71	
MAGEA6	
COL8A1	
LINC01831	
ETNPPL	
COL4A3	
PCDHB14	
INHBE	
SLC22A7	
SPARCL1	
CFHR4	
ITIH4	
FAM169A	
PWWP3B	
ARHGEF26	
CYP2C8	
COL14A1	
PDZRN3	
PIGR	
DMGDH	
ADH1A	
SLC7A11	
CXCL6	
HPGD	
SLC27A2	
LINC02241	
RGS2	
EHHADH	
ACSM5	
SLCO4C1	

SLCO1B1
CTNNA2
FZD6
HAO1
F9
NQO1
SLC13A5
MROH2A
PFKFB3
DSEL
ASPN
LUM
ACMSD
UNC93A
PAGE4
FGFR2
DPYS
SLC10A1
FHL2
CLGN
A1BG
C12orf75
ZNF595
UGT2A3
TOX3
PPM1E
PEG10
EPHA3
LYZ
FETUB
GDA
UGT3A1
PKHD1
CXCL11
ADH1C
ACSM1
PDZK1IP1
OTC
LOX
ITGB8
MTTP
GTSF1
DIO1
NRCAM
PCK1

REG3A
CYP8B1
S100P
PTGDS
HPD
LOC100505985
TNFRSF19
SLC51A
ADGRG2
DKK1
ALDH3A1
CPS1
FABP1
SLC26A3
CYP2E1
SERPINA7
LGSN
NR1I3
APCS
MMP12
DEFB1
ADH1B
RELN
DCAF4L2
SULT2A1
AKR1C4
C15orf48
PON1
RTP3
ARG1
NPW
HSD11B1
CRP
PEG3
