# Supplementary material for: Exploring the Multicomponent Synergy Mechanism of Yinzhihuang Granule in Inhibiting Inflammation-Cancer Transformation of Hepar Based on Integrated Bioinformatics and Network Pharmacology
Source: Biomed Res Int. 2022 Mar 18;2022:6213865. doi: 10.1155/2022/6213865 (PMC8956385; doi:10.1155/2022/6213865)
Supplement: Supplementary Materials — contain eight tables. Supplementary Table S1: the information of differentially expressed genes in GSE83148. Supplementary Table S2: the information of differentially expressed genes in GSE121248. Supplementary Table S3: the information of targets in the PPI network of hepatitis C. Supplementary Table S4: the information of differentially expressed genes in GSE17548. Supplementary Table S5: the information of 25 compounds in YZHG. Supplementary Table S6: relationship between network points of target nodes of YZHG. Supplementary Table S7: relationship between network points of target edges of YZHG. Supplementary Table S8: the information of 4-group disease data. Supplementary Table S9: the information of the drug-disease association network. Supplementary Table S10: the molecular docking result analysis. [file 6213865.f1.zip › Supplement Table S9.pdf]

H-HBV	HBV-HBV、HCC	HCV（data-PPI）	HCV、LC-HCV、HCC
MMP2	**XDH**	**MMP2**	**PTPRS**
GALK1	SLCO1B3	MMP13	SLCO1B3
CDK1	AKR1B10	TLR9	ALOX5
BCHE	CYP2C19	CASP1	CYP1A2
TOP2A	CDK1	STAT3	AKR1B10
LCK	BCHE	EGFR	PLA2G2A
TYMS	ADRA1A	MMP1	CCNB2
	AKR1C4	MMP9	CDK1
	PTGS2		TOP2A
	CYP2C9		CA2
	CA5A		HBB
	ESR1		LCK
	SLCO1B1		CDK5
	HPGD		PTGS2
	EPHX2		CHRM3
	MMP12		ESR1
	CYP1A2		AKR1C3
	NPC1L1		PMP22
	CCNB2		HPGD
	TOP2A		
	CA2		
	HBB		
	GMNN		
	AKR1C3		
	CYP3A4		
	HYAL1		
