# Supplementary material for: Exploring the Multicomponent Synergy Mechanism of Yinzhihuang Granule in Inhibiting Inflammation-Cancer Transformation of Hepar Based on Integrated Bioinformatics and Network Pharmacology
Source: Biomed Res Int. 2022 Mar 18;2022:6213865. doi: 10.1155/2022/6213865 (PMC8956385; doi:10.1155/2022/6213865)
Supplement: Supplementary Materials — contain eight tables. Supplementary Table S1: the information of differentially expressed genes in GSE83148. Supplementary Table S2: the information of differentially expressed genes in GSE121248. Supplementary Table S3: the information of targets in the PPI network of hepatitis C. Supplementary Table S4: the information of differentially expressed genes in GSE17548. Supplementary Table S5: the information of 25 compounds in YZHG. Supplementary Table S6: relationship between network points of target nodes of YZHG. Supplementary Table S7: relationship between network points of target edges of YZHG. Supplementary Table S8: the information of 4-group disease data. Supplementary Table S9: the information of the drug-disease association network. Supplementary Table S10: the molecular docking result analysis. [file 6213865.f1.zip › Supplementary table S7.pdf]

靶点（整理） default edge

Column 3	EdgeBetweenness	interaction	name	selected	shared interaction	shared name	化学成分
**Carboxy-terminal domain RNA polymerase II polypeptide A small phosphatase 1**	4.0	interacts with	shanzhiside (interacts with) CTDSP1	FALSE	interacts with	shanzhiside (interacts with) CTDSP1	山栀苷
**Maltase-glucoamylase**	183.59759528	interacts with	decetylasperulosidic acid methyl ester (interacts with) MGAM	FALSE	interacts with	decetylasperulosidic acid methyl ester (interacts with) MGAM	去乙酰车叶草苷酸甲酯
**Inosine-5'-monophosphate dehydrogenase 1**	554.0	interacts with	decetylasperulosidic acid methyl ester (interacts with) IMPDH1	FALSE	interacts with	decetylasperulosidic acid methyl ester (interacts with) IMPDH1	
**Inosine-5'-monophosphate dehydrogenase 2**	222.77368284	interacts with	decetylasperulosidic acid methyl ester (interacts with) IMPDH2	FALSE	interacts with	decetylasperulosidic acid methyl ester (interacts with) IMPDH2	
**Carbonic anhydrase II**	327.07124795	interacts with	decetylasperulosidic acid methyl ester (interacts with) CA2	FALSE	interacts with	decetylasperulosidic acid methyl ester (interacts with) CA2	
**Aldose reductase**	828.21142691	interacts with	decetylasperulosidic acid methyl ester (interacts with) AKR1B1	FALSE	interacts with	decetylasperulosidic acid methyl ester (interacts with) AKR1B1	
**Aldose reductase**	726.09207605	interacts with	neochlorogenic acid（5-caffeoylquinic acid） (interacts with) AKR1B1	FALSE	interacts with	neochlorogenic acid（5-caffeoylquinic acid） (interacts with) AKR1B1	新绿原酸（5-咖啡酰奎宁酸）
**Cyclooxygenase-2**	121.10251791	interacts with	neochlorogenic acid（5-caffeoylquinic acid） (interacts with) PTGS2	FALSE	interacts with	neochlorogenic acid（5-caffeoylquinic acid） (interacts with) PTGS2	
**LDL-associated phospholipase A2**	128.14216277	interacts with	neochlorogenic acid（5-caffeoylquinic acid） (interacts with) PLA2G7	FALSE	interacts with	neochlorogenic acid（5-caffeoylquinic acid） (interacts with) PLA2G7	
**Signal transducer and activator of transcription 1-alpha/beta**	277.06666667	interacts with	neochlorogenic acid（5-caffeoylquinic acid） (interacts with) STAT1	FALSE	interacts with	neochlorogenic acid（5-caffeoylquinic acid） (interacts with) STAT1	
**Signal transducer and activator of transcription 3**	277.06666667	interacts with	neochlorogenic acid（5-caffeoylquinic acid） (interacts with) STAT3	FALSE	interacts with	neochlorogenic acid（5-caffeoylquinic acid） (interacts with) STAT3	
**Tyrosine-protein kinase LCK**	136.19191645	interacts with	neochlorogenic acid（5-caffeoylquinic acid） (interacts with) LCK	FALSE	interacts with	neochlorogenic acid（5-caffeoylquinic acid） (interacts with) LCK	
**VEGF-receptor 2 and tyrosine-protein kinase SRC**	277.06666667	interacts with	neochlorogenic acid（5-caffeoylquinic acid） (interacts with) SRC	FALSE	interacts with	neochlorogenic acid（5-caffeoylquinic acid） (interacts with) SRC	
**Aldo-keto reductase family 1 member B10**	112.24890939	interacts with	neochlorogenic acid（5-caffeoylquinic acid） (interacts with) AKR1B10	FALSE	interacts with	neochlorogenic acid（5-caffeoylquinic acid） (interacts with) AKR1B10	
**Geminin**	261.09773824	interacts with	neochlorogenic acid（5-caffeoylquinic acid） (interacts with) GMNN	FALSE	interacts with	neochlorogenic acid（5-caffeoylquinic acid） (interacts with) GMNN	
**Glutaminase kidney isoform, mitochondrial**	173.6402792	interacts with	neochlorogenic acid（5-caffeoylquinic acid） (interacts with) GLS	FALSE	interacts with	neochlorogenic acid（5-caffeoylquinic acid） (interacts with) GLS	
**Histone-lysine N-methyltransferase, H3 lysine-9 specific 3**	121.10251791	interacts with	neochlorogenic acid（5-caffeoylquinic acid） (interacts with) EHMT2	FALSE	interacts with	neochlorogenic acid（5-caffeoylquinic acid） (interacts with) EHMT2	
**Lysine-specific demethylase 4A**	270.03222797	interacts with	neochlorogenic acid（5-caffeoylquinic acid） (interacts with) KDM4A	FALSE	interacts with	neochlorogenic acid（5-caffeoylquinic acid） (interacts with) KDM4A	
**Signal transducer and activator of transcription 5B**	277.06666667	interacts with	neochlorogenic acid（5-caffeoylquinic acid） (interacts with) STAT5B	FALSE	interacts with	neochlorogenic acid（5-caffeoylquinic acid） (interacts with) STAT5B	
**UDP-glucuronosyltransferase 1-6**	225.31845462	interacts with	neochlorogenic acid（5-caffeoylquinic acid） (interacts with) UGT1A6	FALSE	interacts with	neochlorogenic acid（5-caffeoylquinic acid） (interacts with) UGT1A6	
**UDP-glucuronosyltransferase 1-9**	225.31845462	interacts with	neochlorogenic acid（5-caffeoylquinic acid） (interacts with) UGT1A9	FALSE	interacts with	neochlorogenic acid（5-caffeoylquinic acid） (interacts with) UGT1A9	
**Carbonic anhydrase VB**	102.36544163	interacts with	neochlorogenic acid（5-caffeoylquinic acid） (interacts with) CA5B	FALSE	interacts with	neochlorogenic acid（5-caffeoylquinic acid） (interacts with) CA5B	
**Carbonic anhydrase V**	176.20431054	interacts with	neochlorogenic acid（5-caffeoylquinic acid） (interacts with) CA5A	FALSE	interacts with	neochlorogenic acid（5-caffeoylquinic acid） (interacts with) CA5A	
**Carbonic anhydrase VI**	191.05089754	interacts with	neochlorogenic acid（5-caffeoylquinic acid） (interacts with) CA6	FALSE	interacts with	neochlorogenic acid（5-caffeoylquinic acid） (interacts with) CA6	
**Carbonic anhydrase VII**	119.20910802	interacts with	neochlorogenic acid（5-caffeoylquinic acid） (interacts with) CA7	FALSE	interacts with	neochlorogenic acid（5-caffeoylquinic acid） (interacts with) CA7	
**Matrix metalloproteinase 12**	126.54291776	interacts with	neochlorogenic acid（5-caffeoylquinic acid） (interacts with) MMP12	FALSE	interacts with	neochlorogenic acid（5-caffeoylquinic acid） (interacts with) MMP12	
**Carbonic anhydrase XIV**	135.35148678	interacts with	neochlorogenic acid（5-caffeoylquinic acid） (interacts with) CA14	FALSE	interacts with	neochlorogenic acid（5-caffeoylquinic acid） (interacts with) CA14	
**Aldose reductase**	726.09207605	interacts with	neochlorogenic acid（5-caffeoylquinic acid） (interacts with) AKR1B1	FALSE	interacts with	neochlorogenic acid（5-caffeoylquinic acid） (interacts with) AKR1B1	
**Carbonic anhydrase XII**	211.39363213	interacts with	neochlorogenic acid（5-caffeoylquinic acid） (interacts with) CA12	FALSE	interacts with	neochlorogenic acid（5-caffeoylquinic acid） (interacts with) CA12	
**Matrix metalloproteinase 9**	111.09936271	interacts with	neochlorogenic acid（5-caffeoylquinic acid） (interacts with) MMP9	FALSE	interacts with	neochlorogenic acid（5-caffeoylquinic acid） (interacts with) MMP9	
**Protein-tyrosine phosphatase 1B**	102.36544163	interacts with	neochlorogenic acid（5-caffeoylquinic acid） (interacts with) PTPN1	FALSE	interacts with	neochlorogenic acid（5-caffeoylquinic acid） (interacts with) PTPN1	
**Matrix metalloproteinase-2**	125.23409163	interacts with	neochlorogenic acid（5-caffeoylquinic acid） (interacts with) MMP2	FALSE	interacts with	neochlorogenic acid（5-caffeoylquinic acid） (interacts with) MMP2	
**Carbonic anhydrases; II & IX**	140.48384046	interacts with	neochlorogenic acid（5-caffeoylquinic acid） (interacts with) CA9	FALSE	interacts with	neochlorogenic acid（5-caffeoylquinic acid） (interacts with) CA9	
**Collagenase**	126.54291776	interacts with	neochlorogenic acid（5-caffeoylquinic acid） (interacts with) MMP13	FALSE	interacts with	neochlorogenic acid（5-caffeoylquinic acid） (interacts with) MMP13	
**Carbonic anhydrase I**	135.35148678	interacts with	neochlorogenic acid（5-caffeoylquinic acid） (interacts with) CA1	FALSE	interacts with	neochlorogenic acid（5-caffeoylquinic acid） (interacts with) CA1	
**Matrix metalloproteinase (1 and 13)**	111.09936271	interacts with	neochlorogenic acid（5-caffeoylquinic acid） (interacts with) MMP1	FALSE	interacts with	neochlorogenic acid（5-caffeoylquinic acid） (interacts with) MMP1	
**Carbonic anhydrase II**	155.3976224	interacts with	neochlorogenic acid（5-caffeoylquinic acid） (interacts with) CA2	FALSE	interacts with	neochlorogenic acid（5-caffeoylquinic acid） (interacts with) CA2	
**Aldose reductase**	726.09207605	interacts with	chlorogenic acid (interacts with) AKR1B1	FALSE	interacts with	chlorogenic acid (interacts with) AKR1B1	绿原酸
**Cyclooxygenase-2**	121.10251791	interacts with	chlorogenic acid (interacts with) PTGS2	FALSE	interacts with	chlorogenic acid (interacts with) PTGS2	
**LDL-associated phospholipase A2**	128.14216277	interacts with	chlorogenic acid (interacts with) PLA2G7	FALSE	interacts with	chlorogenic acid (interacts with) PLA2G7	
**Signal transducer and activator of transcription 1-alpha/beta**	277.06666667	interacts with	chlorogenic acid (interacts with) STAT1	FALSE	interacts with	chlorogenic acid (interacts with) STAT1	
**Signal transducer and activator of transcription 3**	277.06666667	interacts with	chlorogenic acid (interacts with) STAT3	FALSE	interacts with	chlorogenic acid (interacts with) STAT3	
**Tyrosine-protein kinase LCK**	136.19191645	interacts with	chlorogenic acid (interacts with) LCK	FALSE	interacts with	chlorogenic acid (interacts with) LCK	
**VEGF-receptor 2 and tyrosine-protein kinase SRC**	277.06666667	interacts with	chlorogenic acid (interacts with) SRC	FALSE	interacts with	chlorogenic acid (interacts with) SRC	
**Aldo-keto reductase family 1 member B10**	112.24890939	interacts with	chlorogenic acid (interacts with) AKR1B10	FALSE	interacts with	chlorogenic acid (interacts with) AKR1B10	
**Geminin**	261.09773824	interacts with	chlorogenic acid (interacts with) GMNN	FALSE	interacts with	chlorogenic acid (interacts with) GMNN	
**Glutaminase kidney isoform, mitochondrial**	173.6402792	interacts with	chlorogenic acid (interacts with) GLS	FALSE	interacts with	chlorogenic acid (interacts with) GLS	
**Histone-lysine N-methyltransferase, H3 lysine-9 specific 3**	121.10251791	interacts with	chlorogenic acid (interacts with) EHMT2	FALSE	interacts with	chlorogenic acid (interacts with) EHMT2	
**Lysine-specific demethylase 4A**	270.03222797	interacts with	chlorogenic acid (interacts with) KDM4A	FALSE	interacts with	chlorogenic acid (interacts with) KDM4A	
**Signal transducer and activator of transcription 5B**	277.06666667	interacts with	chlorogenic acid (interacts with) STAT5B	FALSE	interacts with	chlorogenic acid (interacts with) STAT5B	
**UDP-glucuronosyltransferase 1-6**	225.31845462	interacts with	chlorogenic acid (interacts with) UGT1A6	FALSE	interacts with	chlorogenic acid (interacts with) UGT1A6	
**UDP-glucuronosyltransferase 1-9**	225.31845462	interacts with	chlorogenic acid (interacts with) UGT1A9	FALSE	interacts with	chlorogenic acid (interacts with) UGT1A9	
**Carbonic anhydrase VB**	102.36544163	interacts with	chlorogenic acid (interacts with) CA5B	FALSE	interacts with	chlorogenic acid (interacts with) CA5B	
**Carbonic anhydrase V**	176.20431054	interacts with	chlorogenic acid (interacts with) CA5A	FALSE	interacts with	chlorogenic acid (interacts with) CA5A	
**Carbonic anhydrase VI**	191.05089754	interacts with	chlorogenic acid (interacts with) CA6	FALSE	interacts with	chlorogenic acid (interacts with) CA6	
**Carbonic anhydrase VII**	119.20910802	interacts with	chlorogenic acid (interacts with) CA7	FALSE	interacts with	chlorogenic acid (interacts with) CA7	
**Matrix metalloproteinase 12**	126.54291776	interacts with	chlorogenic acid (interacts with) MMP12	FALSE	interacts with	chlorogenic acid (interacts with) MMP12	
**Carbonic anhydrase XIV**	135.35148678	interacts with	chlorogenic acid (interacts with) CA14	FALSE	interacts with	chlorogenic acid (interacts with) CA14	
**Aldose reductase**	726.09207605	interacts with	chlorogenic acid (interacts with) AKR1B1	FALSE	interacts with	chlorogenic acid (interacts with) AKR1B1	
**Carbonic anhydrase XII**	211.39363213	interacts with	chlorogenic acid (interacts with) CA12	FALSE	interacts with	chlorogenic acid (interacts with) CA12	
**Matrix metalloproteinase 9**	111.09936271	interacts with	chlorogenic acid (interacts with) MMP9	FALSE	interacts with	chlorogenic acid (interacts with) MMP9	
**Protein-tyrosine phosphatase 1B**	102.36544163	interacts with	chlorogenic acid (interacts with) PTPN1	FALSE	interacts with	chlorogenic acid (interacts with) PTPN1	
**Matrix metalloproteinase-2**	125.23409163	interacts with	chlorogenic acid (interacts with) MMP2	FALSE	interacts with	chlorogenic acid (interacts with) MMP2	
**Carbonic anhydrases; II & IX**	140.48384046	interacts with	chlorogenic acid (interacts with) CA9	FALSE	interacts with	chlorogenic acid (interacts with) CA9	
**Collagenase**	126.54291776	interacts with	chlorogenic acid (interacts with) MMP13	FALSE	interacts with	chlorogenic acid (interacts with) MMP13	
**Carbonic anhydrase I**	135.35148678	interacts with	chlorogenic acid (interacts with) CA1	FALSE	interacts with	chlorogenic acid (interacts with) CA1	
**Matrix metalloproteinase (1 and 13)**	111.09936271	interacts with	chlorogenic acid (interacts with) MMP1	FALSE	interacts with	chlorogenic acid (interacts with) MMP1	
**Carbonic anhydrase II**	155.3976224	interacts with	chlorogenic acid (interacts with) CA2	FALSE	interacts with	chlorogenic acid (interacts with) CA2	
**Carbonic anhydrase VB**	60.7813479	interacts with	cryptochlorogenic acid（4-Dicaffeoylquinic Acid） (interacts with) CA5B	FALSE	interacts with	cryptochlorogenic acid（4-Dicaffeoylquinic Acid） (interacts with) CA5B	隐绿原酸（4-咖啡酰奎宁酸）
**Carbonic anhydrase V**	98.75591159	interacts with	cryptochlorogenic acid（4-Dicaffeoylquinic Acid） (interacts with) CA5A	FALSE	interacts with	cryptochlorogenic acid（4-Dicaffeoylquinic Acid） (interacts with) CA5A	
**Carbonic anhydrase VI**	103.4095098	interacts with	cryptochlorogenic acid（4-Dicaffeoylquinic Acid） (interacts with) CA6	FALSE	interacts with	cryptochlorogenic acid（4-Dicaffeoylquinic Acid） (interacts with) CA6	
**Carbonic anhydrase VII**	64.5241791	interacts with	cryptochlorogenic acid（4-Dicaffeoylquinic Acid） (interacts with) CA7	FALSE	interacts with	cryptochlorogenic acid（4-Dicaffeoylquinic Acid） (interacts with) CA7	
**Carbonic anhydrase XIV**	68.48031274	interacts with	cryptochlorogenic acid（4-Dicaffeoylquinic Acid） (interacts with) CA14	FALSE	interacts with	cryptochlorogenic acid（4-Dicaffeoylquinic Acid） (interacts with) CA14	
**Matrix metalloproteinase 12**	74.45486648	interacts with	cryptochlorogenic acid（4-Dicaffeoylquinic Acid） (interacts with) MMP	FALSE	interacts with	cryptochlorogenic acid（4-Dicaffeoylquinic Acid） (interacts with) MMP12	
**Aldose reductase**	338.92155132	interacts with	cryptochlorogenic acid（4-Dicaffeoylquinic Acid） (interacts with) AKR1	FALSE	interacts with	cryptochlorogenic acid（4-Dicaffeoylquinic Acid） (interacts with) AKR1B1	
**Carbonic anhydrase XII**	107.49550531	interacts with	cryptochlorogenic acid（4-Dicaffeoylquinic Acid） (interacts with) CA12	FALSE	interacts with	cryptochlorogenic acid（4-Dicaffeoylquinic Acid） (interacts with) CA12	
**Matrix metalloproteinase 9**	62.38819138	interacts with	cryptochlorogenic acid（4-Dicaffeoylquinic Acid） (interacts with) MMP9	FALSE	interacts with	cryptochlorogenic acid（4-Dicaffeoylquinic Acid） (interacts with) MMP9	
**Protein-tyrosine phosphatase 1B**	60.7813479	interacts with	cryptochlorogenic acid（4-Dicaffeoylquinic Acid） (interacts with) PTPN	FALSE	interacts with	cryptochlorogenic acid（4-Dicaffeoylquinic Acid） (interacts with) PTPN1	
**Carbonic anhydrases; II & IX**	69.1481804	interacts with	cryptochlorogenic acid（4-Dicaffeoylquinic Acid） (interacts with) CA9	FALSE	interacts with	cryptochlorogenic acid（4-Dicaffeoylquinic Acid） (interacts with) CA9	
**Matrix metalloproteinase-2**	69.20875277	interacts with	cryptochlorogenic acid（4-Dicaffeoylquinic Acid） (interacts with) MMP2	FALSE	interacts with	cryptochlorogenic acid（4-Dicaffeoylquinic Acid） (interacts with) MMP2	
**Collagenase**	74.45486648	interacts with	cryptochlorogenic acid（4-Dicaffeoylquinic Acid） (interacts with) MMP	FALSE	interacts with	cryptochlorogenic acid（4-Dicaffeoylquinic Acid） (interacts with) MMP13	
**Carbonic anhydrase I**	68.48031274	interacts with	cryptochlorogenic acid（4-Dicaffeoylquinic Acid） (interacts with) CA1	FALSE	interacts with	cryptochlorogenic acid（4-Dicaffeoylquinic Acid） (interacts with) CA1	
**Matrix metalloproteinase (1 and 13)**	62.38819138	interacts with	cryptochlorogenic acid（4-Dicaffeoylquinic Acid） (interacts with) MMP	FALSE	interacts with	cryptochlorogenic acid（4-Dicaffeoylquinic Acid） (interacts with) MMP1	
**Carbonic anhydrase II**	75.24493984	interacts with	cryptochlorogenic acid（4-Dicaffeoylquinic Acid） (interacts with) CA2	FALSE	interacts with	cryptochlorogenic acid（4-Dicaffeoylquinic Acid） (interacts with) CA2	
**Acidic mammalian chitinase**	554.0	interacts with	genipin-1-β-D-gentiobioside (interacts with) CHIA	FALSE	interacts with	genipin-1-β-D-gentiobioside (interacts with) CHIA	京尼平-1-β-D-龙胆二糖苷
**Adenosine A1 receptor (by homology)**	618.25073601	interacts with	genipin-1-β-D-gentiobioside (interacts with) ADORA1	FALSE	interacts with	genipin-1-β-D-gentiobioside (interacts with) ADORA1	
**Adenosine A2a receptor (by homology)**	332.95883895	interacts with	genipin-1-β-D-gentiobioside (interacts with) ADORA2A	FALSE	interacts with	genipin-1-β-D-gentiobioside (interacts with) ADORA2A	
**Carbonic anhydrase I**	266.11047221	interacts with	genipin-1-β-D-gentiobioside (interacts with) CA1	FALSE	interacts with	genipin-1-β-D-gentiobioside (interacts with) CA1	
**Carbonic anhydrase II**	295.40043896	interacts with	genipin-1-β-D-gentiobioside (interacts with) CA2	FALSE	interacts with	genipin-1-β-D-gentiobioside (interacts with) CA2	
**Carbonic anhydrase IX**	279.76655485	interacts with	genipin-1-β-D-gentiobioside (interacts with) CA9	FALSE	interacts with	genipin-1-β-D-gentiobioside (interacts with) CA9	
**Carbonic anhydrase XII**	435.7665274	interacts with	genipin-1-β-D-gentiobioside (interacts with) CA12	FALSE	interacts with	genipin-1-β-D-gentiobioside (interacts with) CA12	
**Carbonic anhydrase XIV**	266.11047221	interacts with	genipin-1-β-D-gentiobioside (interacts with) CA14	FALSE	interacts with	genipin-1-β-D-gentiobioside (interacts with) CA14	
**Epoxide hydratase**	554.0	interacts with	genipin-1-β-D-gentiobioside (interacts with) EPHX2	FALSE	interacts with	genipin-1-β-D-gentiobioside (interacts with) EPHX2	
**Galectin-3**	554.0	interacts with	genipin-1-β-D-gentiobioside (interacts with) LGALS3	FALSE	interacts with	genipin-1-β-D-gentiobioside (interacts with) LGALS3	
**Galectin-9**	554.0	interacts with	genipin-1-β-D-gentiobioside (interacts with) LGALS9	FALSE	interacts with	genipin-1-β-D-gentiobioside (interacts with) LGALS9	
**Sialidase 2**	626.26232054	interacts with	genipin-1-β-D-gentiobioside (interacts with) NEU2	FALSE	interacts with	genipin-1-β-D-gentiobioside (interacts with) NEU2	
**Tyrosinase**	735.56322236	interacts with	genipin-1-β-D-gentiobioside (interacts with) TYR	FALSE	interacts with	genipin-1-β-D-gentiobioside (interacts with) TYR	
**Carboxy-terminal domain RNA polymerase II polypeptide A small phosphatase 1**	4.0	interacts with	geniposide (interacts with) CTDSP1	FALSE	interacts with	geniposide (interacts with) CTDSP1	栀子苷（京尼平苷）
**Gamma-amino-N-butyrate transaminase**	554.0	interacts with	p-Hydroxyacetophenone (interacts with) ABAT	FALSE	interacts with	p-Hydroxyacetophenone (interacts with) ABAT	对羟基苯乙酮
**Succinate semialdehyde dehydrogenase**	554.0	interacts with	p-Hydroxyacetophenone (interacts with) ALDH5A1	FALSE	interacts with	p-Hydroxyacetophenone (interacts with) ALDH5A1	
**Estradiol 17-beta-dehydrogenase 3**	326.75677375	interacts with	p-Hydroxyacetophenone (interacts with) HSD17B3	FALSE	interacts with	p-Hydroxyacetophenone (interacts with) HSD17B3	
**UDP-glucuronosyltransferase 1-6**	166.08301568	interacts with	p-Hydroxyacetophenone (interacts with) UGT1A6	FALSE	interacts with	p-Hydroxyacetophenone (interacts with) UGT1A6	
**UDP-glucuronosyltransferase 1-9**	166.08301568	interacts with	p-Hydroxyacetophenone (interacts with) UGT1A9	FALSE	interacts with	p-Hydroxyacetophenone (interacts with) UGT1A9	
**Aldehyde oxidase**	554.0	interacts with	p-Hydroxyacetophenone (interacts with) AOX1	FALSE	interacts with	p-Hydroxyacetophenone (interacts with) AOX1	
**Carbonic anhydrase**	531.70496186	interacts with	p-Hydroxyacetophenone (interacts with) CA3	FALSE	interacts with	p-Hydroxyacetophenone (interacts with) CA3	
**Carbonic anhydrase VII**	185.75125659	interacts with	p-Hydroxyacetophenone (interacts with) CA7	FALSE	interacts with	p-Hydroxyacetophenone (interacts with) CA7	
**Carbonic anhydrase V**	551.87864089	interacts with	p-Hydroxyacetophenone (interacts with) CA5A	FALSE	interacts with	p-Hydroxyacetophenone (interacts with) CA5A	
**Carbonic anhydrase VI**	570.0658033	interacts with	p-Hydroxyacetophenone (interacts with) CA6	FALSE	interacts with	p-Hydroxyacetophenone (interacts with) CA6	
**Carbonic anhydrase XIV**	221.7258907	interacts with	p-Hydroxyacetophenone (interacts with) CA14	FALSE	interacts with	p-Hydroxyacetophenone (interacts with) CA14	
**Carbonic anhydrase XIII**	337.19513852	interacts with	p-Hydroxyacetophenone (interacts with) CA13	FALSE	interacts with	p-Hydroxyacetophenone (interacts with) CA13	
**Nucleotide-binding oligomerization domain-containing protein 2**	554.0	interacts with	p-Hydroxyacetophenone (interacts with) NOD2	FALSE	interacts with	p-Hydroxyacetophenone (interacts with) NOD2	
**Carbonic anhydrase XII**	452.50378635	interacts with	p-Hydroxyacetophenone (interacts with) CA12	FALSE	interacts with	p-Hydroxyacetophenone (interacts with) CA12	
**G-protein coupled receptor 55**	554.0	interacts with	p-Hydroxyacetophenone (interacts with) GPR55	FALSE	interacts with	p-Hydroxyacetophenone (interacts with) GPR55	

**Carbonic anhydrase I**	221.7258907	interacts with	p-Hydroxyacetophenone (interacts with) CA1	FALSE	interacts with	p-Hydroxyacetophenone (interacts with) CA1	
**Aldo-keto-reductase family 1 member C3**	166.45313486	interacts with	p-Hydroxyacetophenone (interacts with) AKR1C3	FALSE	interacts with	p-Hydroxyacetophenone (interacts with) AKR1C3	
**Estrogen receptor alpha**	714.64581174	interacts with	p-Hydroxyacetophenone (interacts with) ESR1	FALSE	interacts with	p-Hydroxyacetophenone (interacts with) ESR1	
**Carbonic anhydrase II**	286.38142219	interacts with	p-Hydroxyacetophenone (interacts with) CA2	FALSE	interacts with	p-Hydroxyacetophenone (interacts with) CA2	
**Carbonic anhydrases; II & IX**	265.22546197	interacts with	p-Hydroxyacetophenone (interacts with) CA9	FALSE	interacts with	p-Hydroxyacetophenone (interacts with) CA9	
**TNF-alpha**	554.0	interacts with	p-Hydroxyacetophenone (interacts with) TNF	FALSE	interacts with	p-Hydroxyacetophenone (interacts with) TNF	
**ATP-binding cassette sub-family G member 2**	617.18943679	interacts with	p-Hydroxyacetophenone (interacts with) ABCG2	FALSE	interacts with	p-Hydroxyacetophenone (interacts with) ABCG2	
**Aldo-keto reductase family 1 member C2**	166.45313486	interacts with	p-Hydroxyacetophenone (interacts with) AKR1C2	FALSE	interacts with	p-Hydroxyacetophenone (interacts with) AKR1C2	
**Nuclear receptor subfamily 0 group B member 1**	166.05589016	interacts with	scutellarin (interacts with) NR0B1	FALSE	interacts with	scutellarin (interacts with) NR0B1	野黄芩苷
**Intestinal alkaline phosphatase**	255.72367493	interacts with	scutellarin (interacts with) ALPI	FALSE	interacts with	scutellarin (interacts with) ALPI	
**GABA-A receptor; anion channel**	192.0447686	interacts with	scutellarin (interacts with) GABRG2	FALSE	interacts with	scutellarin (interacts with) GABRG2	
**FAD-linked sulfhydryl oxidase ALR**	91.4130126	interacts with	scutellarin (interacts with) GFER	FALSE	interacts with	scutellarin (interacts with) GFER	
**Neurotensin receptor**	135.95054598	interacts with	scutellarin (interacts with) NTSR1	FALSE	interacts with	scutellarin (interacts with) NTSR1	
**Adenosine A2a receptor**	180.6627955	interacts with	scutellarin (interacts with) ADORA2A	FALSE	interacts with	scutellarin (interacts with) ADORA2A	
**Adenosine receptors; A1 & A2a**	189.03521017	interacts with	scutellarin (interacts with) ADORA1	FALSE	interacts with	scutellarin (interacts with) ADORA1	
**Aldose reductase**	350.04442971	interacts with	Isochlorogenic acid B (interacts with) AKR1B1	FALSE	interacts with	Isochlorogenic acid B (interacts with) AKR1B1	异绿原酸B（3,4-二咖啡酰奎宁
**Matrix metalloproteinase 12**	71.17472828	interacts with	Isochlorogenic acid B (interacts with) MMP12	FALSE	interacts with	Isochlorogenic acid B (interacts with) MMP12	
**Matrix metalloproteinase-2**	94.89218523	interacts with	Isochlorogenic acid B (interacts with) MMP2	FALSE	interacts with	Isochlorogenic acid B (interacts with) MMP2	
**Collagenase**	71.17472828	interacts with	Isochlorogenic acid B (interacts with) MMP13	FALSE	interacts with	Isochlorogenic acid B (interacts with) MMP13	
**Aldo-keto reductase family 1 member B10**	65.89397856	interacts with	Isochlorogenic acid B (interacts with) AKR1B10	FALSE	interacts with	Isochlorogenic acid B (interacts with) AKR1B10	
**Aldose reductase**	355.3374327	interacts with	Isochlorogenic acid A (interacts with) AKR1B1	FALSE	interacts with	Isochlorogenic acid A (interacts with) AKR1B1	异绿原酸A（3,5-二咖啡酰奎宁
**Aldo-keto reductase family 1 member B10**	67.95356518	interacts with	Isochlorogenic acid A (interacts with) AKR1B10	FALSE	interacts with	Isochlorogenic acid A (interacts with) AKR1B10	
**Carbonic anhydrase VB**	60.89514783	interacts with	Isochlorogenic acid A (interacts with) CA5B	FALSE	interacts with	Isochlorogenic acid A (interacts with) CA5B	
**Carbonic anhydrase V**	102.86159453	interacts with	Isochlorogenic acid A (interacts with) CA5A	FALSE	interacts with	Isochlorogenic acid A (interacts with) CA5A	
**Carbonic anhydrase VI**	108.10313032	interacts with	Isochlorogenic acid A (interacts with) CA6	FALSE	interacts with	Isochlorogenic acid A (interacts with) CA6	
**Carbonic anhydrase VII**	65.69251454	interacts with	Isochlorogenic acid A (interacts with) CA7	FALSE	interacts with	Isochlorogenic acid A (interacts with) CA7	
**Matrix metalloproteinase 12**	76.95051586	interacts with	Isochlorogenic acid A (interacts with) MMP12	FALSE	interacts with	Isochlorogenic acid A (interacts with) MMP12	
**Carbonic anhydrase XIV**	70.3705984	interacts with	Isochlorogenic acid A (interacts with) CA14	FALSE	interacts with	Isochlorogenic acid A (interacts with) CA14	
**Aldose reductase**	355.3374327	interacts with	Isochlorogenic acid A (interacts with) AKR1B1	FALSE	interacts with	Isochlorogenic acid A (interacts with) AKR1B1	
**Carbonic anhydrase XII**	112.08320769	interacts with	Isochlorogenic acid A (interacts with) CA12	FALSE	interacts with	Isochlorogenic acid A (interacts with) CA12	
**Matrix metalloproteinase 9**	64.08633419	interacts with	Isochlorogenic acid A (interacts with) MMP9	FALSE	interacts with	Isochlorogenic acid A (interacts with) MMP9	
**Matrix metalloproteinase-2**	70.82189169	interacts with	Isochlorogenic acid A (interacts with) MMP2	FALSE	interacts with	Isochlorogenic acid A (interacts with) MMP2	
**Protein-tyrosine phosphatase 1B**	60.89514783	interacts with	Isochlorogenic acid A (interacts with) PTPN1	FALSE	interacts with	Isochlorogenic acid A (interacts with) PTPN1	
**Collagenase**	76.95051586	interacts with	Isochlorogenic acid A (interacts with) MMP13	FALSE	interacts with	Isochlorogenic acid A (interacts with) MMP13	
**Carbonic anhydrases; II & IX**	71.42573373	interacts with	Isochlorogenic acid A (interacts with) CA9	FALSE	interacts with	Isochlorogenic acid A (interacts with) CA9	
**Carbonic anhydrase I**	70.3705984	interacts with	Isochlorogenic acid A (interacts with) CA1	FALSE	interacts with	Isochlorogenic acid A (interacts with) CA1	
**Matrix metalloproteinase (1 and 13)**	64.08633419	interacts with	Isochlorogenic acid A (interacts with) MMP1	FALSE	interacts with	Isochlorogenic acid A (interacts with) MMP1	
**Carbonic anhydrase II**	77.75199943	interacts with	Isochlorogenic acid A (interacts with) CA2	FALSE	interacts with	Isochlorogenic acid A (interacts with) CA2	
**Aldose reductase**	349.27549057	interacts with	Isochlorogenic acid C (interacts with) AKR1B1	FALSE	interacts with	Isochlorogenic acid C (interacts with) AKR1B1	异绿原酸C（4,5-二咖啡酰奎宁
**Matrix metalloproteinase 12**	73.09704165	interacts with	Isochlorogenic acid C (interacts with) MMP12	FALSE	interacts with	Isochlorogenic acid C (interacts with) MMP12	
**Matrix metalloproteinase-2**	106.95939444	interacts with	Isochlorogenic acid C (interacts with) MMP2	FALSE	interacts with	Isochlorogenic acid C (interacts with) MMP2	
**Collagenase**	73.09704165	interacts with	Isochlorogenic acid C (interacts with) MMP13	FALSE	interacts with	Isochlorogenic acid C (interacts with) MMP13	
**Cyclin-dependent kinase 1**	148.18696526	interacts with	Baicalin (interacts with) CDK1	FALSE	interacts with	Baicalin (interacts with) CDK1	黄芩苷
**Dipeptidyl peptidase II and dipeptidyl peptidase IV (DPP2 and DPP4)**	148.18696526	interacts with	Baicalin (interacts with) DPP4	FALSE	interacts with	Baicalin (interacts with) DPP4	
**Estrogen receptor alpha**	967.76252437	interacts with	Baicalin (interacts with) ESR1	FALSE	interacts with	Baicalin (interacts with) ESR1	
**Canalicular multispecific organic anion transporter 1**	554.0	interacts with	Baicalin (interacts with) ABCC2	FALSE	interacts with	Baicalin (interacts with) ABCC2	
**GABA-A receptor; anion channel**	466.45186211	interacts with	Baicalin (interacts with) GABRG2	FALSE	interacts with	Baicalin (interacts with) GABRG2	
**Prolyl endopeptidase**	148.18696526	interacts with	Baicalin (interacts with) PREP	FALSE	interacts with	Baicalin (interacts with) PREP	
**Serotonin (5-HT3) receptor**	326.03904135	interacts with	Baicalin (interacts with) HTR7	FALSE	interacts with	Baicalin (interacts with) HTR7	
**Solute carrier organic anion transporter family member 1B1**	554.0	interacts with	Baicalin (interacts with) SLCO1B1	FALSE	interacts with	Baicalin (interacts with) SLCO1B1	
**Solute carrier organic anion transporter family member 1B3**	554.0	interacts with	Baicalin (interacts with) SLCO1B3	FALSE	interacts with	Baicalin (interacts with) SLCO1B3	
**Solute carrier organic anion transporter family member 2B1**	554.0	interacts with	Baicalin (interacts with) SLCO2B1	FALSE	interacts with	Baicalin (interacts with) SLCO2B1	
**Intestinal alkaline phosphatase**	621.26021024	interacts with	Baicalin (interacts with) ALPI	FALSE	interacts with	Baicalin (interacts with) ALPI	
**Sucrase-isomaltase**	196.00781475	interacts with	Baicalin (interacts with) SI	FALSE	interacts with	Baicalin (interacts with) SI	
**Adenosine A1 receptor (by homology)**	1008.07005885	interacts with	Oroxylin A-7-O-β-D-glucuronide (interacts with) ADORA1	FALSE	interacts with	Oroxylin A-7-O-β-D-glucuronide (interacts with) ADORA1	千层纸素A-7-O-β-D-葡萄糖醛
**Xanthine dehydrogenase**	2209.45876755	interacts with	Oroxylin A-7-O-β-D-glucuronide (interacts with) XDH	FALSE	interacts with	Oroxylin A-7-O-β-D-glucuronide (interacts with) XDH	
**Aldose reductase**	3524.13924425	interacts with	Oroxylin A-7-O-β-D-glucuronide (interacts with) AKR1B1	FALSE	interacts with	Oroxylin A-7-O-β-D-glucuronide (interacts with) AKR1B1	
**Interleukin-2**	554.0	interacts with	Oroxylin A-7-O-β-D-glucuronide (interacts with) IL2	FALSE	interacts with	Oroxylin A-7-O-β-D-glucuronide (interacts with) IL2	
**Sialidase 2**	1165.84802929	interacts with	Oroxylin A-7-O-β-D-glucuronide (interacts with) NEU2	FALSE	interacts with	Oroxylin A-7-O-β-D-glucuronide (interacts with) NEU2	
**Adenosine A3 receptor**	554.0	interacts with	Oroxylin A-7-O-β-D-glucuronide (interacts with) ADORA3	FALSE	interacts with	Oroxylin A-7-O-β-D-glucuronide (interacts with) ADORA3	
**Inosine-5'-monophosphate dehydrogenase 2**	471.09488992	interacts with	Oroxylin A-7-O-β-D-glucuronide (interacts with) IMPDH2	FALSE	interacts with	Oroxylin A-7-O-β-D-glucuronide (interacts with) IMPDH2	
**Hexokinase type II**	554.0	interacts with	Oroxylin A-7-O-β-D-glucuronide (interacts with) HK2	FALSE	interacts with	Oroxylin A-7-O-β-D-glucuronide (interacts with) HK2	
**Hexokinase type I**	554.0	interacts with	Oroxylin A-7-O-β-D-glucuronide (interacts with) HK1	FALSE	interacts with	Oroxylin A-7-O-β-D-glucuronide (interacts with) HK1	
**Adenosine A2a receptor**	437.0448233	interacts with	Oroxylin A-7-O-β-D-glucuronide (interacts with) ADORA2A	FALSE	interacts with	Oroxylin A-7-O-β-D-glucuronide (interacts with) ADORA2A	
**P-glycoprotein 1**	1152.56628962	interacts with	Oroxylin A-7-O-β-D-glucuronide (interacts with) ABCB1	FALSE	interacts with	Oroxylin A-7-O-β-D-glucuronide (interacts with) ABCB1	
**Squalene synthetase (by homology)**	554.0	interacts with	Oroxylin A-7-O-β-D-glucuronide (interacts with) FDFT1	FALSE	interacts with	Oroxylin A-7-O-β-D-glucuronide (interacts with) FDFT1	
**MAP kinase p38 alpha**	685.10312801	interacts with	Oroxylin A-7-O-β-D-glucuronide (interacts with) MAPK14	FALSE	interacts with	Oroxylin A-7-O-β-D-glucuronide (interacts with) MAPK14	
**Lysine-specific demethylase 4D**	554.0	interacts with	Oroxylin A-7-O-β-D-glucuronide (interacts with) KDM4D	FALSE	interacts with	Oroxylin A-7-O-β-D-glucuronide (interacts with) KDM4D	
**Lysine-specific demethylase 4C**	1138.13010855	interacts with	Oroxylin A-7-O-β-D-glucuronide (interacts with) KDM4C	FALSE	interacts with	Oroxylin A-7-O-β-D-glucuronide (interacts with) KDM4C	
**Ribosomal protein S6 kinase alpha 3**	608.36059989	interacts with	Oroxylin A-7-O-β-D-glucuronide (interacts with) RPS6KA3	FALSE	interacts with	Oroxylin A-7-O-β-D-glucuronide (interacts with) RPS6KA3	
**Epidermal growth factor receptor erbB1**	1177.84989456	interacts with	Oroxylin A-7-O-β-D-glucuronide (interacts with) EGFR	FALSE	interacts with	Oroxylin A-7-O-β-D-glucuronide (interacts with) EGFR	
**Sigma opioid receptor**	554.0	interacts with	Oroxylin A-7-O-β-D-glucuronide (interacts with) SIGMAR1	FALSE	interacts with	Oroxylin A-7-O-β-D-glucuronide (interacts with) SIGMAR1	
**Dopamine D2 receptor (by homology)**	554.0	interacts with	Oroxylin A-7-O-β-D-glucuronide (interacts with) DRD2	FALSE	interacts with	Oroxylin A-7-O-β-D-glucuronide (interacts with) DRD2	
**Neprilysin (by homology)**	554.0	interacts with	Oroxylin A-7-O-β-D-glucuronide (interacts with) MME	FALSE	interacts with	Oroxylin A-7-O-β-D-glucuronide (interacts with) MME	
**Mitogen-activated protein kinase kinase kinase 14**	554.0	interacts with	Oroxylin A-7-O-β-D-glucuronide (interacts with) MAP3K14	FALSE	interacts with	Oroxylin A-7-O-β-D-glucuronide (interacts with) MAP3K14	
**Sialidase 4**	554.0	interacts with	Oroxylin A-7-O-β-D-glucuronide (interacts with) NEU4	FALSE	interacts with	Oroxylin A-7-O-β-D-glucuronide (interacts with) NEU4	
**Caspase-3**	554.0	interacts with	Oroxylin A-7-O-β-D-glucuronide (interacts with) CASP3	FALSE	interacts with	Oroxylin A-7-O-β-D-glucuronide (interacts with) CASP3	
**Hypoxanthine-guanine phosphoribosyltransferase**	554.0	interacts with	Oroxylin A-7-O-β-D-glucuronide (interacts with) HPRT1	FALSE	interacts with	Oroxylin A-7-O-β-D-glucuronide (interacts with) HPRT1	
**Splicing factor 3B subunit 3**	554.0	interacts with	Oroxylin A-7-O-β-D-glucuronide (interacts with) SF3B3	FALSE	interacts with	Oroxylin A-7-O-β-D-glucuronide (interacts with) SF3B3	
**Proteasome Macropain subunit MB1**	554.0	interacts with	Oroxylin A-7-O-β-D-glucuronide (interacts with) PSMB5	FALSE	interacts with	Oroxylin A-7-O-β-D-glucuronide (interacts with) PSMB5	
**Thymidylate synthase (by homology)**	554.0	interacts with	Oroxylin A-7-O-β-D-glucuronide (interacts with) TYMS	FALSE	interacts with	Oroxylin A-7-O-β-D-glucuronide (interacts with) TYMS	
**Leucine aminopeptidase**	554.0	interacts with	Oroxylin A-7-O-β-D-glucuronide (interacts with) LAP3	FALSE	interacts with	Oroxylin A-7-O-β-D-glucuronide (interacts with) LAP3	
**Integrin alpha-V/beta-3**	554.0	interacts with	Oroxylin A-7-O-β-D-glucuronide (interacts with) ITGAV ITGB3	FALSE	interacts with	Oroxylin A-7-O-β-D-glucuronide (interacts with) ITGAV ITGB3	
**Niemann-Pick C1-like protein 1**	554.0	interacts with	Oroxylin A-7-O-β-D-glucuronide (interacts with) NPC1L1	FALSE	interacts with	Oroxylin A-7-O-β-D-glucuronide (interacts with) NPC1L1	
**Myelin-associated glycoprotein (by homology)**	554.0	interacts with	Oroxylin A-7-O-β-D-glucuronide (interacts with) MAG	FALSE	interacts with	Oroxylin A-7-O-β-D-glucuronide (interacts with) MAG	
**Aldehyde dehydrogenase**	675.3388262	interacts with	Oroxylin A-7-O-β-D-glucuronide (interacts with) ALDH2	FALSE	interacts with	Oroxylin A-7-O-β-D-glucuronide (interacts with) ALDH2	
**Receptor-type tyrosine-protein phosphatase S**	554.0	interacts with	Oroxylin A-7-O-β-D-glucuronide (interacts with) PTPRS	FALSE	interacts with	Oroxylin A-7-O-β-D-glucuronide (interacts with) PTPRS	
**Muscle glycogen phosphorylase**	554.0	interacts with	Oroxylin A-7-O-β-D-glucuronide (interacts with) PYGM	FALSE	interacts with	Oroxylin A-7-O-β-D-glucuronide (interacts with) PYGM	
**Caspase-1**	554.0	interacts with	Oroxylin A-7-O-β-D-glucuronide (interacts with) CASP1	FALSE	interacts with	Oroxylin A-7-O-β-D-glucuronide (interacts with) CASP1	
**Intestinal alkaline phosphatase**	154.53385357	interacts with	Wogonoside (interacts with) ALPI	FALSE	interacts with	Wogonoside (interacts with) ALPI	汉黄芩苷
**Aldose reductase**	428.62689071	interacts with	Wogonoside (interacts with) AKR1B1	FALSE	interacts with	Wogonoside (interacts with) AKR1B1	
**Androgen Receptor**	282.20344197	interacts with	Baicalein (interacts with) AR	FALSE	interacts with	Baicalein (interacts with) AR	黄芩素
**Arachidonate 5-lipoxygenase**	364.53290762	interacts with	Baicalein (interacts with) ALOX5	FALSE	interacts with	Baicalein (interacts with) ALOX5	
**Beta-glucocerebrosidase**	282.20344197	interacts with	Baicalein (interacts with) GBA	FALSE	interacts with	Baicalein (interacts with) GBA	
**Cyclin-dependent kinase 1**	619.59541221	interacts with	Baicalein (interacts with) CDK1	FALSE	interacts with	Baicalein (interacts with) CDK1	
**Cytochrome P450 2D6**	282.20344197	interacts with	Baicalein (interacts with) CYP2D6	FALSE	interacts with	Baicalein (interacts with) CYP2D6	
**Cytochrome P450 3A4**	351.47539622	interacts with	Baicalein (interacts with) CYP3A4	FALSE	interacts with	Baicalein (interacts with) CYP3A4	
**Dipeptidyl peptidase II and dipeptidyl peptidase IV (DPP2 and DPP4)**	619.59541221	interacts with	Baicalein (interacts with) DPP4	FALSE	interacts with	Baicalein (interacts with) DPP4	
**Estrogen receptor alpha**	640.47970365	interacts with	Baicalein (interacts with) ESR1	FALSE	interacts with	Baicalein (interacts with) ESR1	
**Glucocorticoid receptor**	282.20344197	interacts with	Baicalein (interacts with) NR3C1	FALSE	interacts with	Baicalein (interacts with) NR3C1	
**Hemoglobin beta chain**	554.0	interacts with	Baicalein (interacts with) HBB	FALSE	interacts with	Baicalein (interacts with) HBB	
**Lysosomal alpha-glucosidase**	459.24446285	interacts with	Baicalein (interacts with) GAA	FALSE	interacts with	Baicalein (interacts with) GAA	
**Maltase-glucoamylase**	610.95189872	interacts with	Baicalein (interacts with) MGAM	FALSE	interacts with	Baicalein (interacts with) MGAM	
**P-selectin**	907.63014281	interacts with	Baicalein (interacts with) SELP	FALSE	interacts with	Baicalein (interacts with) SELP	
**Selectin E**	554.0	interacts with	Baicalein (interacts with) SELE	FALSE	interacts with	Baicalein (interacts with) SELE	
**Transcription factor AP1**	452.55159634	interacts with	Baicalein (interacts with) JUN	FALSE	interacts with	Baicalein (interacts with) JUN	
**Tumour suppressor p53/oncoprotein Mdm2**	282.20344197	interacts with	Baicalein (interacts with) TP53	FALSE	interacts with	Baicalein (interacts with) TP53	
**Tyrosine-protein kinase receptor FLT3**	259.09700735	interacts with	Baicalein (interacts with) FLT3	FALSE	interacts with	Baicalein (interacts with) FLT3	
**Xanthine dehydrogenase**	859.66277185	interacts with	Baicalein (interacts with) XDH	FALSE	interacts with	Baicalein (interacts with) XDH	
**15-hydroxyprostaglandin dehydrogenase [NAD+]**	351.47539622	interacts with	Baicalein (interacts with) HPGD	FALSE	interacts with	Baicalein (interacts with) HPGD	
**Aldehyde dehydrogenase 1A1**	351.47539622	interacts with	Baicalein (interacts with) ALDH1A1	FALSE	interacts with	Baicalein (interacts with) ALDH1A1	
**Arachidonate 12-lipoxygenase**	372.2826643	interacts with	Baicalein (interacts with) ALOX12	FALSE	interacts with	Baicalein (interacts with) ALOX12	
**Arachidonate 15-lipoxygenase**	282.20344197	interacts with	Baicalein (interacts with) ALOX15	FALSE	interacts with	Baicalein (interacts with) ALOX15	
**Arachidonate 15-lipoxygenase, type II**	282.20344197	interacts with	Baicalein (interacts with) ALOX15B	FALSE	interacts with	Baicalein (interacts with) ALOX15B	
**ATP-dependent DNA helicase Q1**	282.20344197	interacts with	Baicalein (interacts with) RECQL	FALSE	interacts with	Baicalein (interacts with) RECQL	

**ATPase family AAA domain-containing protein 5**	282.20344197	interacts with	Baicalein (interacts with) ATAD5	FALSE	interacts with	Baicalein (interacts with) ATAD5	
**Bloom syndrome protein**	282.20344197	interacts with	Baicalein (interacts with) BLM	FALSE	interacts with	Baicalein (interacts with) BLM	
**Breast cancer type 1 susceptibility protein**	282.20344197	interacts with	Baicalein (interacts with) BRCA1	FALSE	interacts with	Baicalein (interacts with) BRCA1	
**Cyclic AMP-responsive element-binding protein 1**	452.55159634	interacts with	Baicalein (interacts with) CREB1	FALSE	interacts with	Baicalein (interacts with) CREB1	
**Cytochrome P450 1A**	259.09700735	interacts with	Baicalein (interacts with) CYP1A1	FALSE	interacts with	Baicalein (interacts with) CYP1A1	
**Cytochrome P450 1A2**	282.20344197	interacts with	Baicalein (interacts with) CYP1A2	FALSE	interacts with	Baicalein (interacts with) CYP1A2	
**Cytochrome P450 1B1**	372.2826643	interacts with	Baicalein (interacts with) CYP1B1	FALSE	interacts with	Baicalein (interacts with) CYP1B1	
**Cytochrome P450 2C19**	282.20344197	interacts with	Baicalein (interacts with) CYP2C19	FALSE	interacts with	Baicalein (interacts with) CYP2C19	
**Cytochrome P450 2C9**	282.20344197	interacts with	Baicalein (interacts with) CYP2C9	FALSE	interacts with	Baicalein (interacts with) CYP2C9	
**DNA polymerase eta**	372.2826643	interacts with	Baicalein (interacts with) POLH	FALSE	interacts with	Baicalein (interacts with) POLH	
**DNA polymerase iota**	372.2826643	interacts with	Baicalein (interacts with) POLI	FALSE	interacts with	Baicalein (interacts with) POLI	
**DNA polymerase kapp**	459.24446285	interacts with	Baicalein (interacts with) POLK	FALSE	interacts with	Baicalein (interacts with) POLK	
**DNA-(apurinic or apyrimidinic site) lyase**	459.24446285	interacts with	Baicalein (interacts with) APEX1	FALSE	interacts with	Baicalein (interacts with) APEX1	
**Egl nine homolog 1**	427.80188668	interacts with	Baicalein (interacts with) EGLN1	FALSE	interacts with	Baicalein (interacts with) EGLN1	
**Endoplasmic reticulum-associated amyloid beta-peptide-binding protei**	351.47539622	interacts with	Baicalein (interacts with) HSD17B10	FALSE	interacts with	Baicalein (interacts with) HSD17B10	
**Estradiol 17-beta-dehydrogenase 3**	633.3242027	interacts with	Baicalein (interacts with) HSD17B3	FALSE	interacts with	Baicalein (interacts with) HSD17B3	
**Flap endonuclease 1**	372.2826643	interacts with	Baicalein (interacts with) FEN1	FALSE	interacts with	Baicalein (interacts with) FEN1	
**G-protein coupled receptor 35**	282.20344197	interacts with	Baicalein (interacts with) GPR35	FALSE	interacts with	Baicalein (interacts with) GPR35	
**GABA-A receptor; anion channel**	390.80159796	interacts with	Baicalein (interacts with) GABRG2	FALSE	interacts with	Baicalein (interacts with) GABRG2	
**Geminin**	526.4783597	interacts with	Baicalein (interacts with) GMNN	FALSE	interacts with	Baicalein (interacts with) GMNN	
**Glyoxalase I**	282.20344197	interacts with	Baicalein (interacts with) GLO1	FALSE	interacts with	Baicalein (interacts with) GLO1	
**Histone-lysine N-methyltransferase MLL**	351.47539622	interacts with	Baicalein (interacts with) KMT2A	FALSE	interacts with	Baicalein (interacts with) KMT2A	
**Hypoxia-inducible factor 1-alpha inhibitor**	554.0	interacts with	Baicalein (interacts with) HIF1AN	FALSE	interacts with	Baicalein (interacts with) HIF1AN	
**Lethal(3)malignant brain tumor-like protein 1**	554.0	interacts with	Baicalein (interacts with) L3MBTL1	FALSE	interacts with	Baicalein (interacts with) L3MBTL1	
**Leukocyte adhesion molecule-1**	907.63014281	interacts with	Baicalein (interacts with) SELL	FALSE	interacts with	Baicalein (interacts with) SELL	
**Lysine-specific demethylase 4A**	663.34276013	interacts with	Baicalein (interacts with) KDM4A	FALSE	interacts with	Baicalein (interacts with) KDM4A	
**Lysine-specific demethylase 4D-like**	459.24446285	interacts with	Baicalein (interacts with) KDM4E	FALSE	interacts with	Baicalein (interacts with) KDM4E	
**Microtubule-associated protein tau**	351.47539622	interacts with	Baicalein (interacts with) MAPT	FALSE	interacts with	Baicalein (interacts with) MAPT	
**P-glycoprotein 1**	623.74902717	interacts with	Baicalein (interacts with) ABCB1	FALSE	interacts with	Baicalein (interacts with) ABCB1	
**Peptidyl-prolyl cis-trans isomerase NIMA-interacting 1**	282.20344197	interacts with	Baicalein (interacts with) PIN1	FALSE	interacts with	Baicalein (interacts with) PIN1	
**Prolyl endopeptidase**	619.59541221	interacts with	Baicalein (interacts with) PREP	FALSE	interacts with	Baicalein (interacts with) PREP	
**Runt-related transcription factor 1/Core-binding factor subunit beta**	372.2826643	interacts with	Baicalein (interacts with) RUNX1	FALSE	interacts with	Baicalein (interacts with) RUNX1	
**Serine/threonine-protein kinase PIM1**	282.20344197	interacts with	Baicalein (interacts with) PIM1	FALSE	interacts with	Baicalein (interacts with) PIM1	
**Serotonin (5-HT3) receptor**	555.32932223	interacts with	Baicalein (interacts with) HTR7	FALSE	interacts with	Baicalein (interacts with) HTR7	
**Sialidase 2**	790.49380938	interacts with	Baicalein (interacts with) NEU2	FALSE	interacts with	Baicalein (interacts with) NEU2	
**Sucrase-isomaltase**	530.04686894	interacts with	Baicalein (interacts with) SI	FALSE	interacts with	Baicalein (interacts with) SI	
**Survival motor neuron protein**	372.2826643	interacts with	Baicalein (interacts with) SMN1	FALSE	interacts with	Baicalein (interacts with) SMN1	
**Thyroid hormone receptor beta-1**	351.47539622	interacts with	Baicalein (interacts with) THRB	FALSE	interacts with	Baicalein (interacts with) THRB	
**Ubiquitin carboxyl-terminal hydrolase 2**	554.0	interacts with	Baicalein (interacts with) USP2	FALSE	interacts with	Baicalein (interacts with) USP2	
**UDP-glucuronosyltransferase 1-1**	554.0	interacts with	Baicalein (interacts with) UGT1A1	FALSE	interacts with	Baicalein (interacts with) UGT1A1	
**UDP-glucuronosyltransferase 2B15**	554.0	interacts with	Baicalein (interacts with) UGT2B15	FALSE	interacts with	Baicalein (interacts with) UGT2B15	
**Werner syndrome ATP-dependent helicase**	282.20344197	interacts with	Baicalein (interacts with) WRN	FALSE	interacts with	Baicalein (interacts with) WRN	
**Cytochrome P450 1A1**	259.09700735	interacts with	Baicalein (interacts with) CYP1A1	FALSE	interacts with	Baicalein (interacts with) CYP1A1	
**Nuclear receptor subfamily 0 group B member 1**	295.36642794	interacts with	Baicalein (interacts with) NR0B1	FALSE	interacts with	Baicalein (interacts with) NR0B1	
**MAP kinase ERK1**	452.55159634	interacts with	Baicalein (interacts with) MAPK3	FALSE	interacts with	Baicalein (interacts with) MAPK3	
**GABA-A receptor; anion channel**	390.80159796	interacts with	Baicalein (interacts with) GABRG2	FALSE	interacts with	Baicalein (interacts with) GABRG2	
**Tankyrase-1**	259.09700735	interacts with	Baicalein (interacts with) TNKS	FALSE	interacts with	Baicalein (interacts with) TNKS	
**Aldose reductase**	1611.41007051	interacts with	Baicalein (interacts with) AKR1B1	FALSE	interacts with	Baicalein (interacts with) AKR1B1	
**Epidermal growth factor receptor erbB1**	744.80037524	interacts with	Baicalein (interacts with) EGFR	FALSE	interacts with	Baicalein (interacts with) EGFR	
**Aryl hydrocarbon receptor**	452.55159634	interacts with	Baicalein (interacts with) AHR	FALSE	interacts with	Baicalein (interacts with) AHR	
**FAD-linked sulfhydryl oxidase ALR**	426.31213762	interacts with	Baicalein (interacts with) GFER	FALSE	interacts with	Baicalein (interacts with) GFER	
**Sucrase-isomaltase**	530.04686894	interacts with	Baicalein (interacts with) SI	FALSE	interacts with	Baicalein (interacts with) SI	
**Tankyrase-2**	259.09700735	interacts with	Baicalein (interacts with) TNKS2	FALSE	interacts with	Baicalein (interacts with) TNKS2	
**GABA receptor delta subunit**	452.55159634	interacts with	Baicalein (interacts with) GABRD	FALSE	interacts with	Baicalein (interacts with) GABRD	
**Nitric oxide synthase, inducible**	452.55159634	interacts with	Baicalein (interacts with) NOS2	FALSE	interacts with	Baicalein (interacts with) NOS2	
**Cytochrome P450 1A2**	282.20344197	interacts with	Baicalein (interacts with) CYP1A2	FALSE	interacts with	Baicalein (interacts with) CYP1A2	
**Cytochrome P450 19A1**	452.55159634	interacts with	Baicalein (interacts with) CYP19A1	FALSE	interacts with	Baicalein (interacts with) CYP19A1	
**ATP-binding cassette sub-family G member 2**	437.7031469	interacts with	Baicalein (interacts with) ABCG2	FALSE	interacts with	Baicalein (interacts with) ABCG2	
**Adenosine receptors; A1 & A2a**	1297.0148031	interacts with	Baicalein (interacts with) ADORA1	FALSE	interacts with	Baicalein (interacts with) ADORA1	
**Tyrosine-protein kinase FYN**	554.0	interacts with	Baicalein (interacts with) FYN	FALSE	interacts with	Baicalein (interacts with) FYN	
**Phospholipase A2 group IIA**	554.0	interacts with	Baicalein (interacts with) PLA2G2A	FALSE	interacts with	Baicalein (interacts with) PLA2G2A	
**Carbonic anhydrase XII**	1470.19366672	interacts with	Baicalein (interacts with) CA12	FALSE	interacts with	Baicalein (interacts with) CA12	
**Matrix metalloproteinase 12**	73.09704165	interacts with	1,3-dicaffeoylquinic acid (interacts with) MMP12	FALSE	interacts with	1,3-dicaffeoylquinic acid (interacts with) MMP12	1，3-二咖啡酰奎宁酸
**Aldose reductase**	349.27549057	interacts with	1,3-dicaffeoylquinic acid (interacts with) AKR1B1	FALSE	interacts with	1,3-dicaffeoylquinic acid (interacts with) AKR1B1	
**Matrix metalloproteinase-2**	106.95939444	interacts with	1,3-dicaffeoylquinic acid (interacts with) MMP2	FALSE	interacts with	1,3-dicaffeoylquinic acid (interacts with) MMP2	
**Collagenase**	73.09704165	interacts with	1,3-dicaffeoylquinic acid (interacts with) MMP13	FALSE	interacts with	1,3-dicaffeoylquinic acid (interacts with) MMP13	
**Beta-secretase 1**	554.0	interacts with	scoparone (interacts with) BACE1	FALSE	interacts with	scoparone (interacts with) BACE1	滨蒿内酯
**Monoamine oxidase A**	324.72083802	interacts with	scoparone (interacts with) MAOA	FALSE	interacts with	scoparone (interacts with) MAOA	
**Aldehyde dehydrogenase**	599.2515295	interacts with	scoparone (interacts with) ALDH2	FALSE	interacts with	scoparone (interacts with) ALDH2	
**Carbonic anhydrase XIII**	261.13521319	interacts with	scoparone (interacts with) CA13	FALSE	interacts with	scoparone (interacts with) CA13	
**Carbonic anhydrase VII**	186.18850026	interacts with	scoparone (interacts with) CA7	FALSE	interacts with	scoparone (interacts with) CA7	
**Carbonic anhydrase**	581.01737957	interacts with	scoparone (interacts with) CA3	FALSE	interacts with	scoparone (interacts with) CA3	
**Carbonic anhydrase VI**	623.67702015	interacts with	scoparone (interacts with) CA6	FALSE	interacts with	scoparone (interacts with) CA6	
**Photoreceptor-specific nuclear receptor**	554.0	interacts with	scoparone (interacts with) NR2E3	FALSE	interacts with	scoparone (interacts with) NR2E3	
**Toll-like receptor (TLR7/TLR9)**	554.0	interacts with	scoparone (interacts with) TLR9	FALSE	interacts with	scoparone (interacts with) TLR9	
**Progesterone receptor**	554.0	interacts with	scoparone (interacts with) PGR	FALSE	interacts with	scoparone (interacts with) PGR	
**Peroxisome proliferator-activated receptor gamma/Nuclear receptor corepressor**	554.0	interacts with	scoparone (interacts with) NCOR2	FALSE	interacts with	scoparone (interacts with) NCOR2	
**Carbonic anhydrase XII**	675.47149924	interacts with	scoparone (interacts with) CA12	FALSE	interacts with	scoparone (interacts with) CA12	
**Carbonic anhydrase XIV**	210.7793259	interacts with	scoparone (interacts with) CA14	FALSE	interacts with	scoparone (interacts with) CA14	
**Carbonic anhydrases; II & IX**	237.12691945	interacts with	scoparone (interacts with) CA9	FALSE	interacts with	scoparone (interacts with) CA9	
**Carbonic anhydrase I**	210.7793259	interacts with	scoparone (interacts with) CA1	FALSE	interacts with	scoparone (interacts with) CA1	
**Carbonic anhydrase II**	258.23213856	interacts with	scoparone (interacts with) CA2	FALSE	interacts with	scoparone (interacts with) CA2	
**Monoamine oxidase B**	324.72083802	interacts with	scoparone (interacts with) MAOB	FALSE	interacts with	scoparone (interacts with) MAOB	
**Intestinal alkaline phosphatase**	154.53385357	interacts with	wogonin (interacts with) ALPI	FALSE	interacts with	wogonin (interacts with) ALPI	汉黄芩素
**Intestinal alkaline phosphatase**	428.62689071	interacts with	wogonin (interacts with) AKR1B1	FALSE	interacts with	wogonin (interacts with) AKR1B1	
**Transcription factor AP1**	126.19957405	interacts with	oroxylin A (interacts with) JUN	FALSE	interacts with	oroxylin A (interacts with) JUN	千层纸素A
**Tyrosine-protein kinase receptor FLT3**	124.5071133	interacts with	oroxylin A (interacts with) FLT3	FALSE	interacts with	oroxylin A (interacts with) FLT3	
**Cyclic AMP-responsive element-binding protein 1**	126.19957405	interacts with	oroxylin A (interacts with) CREB1	FALSE	interacts with	oroxylin A (interacts with) CREB1	
**Transcription factor Sp1**	554.0	interacts with	oroxylin A (interacts with) SP1	FALSE	interacts with	oroxylin A (interacts with) SP1	
**Nuclear receptor subfamily 0 group B member 1**	129.825297	interacts with	oroxylin A (interacts with) NR0B1	FALSE	interacts with	oroxylin A (interacts with) NR0B1	
**Aldose reductase**	745.33120951	interacts with	oroxylin A (interacts with) AKR1B1	FALSE	interacts with	oroxylin A (interacts with) AKR1B1	
**GABA-A receptor; anion channel**	180.49743466	interacts with	oroxylin A (interacts with) GABRG2	FALSE	interacts with	oroxylin A (interacts with) GABRG2	
**Cytochrome P450 1A1**	124.5071133	interacts with	oroxylin A (interacts with) CYP1A1	FALSE	interacts with	oroxylin A (interacts with) CYP1A1	
**MAP kinase ERK1**	126.19957405	interacts with	oroxylin A (interacts with) MAPK3	FALSE	interacts with	oroxylin A (interacts with) MAPK3	
**Tankyrase-1**	124.5071133	interacts with	oroxylin A (interacts with) TNKS	FALSE	interacts with	oroxylin A (interacts with) TNKS	
**Arachidonate 5-lipoxygenase**	209.27051624	interacts with	oroxylin A (interacts with) ALOX5	FALSE	interacts with	oroxylin A (interacts with) ALOX5	
**FAD-linked sulfhydryl oxidase ALR**	117.65138059	interacts with	oroxylin A (interacts with) GFER	FALSE	interacts with	oroxylin A (interacts with) GFER	
**Epidermal growth factor receptor erbB1**	249.40534628	interacts with	oroxylin A (interacts with) EGFR	FALSE	interacts with	oroxylin A (interacts with) EGFR	
**P-glycoprotein 1**	208.66315116	interacts with	oroxylin A (interacts with) ABCB1	FALSE	interacts with	oroxylin A (interacts with) ABCB1	
**Nitric oxide synthase, inducible**	126.19957405	interacts with	oroxylin A (interacts with) NOS2	FALSE	interacts with	oroxylin A (interacts with) NOS2	
**Insulin-degrading enzyme**	554.0	interacts with	oroxylin A (interacts with) IDE	FALSE	interacts with	oroxylin A (interacts with) IDE	
**Aryl hydrocarbon receptor**	126.19957405	interacts with	oroxylin A (interacts with) AHR	FALSE	interacts with	oroxylin A (interacts with) AHR	
**Sucrase-isomaltase**	172.30237305	interacts with	oroxylin A (interacts with) SI	FALSE	interacts with	oroxylin A (interacts with) SI	
**Tankyrase-2**	124.5071133	interacts with	oroxylin A (interacts with) TNKS2	FALSE	interacts with	oroxylin A (interacts with) TNKS2	
**GABA receptor delta subunit**	126.19957405	interacts with	oroxylin A (interacts with) GABRD	FALSE	interacts with	oroxylin A (interacts with) GABRD	
**ATP-binding cassette sub-family G member 2**	242.65545958	interacts with	oroxylin A (interacts with) ABCG2	FALSE	interacts with	oroxylin A (interacts with) ABCG2	
**Xanthine dehydrogenase**	418.85423707	interacts with	oroxylin A (interacts with) XDH	FALSE	interacts with	oroxylin A (interacts with) XDH	
**Cytochrome P450 19A1**	126.19957405	interacts with	oroxylin A (interacts with) CYP19A1	FALSE	interacts with	oroxylin A (interacts with) CYP19A1	
**Adenosine receptors; A1 & A2a**	362.04564698	interacts with	oroxylin A (interacts with) ADORA1	FALSE	interacts with	oroxylin A (interacts with) ADORA1	
**Adrenergic receptors; alpha-1 A & B**	554.0	interacts with	Hyperoside (interacts with) ADRA1B	FALSE	interacts with	Hyperoside (interacts with) ADRA1B	金丝桃苷
**Alpha adrenergic receptor (1a and 1d)**	554.0	interacts with	Hyperoside (interacts with) ADRA1A	FALSE	interacts with	Hyperoside (interacts with) ADRA1A	
**Alpha-2a adrenergic receptor**	554.0	interacts with	Hyperoside (interacts with) ADRA2A	FALSE	interacts with	Hyperoside (interacts with) ADRA2A	
**Alpha-2b adrenergic receptor**	554.0	interacts with	Hyperoside (interacts with) ADRA2B	FALSE	interacts with	Hyperoside (interacts with) ADRA2B	
**Dopamine D1 receptor**	554.0	interacts with	Hyperoside (interacts with) DRD1	FALSE	interacts with	Hyperoside (interacts with) DRD1	
**Dopamine D5 receptor**	554.0	interacts with	Hyperoside (interacts with) DRD5	FALSE	interacts with	Hyperoside (interacts with) DRD5	

**Kappa opioid receptor**	554.0	interacts with	Hyperoside (interacts with) OPRK1	FALSE	interacts with	Hyperoside (interacts with) OPRK1	
**Lysosomal alpha-glucosidase**	475.71800779	interacts with	Hyperoside (interacts with) GAA	FALSE	interacts with	Hyperoside (interacts with) GAA	
**Mannose-6-phosphate isomerase**	554.0	interacts with	Hyperoside (interacts with) MPI	FALSE	interacts with	Hyperoside (interacts with) MPI	
**Muscarinic acetylcholine receptor**	554.0	interacts with	Hyperoside (interacts with) CHRM3	FALSE	interacts with	Hyperoside (interacts with) CHRM3	
**Muscarinic acetylcholine receptor M2**	554.0	interacts with	Hyperoside (interacts with) CHRM2	FALSE	interacts with	Hyperoside (interacts with) CHRM2	
**Muscarinic acetylcholine receptor M4**	554.0	interacts with	Hyperoside (interacts with) CHRM4	FALSE	interacts with	Hyperoside (interacts with) CHRM4	
**Muscarinic acetylcholine receptors; M1 & M2**	554.0	interacts with	Hyperoside (interacts with) CHRM1	FALSE	interacts with	Hyperoside (interacts with) CHRM1	
**Opioid receptors; mu & delta**	554.0	interacts with	Hyperoside (interacts with) OPRM1	FALSE	interacts with	Hyperoside (interacts with) OPRM1	
**Serotonin 1a (5-HT1a) receptor**	554.0	interacts with	Hyperoside (interacts with) HTR1A	FALSE	interacts with	Hyperoside (interacts with) HTR1A	
**Serotonin 1d (5-HT1d) recept**	554.0	interacts with	Hyperoside (interacts with) HTR1D	FALSE	interacts with	Hyperoside (interacts with) HTR1D	
**Serotonin 3a (5-HT3a) receptor**	554.0	interacts with	Hyperoside (interacts with) HTR3A	FALSE	interacts with	Hyperoside (interacts with) HTR3A	
**Serotonin 6 (5-HT6) receptor**	554.0	interacts with	Hyperoside (interacts with) HTR6	FALSE	interacts with	Hyperoside (interacts with) HTR6	
**Serotonin and norepinephrine transporters (SERT/NET)**	554.0	interacts with	Hyperoside (interacts with) SLC6A2	FALSE	interacts with	Hyperoside (interacts with) SLC6A2	
**Serotonin transporter**	554.0	interacts with	Hyperoside (interacts with) SLC6A4	FALSE	interacts with	Hyperoside (interacts with) SLC6A4	
**Vasopressin V1b receptor**	554.0	interacts with	Hyperoside (interacts with) AVPR1B	FALSE	interacts with	Hyperoside (interacts with) AVPR1B	
**Vasopressin V2 receptor**	554.0	interacts with	Hyperoside (interacts with) AVPR2	FALSE	interacts with	Hyperoside (interacts with) AVPR2	
**Alpha-2c adrenergic receptor**	554.0	interacts with	Hyperoside (interacts with) ADRA2C	FALSE	interacts with	Hyperoside (interacts with) ADRA2C	
**DNA polymerase eta**	365.95763324	interacts with	Hyperoside (interacts with) POLH	FALSE	interacts with	Hyperoside (interacts with) POLH	
**DNA polymerase iota**	365.95763324	interacts with	Hyperoside (interacts with) POLI	FALSE	interacts with	Hyperoside (interacts with) POLI	
**DNA polymerase kappa**	475.71800779	interacts with	Hyperoside (interacts with) POLK	FALSE	interacts with	Hyperoside (interacts with) POLK	
**DNA-(apurinic or apyrimidinic site) lyase**	475.71800779	interacts with	Hyperoside (interacts with) APEX1	FALSE	interacts with	Hyperoside (interacts with) APEX1	
**Flap endonuclease 1**	365.95763324	interacts with	Hyperoside (interacts with) FEN1	FALSE	interacts with	Hyperoside (interacts with) FEN1	
**Geminin**	466.48136425	interacts with	Hyperoside (interacts with) GMNN	FALSE	interacts with	Hyperoside (interacts with) GMNN	
**Glutaminase kidney isoform, mitochondrial**	479.2963226	interacts with	Hyperoside (interacts with) GLS	FALSE	interacts with	Hyperoside (interacts with) GLS	
**Lysine-specific demethylase 4D-like**	475.71800779	interacts with	Hyperoside (interacts with) KDM4E	FALSE	interacts with	Hyperoside (interacts with) KDM4E	
**Muscleblind-like protein 1**	277.23340857	interacts with	Hyperoside (interacts with) MBNL1	FALSE	interacts with	Hyperoside (interacts with) MBNL1	
**Protein disulfide-isomerase**	391.40478376	interacts with	Hyperoside (interacts with) P4HB	FALSE	interacts with	Hyperoside (interacts with) P4HB	
**Runt-related transcription factor 1/Core-binding factor subunit beta**	365.95763324	interacts with	Hyperoside (interacts with) RUNX1	FALSE	interacts with	Hyperoside (interacts with) RUNX1	
**Serotonin (5-HT3) receptor**	539.41152298	interacts with	Hyperoside (interacts with) HTR7	FALSE	interacts with	Hyperoside (interacts with) HTR7	
**Survival motor neuron protein**	365.95763324	interacts with	Hyperoside (interacts with) SMN1	FALSE	interacts with	Hyperoside (interacts with) SMN1	
**Protein disulfide-isomerase**	391.40478376	interacts with	Hyperoside (interacts with) P4HB	FALSE	interacts with	Hyperoside (interacts with) P4HB	
**Ribosomal protein S6 kinase alpha 3**	740.83364013	interacts with	Hyperoside (interacts with) RPS6KA3	FALSE	interacts with	Hyperoside (interacts with) RPS6KA3	
**Lysine-specific demethylase 4A**	604.97286406	interacts with	Hyperoside (interacts with) KDM4A	FALSE	interacts with	Hyperoside (interacts with) KDM4A	
**PI3-kinase p85-alpha subunit**	277.23340857	interacts with	Hyperoside (interacts with) PIK3R1	FALSE	interacts with	Hyperoside (interacts with) PIK3R1	
**Aldose reductase**	1609.61691401	interacts with	Hyperoside (interacts with) AKR1B1	FALSE	interacts with	Hyperoside (interacts with) AKR1B1	
**Intestinal alkaline phosphatase**	601.97656635	interacts with	Hyperoside (interacts with) ALPI	FALSE	interacts with	Hyperoside (interacts with) ALPI	
**Dual-specificity tyrosine-phosphorylation regulated kinase 1A**	359.28558343	interacts with	Hyperoside (interacts with) DYRK1A	FALSE	interacts with	Hyperoside (interacts with) DYRK1A	
**Lysine-specific demethylase 4C**	669.39245273	interacts with	Hyperoside (interacts with) KDM4C	FALSE	interacts with	Hyperoside (interacts with) KDM4C	
**Xanthine dehydrogenase**	1017.5263694	interacts with	Hyperoside (interacts with) XDH	FALSE	interacts with	Hyperoside (interacts with) XDH	
**Tyrosinase**	672.57386259	interacts with	Hyperoside (interacts with) TYR	FALSE	interacts with	Hyperoside (interacts with) TYR	
**Aldehyde reductase**	277.23340857	interacts with	Hyperoside (interacts with) AKR1A1	FALSE	interacts with	Hyperoside (interacts with) AKR1A1	
**Sentrin-specific protease 1**	268.47465495	interacts with	Hyperoside (interacts with) SENP1	FALSE	interacts with	Hyperoside (interacts with) SENP1	
**Carbonic anhydrase V**	738.58426905	interacts with	Hyperoside (interacts with) CA5A	FALSE	interacts with	Hyperoside (interacts with) CA5A	
**5'-nucleotidase**	277.23340857	interacts with	Hyperoside (interacts with) NT5E	FALSE	interacts with	Hyperoside (interacts with) NT5E	
**CaM kinase II**	277.23340857	interacts with	Hyperoside (interacts with) CAMK2B	FALSE	interacts with	Hyperoside (interacts with) CAMK2B	
**Carbonic anhydrase**	761.76453417	interacts with	Hyperoside (interacts with) CA3	FALSE	interacts with	Hyperoside (interacts with) CA3	
**NADPH oxidase 4**	277.23340857	interacts with	Hyperoside (interacts with) NOX4	FALSE	interacts with	Hyperoside (interacts with) NOX4	
**Carbonic anhydrase VI**	950.24907657	interacts with	Hyperoside (interacts with) CA6	FALSE	interacts with	Hyperoside (interacts with) CA6	
**Cytochrome P450 1B1**	365.95763324	interacts with	Hyperoside (interacts with) CYP1B1	FALSE	interacts with	Hyperoside (interacts with) CYP1B1	
**Arachidonate 12-lipoxygenase**	365.95763324	interacts with	Hyperoside (interacts with) ALOX12	FALSE	interacts with	Hyperoside (interacts with) ALOX12	
**Aldose reductase**	1533.52731541	interacts with	Caffeic acid (interacts with) AKR1B1	FALSE	interacts with	Caffeic acid (interacts with) AKR1B1	咖啡酸
**Alpha-galactosidase A**	307.03222572	interacts with	Caffeic acid (interacts with) GLA	FALSE	interacts with	Caffeic acid (interacts with) GLA	
**Arachidonate 5-lipoxygenase**	482.84189192	interacts with	Caffeic acid (interacts with) ALOX5	FALSE	interacts with	Caffeic acid (interacts with) ALOX5	
**Carbonic anhydrase II**	559.24437216	interacts with	Caffeic acid (interacts with) CA2	FALSE	interacts with	Caffeic acid (interacts with) CA2	
**Cyclooxygenase**	307.03222572	interacts with	Caffeic acid (interacts with) PTGS1	FALSE	interacts with	Caffeic acid (interacts with) PTGS1	
**Cyclooxygenase-2**	281.15837557	interacts with	Caffeic acid (interacts with) PTGS2	FALSE	interacts with	Caffeic acid (interacts with) PTGS2	
**Cytochrome P450 3A4**	384.52484601	interacts with	Caffeic acid (interacts with) CYP3A4	FALSE	interacts with	Caffeic acid (interacts with) CYP3A4	
**Epidermal growth factor receptor erbB1**	1017.23041699	interacts with	Caffeic acid (interacts with) EGFR	FALSE	interacts with	Caffeic acid (interacts with) EGFR	
**Estrogen receptor alpha**	810.94292043	interacts with	Caffeic acid (interacts with) ESR1	FALSE	interacts with	Caffeic acid (interacts with) ESR1	
**Heat shock protein HSP90**	554.0	interacts with	Caffeic acid (interacts with) HSP90AA1	FALSE	interacts with	Caffeic acid (interacts with) HSP90AA1	
**HERG**	554.0	interacts with	Caffeic acid (interacts with) KCNH2	FALSE	interacts with	Caffeic acid (interacts with) KCNH2	
**Hydroxycarboxylic acid receptor 2**	554.0	interacts with	Caffeic acid (interacts with) HCAR2	FALSE	interacts with	Caffeic acid (interacts with) HCAR2	
**LDL-associated phospholipase A2**	360.05399977	interacts with	Caffeic acid (interacts with) PLA2G7	FALSE	interacts with	Caffeic acid (interacts with) PLA2G7	
**Lysosomal alpha-glucosidase**	499.33221741	interacts with	Caffeic acid (interacts with) GAA	FALSE	interacts with	Caffeic acid (interacts with) GAA	
**Matrix metalloproteinase (1 and 13)**	279.54424393	interacts with	Caffeic acid (interacts with) MMP1	FALSE	interacts with	Caffeic acid (interacts with) MMP1	
**Matrix metalloproteinase 9**	279.54424393	interacts with	Caffeic acid (interacts with) MMP9	FALSE	interacts with	Caffeic acid (interacts with) MMP9	
**Matrix metalloproteinase-2**	373.90726168	interacts with	Caffeic acid (interacts with) MMP2	FALSE	interacts with	Caffeic acid (interacts with) MMP2	
**Protein-tyrosine phosphatase 1B**	321.55584737	interacts with	Caffeic acid (interacts with) PTPN1	FALSE	interacts with	Caffeic acid (interacts with) PTPN1	
**Tyrosinase**	485.56647358	interacts with	Caffeic acid (interacts with) TYR	FALSE	interacts with	Caffeic acid (interacts with) TYR	
**Xanthine dehydrogenase**	1194.76445916	interacts with	Caffeic acid (interacts with) XDH	FALSE	interacts with	Caffeic acid (interacts with) XDH	
**15-hydroxyprostaglandin dehydrogenase [NAD+]**	384.52484601	interacts with	Caffeic acid (interacts with) HPGD	FALSE	interacts with	Caffeic acid (interacts with) HPGD	
**Alanine aminotransferase 1**	554.0	interacts with	Caffeic acid (interacts with) GPT	FALSE	interacts with	Caffeic acid (interacts with) GPT	
**Aldehyde dehydrogenase 1A1**	384.52484601	interacts with	Caffeic acid (interacts with) ALDH1A1	FALSE	interacts with	Caffeic acid (interacts with) ALDH1A1	
**Aldo-keto reductase family 1 member B10**	352.00107965	interacts with	Caffeic acid (interacts with) AKR1B10	FALSE	interacts with	Caffeic acid (interacts with) AKR1B10	
**Aldo-keto reductase family 1 member C1**	554.0	interacts with	Caffeic acid (interacts with) AKR1C1	FALSE	interacts with	Caffeic acid (interacts with) AKR1C1	
**Aldo-keto reductase family 1 member C2**	507.04922946	interacts with	Caffeic acid (interacts with) AKR1C2	FALSE	interacts with	Caffeic acid (interacts with) AKR1C2	
**Aldo-keto reductase family 1 member C4**	554.0	interacts with	Caffeic acid (interacts with) AKR1C4	FALSE	interacts with	Caffeic acid (interacts with) AKR1C4	
**Aldo-keto-reductase family 1 member C3**	507.04922946	interacts with	Caffeic acid (interacts with) AKR1C3	FALSE	interacts with	Caffeic acid (interacts with) AKR1C3	
**Bromodomain adjacent to zinc finger domain protein 2B**	307.03222572	interacts with	Caffeic acid (interacts with) BAZ2B	FALSE	interacts with	Caffeic acid (interacts with) BAZ2B	
**Carbonic anhydrase**	498.74066233	interacts with	Caffeic acid (interacts with) CA3	FALSE	interacts with	Caffeic acid (interacts with) CA3	
**Carbonic anhydrase I**	475.7150292	interacts with	Caffeic acid (interacts with) CA1	FALSE	interacts with	Caffeic acid (interacts with) CA1	
**Carbonic anhydrase IV**	554.0	interacts with	Caffeic acid (interacts with) CA4	FALSE	interacts with	Caffeic acid (interacts with) CA4	
**Carbonic anhydrase V**	446.78227983	interacts with	Caffeic acid (interacts with) CA5A	FALSE	interacts with	Caffeic acid (interacts with) CA5A	
**Carbonic anhydrase VB**	321.55584737	interacts with	Caffeic acid (interacts with) CA5B	FALSE	interacts with	Caffeic acid (interacts with) CA5B	
**Carbonic anhydrase VI**	515.55194813	interacts with	Caffeic acid (interacts with) CA6	FALSE	interacts with	Caffeic acid (interacts with) CA6	
**Carbonic anhydrase VII**	413.64748393	interacts with	Caffeic acid (interacts with) CA7	FALSE	interacts with	Caffeic acid (interacts with) CA7	
**Carbonic anhydrase XII**	529.11959264	interacts with	Caffeic acid (interacts with) CA12	FALSE	interacts with	Caffeic acid (interacts with) CA12	
**Carbonic anhydrase XIV**	475.7150292	interacts with	Caffeic acid (interacts with) CA14	FALSE	interacts with	Caffeic acid (interacts with) CA14	
**Carbonic anhydrases; II & IX**	479.71500491	interacts with	Caffeic acid (interacts with) CA9	FALSE	interacts with	Caffeic acid (interacts with) CA9	
**DNA polymerase beta**	307.03222572	interacts with	Caffeic acid (interacts with) POLB	FALSE	interacts with	Caffeic acid (interacts with) POLB	
**DNA polymerase kappa**	499.33221741	interacts with	Caffeic acid (interacts with) POLK	FALSE	interacts with	Caffeic acid (interacts with) POLK	
**DNA-(apurinic or apyrimidinic site) lyase**	499.33221741	interacts with	Caffeic acid (interacts with) APEX1	FALSE	interacts with	Caffeic acid (interacts with) APEX1	
**Dual specificity protein phosphatase 3**	554.0	interacts with	Caffeic acid (interacts with) DUSP3	FALSE	interacts with	Caffeic acid (interacts with) DUSP3	
**Egl nine homolog 1**	376.2277707	interacts with	Caffeic acid (interacts with) EGLN1	FALSE	interacts with	Caffeic acid (interacts with) EGLN1	
**Endoplasmic reticulum-associated amyloid beta-peptide-binding protein**	384.52484601	interacts with	Caffeic acid (interacts with) HSD17B10	FALSE	interacts with	Caffeic acid (interacts with) HSD17B10	
**Glutaminase kidney isoform, mitochondrial**	393.47075181	interacts with	Caffeic acid (interacts with) GLS	FALSE	interacts with	Caffeic acid (interacts with) GLS	
**Histone-lysine N-methyltransferase MLL**	384.52484601	interacts with	Caffeic acid (interacts with) KMT2A	FALSE	interacts with	Caffeic acid (interacts with) KMT2A	
**Histone-lysine N-methyltransferase, H3 lysine-9 specific 3**	281.15837557	interacts with	Caffeic acid (interacts with) EHMT2	FALSE	interacts with	Caffeic acid (interacts with) EHMT2	
**Hyaluronidase-1**	554.0	interacts with	Caffeic acid (interacts with) HYAL1	FALSE	interacts with	Caffeic acid (interacts with) HYAL1	
**Lysine-specific demethylase 4A**	521.11522996	interacts with	Caffeic acid (interacts with) KDM4A	FALSE	interacts with	Caffeic acid (interacts with) KDM4A	
**Lysine-specific demethylase 4D-like**	499.33221741	interacts with	Caffeic acid (interacts with) KDM4E	FALSE	interacts with	Caffeic acid (interacts with) KDM4E	
**Microtubule-associated protein tau**	384.52484601	interacts with	Caffeic acid (interacts with) MAPT	FALSE	interacts with	Caffeic acid (interacts with) MAPT	
**Mitogen-activated protein kinase; ERK1/ERK2**	554.0	interacts with	Caffeic acid (interacts with) MAPK1	FALSE	interacts with	Caffeic acid (interacts with) MAPK1	
**Nuclear factor NF-kappa-B p105 subunit**	554.0	interacts with	Caffeic acid (interacts with) NFKB1	FALSE	interacts with	Caffeic acid (interacts with) NFKB1	
**Peripheral myelin protein 22**	554.0	interacts with	Caffeic acid (interacts with) PMP22	FALSE	interacts with	Caffeic acid (interacts with) PMP22	
**Prelamin-A/C**	307.03222572	interacts with	Caffeic acid (interacts with) LMNA	FALSE	interacts with	Caffeic acid (interacts with) LMNA	
**Protein-tyrosine phosphatase 2C**	554.0	interacts with	Caffeic acid (interacts with) PTPN11	FALSE	interacts with	Caffeic acid (interacts with) PTPN11	
**Protein-tyrosine phosphatase LC-PTP**	554.0	interacts with	Caffeic acid (interacts with) PTPN7	FALSE	interacts with	Caffeic acid (interacts with) PTPN7	
**Thyroid hormone receptor beta-1**	384.52484601	interacts with	Caffeic acid (interacts with) THRB	FALSE	interacts with	Caffeic acid (interacts with) THRB	
**Tyrosyl-DNA phosphodiesterase 1**	307.03222572	interacts with	Caffeic acid (interacts with) TDP1	FALSE	interacts with	Caffeic acid (interacts with) TDP1	
**Carbonic anhydrase V**	446.78227983	interacts with	Caffeic acid (interacts with) CA5A	FALSE	interacts with	Caffeic acid (interacts with) CA5A	
**Carbonic anhydrase VI**	515.55194813	interacts with	Caffeic acid (interacts with) CA6	FALSE	interacts with	Caffeic acid (interacts with) CA6	
**Carbonic anhydrase VB**	321.55584737	interacts with	Caffeic acid (interacts with) CA5B	FALSE	interacts with	Caffeic acid (interacts with) CA5B	
**Arachidonate 5-lipoxygenase**	482.84189192	interacts with	Caffeic acid (interacts with) ALOX5	FALSE	interacts with	Caffeic acid (interacts with) ALOX5	
**Carbonic anhydrase VII**	413.64748393	interacts with	Caffeic acid (interacts with) CA7	FALSE	interacts with	Caffeic acid (interacts with) CA7	

**MAP/microtubule affinity-regulating kinase 4**	554.0	interacts with	Caffeic acid (interacts with) MARK4	FALSE	interacts with	Caffeic acid (interacts with) MARK4	
**Receptor-type tyrosine-protein phosphatase beta**	554.0	interacts with	Caffeic acid (interacts with) PTPRB	FALSE	interacts with	Caffeic acid (interacts with) PTPRB	
**CaM-kinase kinase beta**	554.0	interacts with	Caffeic acid (interacts with) CAMKK2	FALSE	interacts with	Caffeic acid (interacts with) CAMKK2	
**Beta amyloid A4 protein**	554.0	interacts with	Caffeic acid (interacts with) APP	FALSE	interacts with	Caffeic acid (interacts with) APP	
**Carbonic anhydrase XIV**	475.7150292	interacts with	Caffeic acid (interacts with) CA14	FALSE	interacts with	Caffeic acid (interacts with) CA14	
**Mitogen-activated protein kinase kinase kinase 10**	554.0	interacts with	Caffeic acid (interacts with) MAP3K10	FALSE	interacts with	Caffeic acid (interacts with) MAP3K10	
**Carbonic anhydrase XII**	529.11959264	interacts with	Caffeic acid (interacts with) CA12	FALSE	interacts with	Caffeic acid (interacts with) CA12	
**Carbonic anhydrase**	498.74066233	interacts with	Caffeic acid (interacts with) CA3	FALSE	interacts with	Caffeic acid (interacts with) CA3	
**Dual-specificity tyrosine-phosphorylation regulated kinase 1A**	434.86558978	interacts with	Caffeic acid (interacts with) DYRK1A	FALSE	interacts with	Caffeic acid (interacts with) DYRK1A	
**SUMO-activating enzyme**	554.0	interacts with	Caffeic acid (interacts with) UBA2	FALSE	interacts with	Caffeic acid (interacts with) UBA2	
**DNA-(apurinic or apyrimidinic site) lyase**	499.33221741	interacts with	Caffeic acid (interacts with) APEX1	FALSE	interacts with	Caffeic acid (interacts with) APEX1	
**Carbonic anhydrases; II & IX**	479.71500491	interacts with	Caffeic acid (interacts with) CA9	FALSE	interacts with	Caffeic acid (interacts with) CA9	
**Protein-tyrosine phosphatase 1B**	321.55584737	interacts with	Caffeic acid (interacts with) PTPN1	FALSE	interacts with	Caffeic acid (interacts with) PTPN1	
**Carbonic anhydrase I**	475.7150292	interacts with	Caffeic acid (interacts with) CA1	FALSE	interacts with	Caffeic acid (interacts with) CA1	
**Carbonic anhydrase II**	559.24437216	interacts with	Caffeic acid (interacts with) CA2	FALSE	interacts with	Caffeic acid (interacts with) CA2	
**Adenosine A1 receptor (by homology)**	916.73971437	interacts with	geniposidic acid (interacts with) ADORA1	FALSE	interacts with	geniposidic acid (interacts with) ADORA1	栀子酸
**P-selectin**	392.23395939	interacts with	geniposidic acid (interacts with) SELP	FALSE	interacts with	geniposidic acid (interacts with) SELP	
**Endo-beta-N-acetylglucosaminidase**	554.0	interacts with	geniposidic acid (interacts with) ENGASE	FALSE	interacts with	geniposidic acid (interacts with) ENGASE	
**Tyrosyl-tRNA synthetase**	554.0	interacts with	geniposidic acid (interacts with) YARS	FALSE	interacts with	geniposidic acid (interacts with) YARS	
**Leukocyte adhesion molecule-1**	392.23395939	interacts with	geniposidic acid (interacts with) SELL	FALSE	interacts with	geniposidic acid (interacts with) SELL	
**Lysine-specific demethylase 4A**	191.98974835	interacts with	luteoloside (interacts with) KDM4A	FALSE	interacts with	luteoloside (interacts with) KDM4A	木犀草苷
**Protein disulfide-isomerase**	203.25546328	interacts with	luteoloside (interacts with) P4HB	FALSE	interacts with	luteoloside (interacts with) P4HB	
**Lysine-specific demethylase 4C**	170.64852514	interacts with	luteoloside (interacts with) KDM4C	FALSE	interacts with	luteoloside (interacts with) KDM4C	
**Sentrin-specific protease 1**	140.40022436	interacts with	luteoloside (interacts with) SENP1	FALSE	interacts with	luteoloside (interacts with) SENP1	
**Aldose reductase**	334.45351925	interacts with	luteoloside (interacts with) AKR1B1	FALSE	interacts with	luteoloside (interacts with) AKR1B1	
**Tyrosinase**	159.2579013	interacts with	luteoloside (interacts with) TYR	FALSE	interacts with	luteoloside (interacts with) TYR	
**Intestinal alkaline phosphatase**	224.22990043	interacts with	luteoloside (interacts with) ALPI	FALSE	interacts with	luteoloside (interacts with) ALPI	
**Dual-specificity tyrosine-phosphorylation regulated kinase 1A**	139.30420793	interacts with	luteoloside (interacts with) DYRK1A	FALSE	interacts with	luteoloside (interacts with) DYRK1A	
**Aldehyde dehydrogenase**	307.2139511	interacts with	luteoloside (interacts with) ALDH2	FALSE	interacts with	luteoloside (interacts with) ALDH2	
**Carbonic anhydrase XII**	260.37740848	interacts with	luteoloside (interacts with) CA12	FALSE	interacts with	luteoloside (interacts with) CA12	
**Ribosomal protein S6 kinase alpha 3**	198.90561588	interacts with	luteoloside (interacts with) RPS6KA3	FALSE	interacts with	luteoloside (interacts with) RPS6KA3	
**Xanthine dehydrogenase**	226.51112915	interacts with	luteoloside (interacts with) XDH	FALSE	interacts with	luteoloside (interacts with) XDH	
**Carbonic anhydrases; II & IX**	251.98535416	interacts with	luteoloside (interacts with) CA9	FALSE	interacts with	luteoloside (interacts with) CA9	
**FAD-linked sulfhydryl oxidase ALR**	183.42328096	interacts with	luteoloside (interacts with) GFER	FALSE	interacts with	luteoloside (interacts with) GFER	
**Neurotensin receptor**	438.81381173	interacts with	luteoloside (interacts with) NTSR1	FALSE	interacts with	luteoloside (interacts with) NTSR1	
**Arachidonate 5-lipoxygenase**	174.99839361	interacts with	luteoloside (interacts with) ALOX5	FALSE	interacts with	luteoloside (interacts with) ALOX5	
**Alpha-galactosidase A**	450.15166178	interacts with	luteolin (interacts with) GLA	FALSE	interacts with	luteolin (interacts with) GLA	木犀草素
**Androgen Receptor**	383.5908713	interacts with	luteolin (interacts with) AR	FALSE	interacts with	luteolin (interacts with) AR	
**Beta-glucocerebrosidase**	383.5908713	interacts with	luteolin (interacts with) GBA	FALSE	interacts with	luteolin (interacts with) GBA	
**Butyrylcholinesterase**	554.0	interacts with	luteolin (interacts with) BCHE	FALSE	interacts with	luteolin (interacts with) BCHE	
**CDK6/cyclin D3**	554.0	interacts with	luteolin (interacts with) CDK6	FALSE	interacts with	luteolin (interacts with) CDK6	
**Cholinesterases; ACHE & BCHE**	554.0	interacts with	luteolin (interacts with) ACHE	FALSE	interacts with	luteolin (interacts with) ACHE	
**Cyclin-dependent kinase 2**	554.0	interacts with	luteolin (interacts with) CDK2	FALSE	interacts with	luteolin (interacts with) CDK2	
**Cyclooxygenase**	450.15166178	interacts with	luteolin (interacts with) PTGS1	FALSE	interacts with	luteolin (interacts with) PTGS1	
**Cyclooxygenase-2**	507.42398964	interacts with	luteolin (interacts with) PTGS2	FALSE	interacts with	luteolin (interacts with) PTGS2	
**Cytochrome P450 2D6**	383.5908713	interacts with	luteolin (interacts with) CYP2D6	FALSE	interacts with	luteolin (interacts with) CYP2D6	
**Cytochrome P450 3A4**	383.00761593	interacts with	luteolin (interacts with) CYP3A4	FALSE	interacts with	luteolin (interacts with) CYP3A4	
**DNA topoisomerase I**	554.0	interacts with	luteolin (interacts with) TOP1	FALSE	interacts with	luteolin (interacts with) TOP1	
**DNA topoisomerase II alpha**	554.0	interacts with	luteolin (interacts with) TOP2A	FALSE	interacts with	luteolin (interacts with) TOP2A	
**Estrogen receptor alpha**	840.10846439	interacts with	luteolin (interacts with) ESR1	FALSE	interacts with	luteolin (interacts with) ESR1	
**Fatty acid synthase**	554.0	interacts with	luteolin (interacts with) FASN	FALSE	interacts with	luteolin (interacts with) FASN	
**Glucocorticoid receptor**	383.5908713	interacts with	luteolin (interacts with) NR3C1	FALSE	interacts with	luteolin (interacts with) NR3C1	
**Glycogen synthase kinase-3**	554.0	interacts with	luteolin (interacts with) GSK3B	FALSE	interacts with	luteolin (interacts with) GSK3B	
**Hypoxia-inducible factor 1 alpha**	554.0	interacts with	luteolin (interacts with) HIF1A	FALSE	interacts with	luteolin (interacts with) HIF1A	
**Lysosomal alpha-glucosidase**	442.13683344	interacts with	luteolin (interacts with) GAA	FALSE	interacts with	luteolin (interacts with) GAA	
**MAP kinase p38 alpha**	1020.09357582	interacts with	luteolin (interacts with) MAPK14	FALSE	interacts with	luteolin (interacts with) MAPK14	
**Matrix metalloproteinase (1 and 13)**	549.99140204	interacts with	luteolin (interacts with) MMP1	FALSE	interacts with	luteolin (interacts with) MMP1	
**Matrix metalloproteinase (2 and 3)**	554.0	interacts with	luteolin (interacts with) MMP3	FALSE	interacts with	luteolin (interacts with) MMP3	
**Matrix metalloproteinase 12**	628.35142631	interacts with	luteolin (interacts with) MMP12	FALSE	interacts with	luteolin (interacts with) MMP12	
**Matrix metalloproteinase 9**	549.99140204	interacts with	luteolin (interacts with) MMP9	FALSE	interacts with	luteolin (interacts with) MMP9	
**Matrix metalloproteinase-2**	665.95085146	interacts with	luteolin (interacts with) MMP2	FALSE	interacts with	luteolin (interacts with) MMP2	
**Monoamine oxidase A**	711.43248303	interacts with	luteolin (interacts with) MAOA	FALSE	interacts with	luteolin (interacts with) MAOA	
**Monoamine oxidase B**	711.43248303	interacts with	luteolin (interacts with) MAOB	FALSE	interacts with	luteolin (interacts with) MAOB	
**Poly [ADP-ribose] polymerase-1**	554.0	interacts with	luteolin (interacts with) PARP1	FALSE	interacts with	luteolin (interacts with) PARP1	
**Thyroid stimulating hormone receptor**	554.0	interacts with	luteolin (interacts with) TSHR	FALSE	interacts with	luteolin (interacts with) TSHR	
**Tumor necrosis factor receptor superfamily member 10B**	554.0	interacts with	luteolin (interacts with) TNFRSF10B	FALSE	interacts with	luteolin (interacts with) TNFRSF10B	
**Tumour suppressor p53/oncoprotein Mdm2**	383.5908713	interacts with	luteolin (interacts with) TP53	FALSE	interacts with	luteolin (interacts with) TP53	
**Tyrosinase**	767.87116915	interacts with	luteolin (interacts with) TYR	FALSE	interacts with	luteolin (interacts with) TYR	
**Tyrosine-protein kinase LCK**	493.01468866	interacts with	luteolin (interacts with) LCK	FALSE	interacts with	luteolin (interacts with) LCK	
**Tyrosine-protein kinase receptor FLT3**	413.21872306	interacts with	luteolin (interacts with) FLT3	FALSE	interacts with	luteolin (interacts with) FLT3	
**Xanthine dehydrogenase**	1083.45359247	interacts with	luteolin (interacts with) XDH	FALSE	interacts with	luteolin (interacts with) XDH	
**15-hydroxyprostaglandin dehydrogenase [NAD+]**	383.00761593	interacts with	luteolin (interacts with) HPGD	FALSE	interacts with	luteolin (interacts with) HPGD	
**78 kDa glucose-regulated protein**	554.0	interacts with	luteolin (interacts with) HSPA5	FALSE	interacts with	luteolin (interacts with) HSPA5	
**Aldehyde dehydrogenase 1A1**	383.00761593	interacts with	luteolin (interacts with) ALDH1A1	FALSE	interacts with	luteolin (interacts with) ALDH1A1	
**Arachidonate 12-lipoxygenase**	409.8200966	interacts with	luteolin (interacts with) ALOX12	FALSE	interacts with	luteolin (interacts with) ALOX12	
**Arachidonate 15-lipoxygenase**	383.5908713	interacts with	luteolin (interacts with) ALOX15	FALSE	interacts with	luteolin (interacts with) ALOX15	
**Arachidonate 15-lipoxygenase, type II**	383.5908713	interacts with	luteolin (interacts with) ALOX15B	FALSE	interacts with	luteolin (interacts with) ALOX15B	
**Ataxin-2**	554.0	interacts with	luteolin (interacts with) ATXN2	FALSE	interacts with	luteolin (interacts with) ATXN2	
**ATP-binding cassette sub-family G member 2**	586.77944647	interacts with	luteolin (interacts with) ABCG2	FALSE	interacts with	luteolin (interacts with) ABCG2	
**ATP-dependent DNA helicase Q1**	383.5908713	interacts with	luteolin (interacts with) RECQL	FALSE	interacts with	luteolin (interacts with) RECQL	
**ATPase family AAA domain-containing protein 5**	383.5908713	interacts with	luteolin (interacts with) ATAD5	FALSE	interacts with	luteolin (interacts with) ATAD5	
**Bloom syndrome protein**	383.5908713	interacts with	luteolin (interacts with) BLM	FALSE	interacts with	luteolin (interacts with) BLM	
**Breast cancer type 1 susceptibility protein**	383.5908713	interacts with	luteolin (interacts with) BRCA1	FALSE	interacts with	luteolin (interacts with) BRCA1	
**Bromodomain adjacent to zinc finger domain protein 2B**	450.15166178	interacts with	luteolin (interacts with) BAZ2B	FALSE	interacts with	luteolin (interacts with) BAZ2B	
**c-Jun N-terminal kinase 3**	554.0	interacts with	luteolin (interacts with) MAPK10	FALSE	interacts with	luteolin (interacts with) MAPK10	
**Collagenase**	628.35142631	interacts with	luteolin (interacts with) MMP13	FALSE	interacts with	luteolin (interacts with) MMP13	
**Cyclin-dependent kinase 1/cyclin B**	554.0	interacts with	luteolin (interacts with) CCNB2	FALSE	interacts with	luteolin (interacts with) CCNB2	
**Cyclin-dependent kinase 5/CDK5 activator 1**	554.0	interacts with	luteolin (interacts with) CDK5	FALSE	interacts with	luteolin (interacts with) CDK5	
**Cytochrome P450 1A1**	413.21872306	interacts with	luteolin (interacts with) CYP1A1	FALSE	interacts with	luteolin (interacts with) CYP1A1	
**Cytochrome P450 1A2**	383.5908713	interacts with	luteolin (interacts with) CYP1A2	FALSE	interacts with	luteolin (interacts with) CYP1A2	
**Cytochrome P450 1B1**	409.8200966	interacts with	luteolin (interacts with) CYP1B1	FALSE	interacts with	luteolin (interacts with) CYP1B1	
**Cytochrome P450 2C19**	383.5908713	interacts with	luteolin (interacts with) CYP2C19	FALSE	interacts with	luteolin (interacts with) CYP2C19	
**Cytochrome P450 2C9**	383.5908713	interacts with	luteolin (interacts with) CYP2C9	FALSE	interacts with	luteolin (interacts with) CYP2C9	
**DNA polymerase beta**	450.15166178	interacts with	luteolin (interacts with) POLB	FALSE	interacts with	luteolin (interacts with) POLB	
**DNA polymerase eta**	409.8200966	interacts with	luteolin (interacts with) POLH	FALSE	interacts with	luteolin (interacts with) POLH	
**DNA polymerase iota**	409.8200966	interacts with	luteolin (interacts with) POLI	FALSE	interacts with	luteolin (interacts with) POLI	
**DNA polymerase kappa**	442.13683344	interacts with	luteolin (interacts with) POLK	FALSE	interacts with	luteolin (interacts with) POLK	
**DNA-(apurinic or apyrimidinic site) lyase**	442.13683344	interacts with	luteolin (interacts with) APEX1	FALSE	interacts with	luteolin (interacts with) APEX1	
**Endoplasmic reticulum-associated amyloid beta-peptide-binding protein**	383.00761593	interacts with	luteolin (interacts with) HSD17B10	FALSE	interacts with	luteolin (interacts with) HSD17B10	
**Flap endonuclease 1**	409.8200966	interacts with	luteolin (interacts with) FEN1	FALSE	interacts with	luteolin (interacts with) FEN1	
**G-protein coupled receptor 35**	383.5908713	interacts with	luteolin (interacts with) GPR35	FALSE	interacts with	luteolin (interacts with) GPR35	
**Galactokinase**	554.0	interacts with	luteolin (interacts with) GALK1	FALSE	interacts with	luteolin (interacts with) GALK1	
**Geminin**	455.93110514	interacts with	luteolin (interacts with) GMNN	FALSE	interacts with	luteolin (interacts with) GMNN	
**Glutaminase kidney isoform, mitochondrial**	549.09639617	interacts with	luteolin (interacts with) GLS	FALSE	interacts with	luteolin (interacts with) GLS	
**Glyoxalase I**	383.5908713	interacts with	luteolin (interacts with) GLO1	FALSE	interacts with	luteolin (interacts with) GLO1	
**Histone-lysine N-methyltransferase MLL**	383.00761593	interacts with	luteolin (interacts with) KMT2A	FALSE	interacts with	luteolin (interacts with) KMT2A	
**Histone-lysine N-methyltransferase, H3 lysine-9 specific 3**	507.42398964	interacts with	luteolin (interacts with) EHMT2	FALSE	interacts with	luteolin (interacts with) EHMT2	
**Lymphocyte differentiation antigen CD38**	554.0	interacts with	luteolin (interacts with) CD38	FALSE	interacts with	luteolin (interacts with) CD38	
**Lysine-specific demethylase 4A**	562.74626322	interacts with	luteolin (interacts with) KDM4A	FALSE	interacts with	luteolin (interacts with) KDM4A	
**Lysine-specific demethylase 4D-like**	442.13683344	interacts with	luteolin (interacts with) KDM4E	FALSE	interacts with	luteolin (interacts with) KDM4E	
**Lysyl-tRNA synthetase**	554.0	interacts with	luteolin (interacts with) KARS	FALSE	interacts with	luteolin (interacts with) KARS	
**Microtubule-associated protein tau**	383.00761593	interacts with	luteolin (interacts with) MAPT	FALSE	interacts with	luteolin (interacts with) MAPT	
**Mothers against decapentaplegic homolog 3**	554.0	interacts with	luteolin (interacts with) SMAD3	FALSE	interacts with	luteolin (interacts with) SMAD3	
**Muscleblind-like protein 1**	490.88475032	interacts with	luteolin (interacts with) MBNL1	FALSE	interacts with	luteolin (interacts with) MBNL1	
**NADPH oxidase 4**	490.88475032	interacts with	luteolin (interacts with) NOX4	FALSE	interacts with	luteolin (interacts with) NOX4	

**Nuclear factor erythroid 2-related factor 2**	554.0	interacts with	luteolin (interacts with) NFE2L2	FALSE	interacts with	luteolin (interacts with) NFE2L2	
**P-glycoprotein 1**	956.01708614	interacts with	luteolin (interacts with) ABCB1	FALSE	interacts with	luteolin (interacts with) ABCB1	
**Peptidyl-prolyl cis-trans isomerase NIMA-interacting 1**	383.5908713	interacts with	luteolin (interacts with) PIN1	FALSE	interacts with	luteolin (interacts with) PIN1	
**Prelamin-A/C**	450.15166178	interacts with	luteolin (interacts with) LMNA	FALSE	interacts with	luteolin (interacts with) LMNA	
**Runt-related transcription factor 1/Core-binding factor subunit beta**	409.8200966	interacts with	luteolin (interacts with) RUNX1	FALSE	interacts with	luteolin (interacts with) RUNX1	
**Salivary alpha-amylase**	554.0	interacts with	luteolin (interacts with) AMY1A	FALSE	interacts with	luteolin (interacts with) AMY1A	
**Serine/threonine-protein kinase PIM1**	383.5908713	interacts with	luteolin (interacts with) PIM1	FALSE	interacts with	luteolin (interacts with) PIM1	
**Sialidase 2**	1176.07047683	interacts with	luteolin (interacts with) NEU2	FALSE	interacts with	luteolin (interacts with) NEU2	
**Survival motor neuron protein**	409.8200966	interacts with	luteolin (interacts with) SMN1	FALSE	interacts with	luteolin (interacts with) SMN1	
**Tankyrase-1**	413.21872306	interacts with	luteolin (interacts with) TNKS	FALSE	interacts with	luteolin (interacts with) TNKS	
**Tankyrase-2**	413.21872306	interacts with	luteolin (interacts with) TNKS2	FALSE	interacts with	luteolin (interacts with) TNKS2	
**Thyroid hormone receptor beta-1**	383.00761593	interacts with	luteolin (interacts with) THRB	FALSE	interacts with	luteolin (interacts with) THRB	
**Trypsin I**	554.0	interacts with	luteolin (interacts with) PRSS1	FALSE	interacts with	luteolin (interacts with) PRSS1	
**Tyrosyl-DNA phosphodiesterase 1**	450.15166178	interacts with	luteolin (interacts with) TDP1	FALSE	interacts with	luteolin (interacts with) TDP1	
**Ubiquitin carboxyl-terminal hydrolase 1**	554.0	interacts with	luteolin (interacts with) USP1	FALSE	interacts with	luteolin (interacts with) USP1	
**Werner syndrome ATP-dependent helicase**	383.5908713	interacts with	luteolin (interacts with) WRN	FALSE	interacts with	luteolin (interacts with) WRN	
**Arachidonate 5-lipoxygenase**	449.22990244	interacts with	luteolin (interacts with) ALOX5	FALSE	interacts with	luteolin (interacts with) ALOX5	
**Lysine-specific demethylase 4A**	562.74626322	interacts with	luteolin (interacts with) KDM4A	FALSE	interacts with	luteolin (interacts with) KDM4A	
**Aldose reductase**	1717.74921557	interacts with	luteolin (interacts with) AKR1B1	FALSE	interacts with	luteolin (interacts with) AKR1B1	
**PI3-kinase p85-alpha subunit**	490.88475032	interacts with	luteolin (interacts with) PIK3R1	FALSE	interacts with	luteolin (interacts with) PIK3R1	
**Cytochrome P450 1A1**	413.21872306	interacts with	luteolin (interacts with) CYP1A1	FALSE	interacts with	luteolin (interacts with) CYP1A1	
**Nuclear receptor subfamily 0 group B member 1**	493.84586066	interacts with	luteolin (interacts with) NR0B1	FALSE	interacts with	luteolin (interacts with) NR0B1	
**NADPH oxidase 4**	490.88475032	interacts with	luteolin (interacts with) NOX4	FALSE	interacts with	luteolin (interacts with) NOX4	
**Cytochrome P450 1B1**	409.8200966	interacts with	luteolin (interacts with) CYP1B1	FALSE	interacts with	luteolin (interacts with) CYP1B1	
**Lysine-specific demethylase 4C**	1021.41653271	interacts with	luteolin (interacts with) KDM4C	FALSE	interacts with	luteolin (interacts with) KDM4C	
**GABA-A receptor; anion channel**	745.34629183	interacts with	luteolin (interacts with) GABRG2	FALSE	interacts with	luteolin (interacts with) GABRG2	
**Intestinal alkaline phosphatase**	935.10915368	interacts with	luteolin (interacts with) ALPI	FALSE	interacts with	luteolin (interacts with) ALPI	
**Xanthine dehydrogenase**	1083.45359247	interacts with	luteolin (interacts with) XDH	FALSE	interacts with	luteolin (interacts with) XDH	
**Aldehyde reductase**	490.88475032	interacts with	luteolin (interacts with) AKR1A1	FALSE	interacts with	luteolin (interacts with) AKR1A1	
**Arachidonate 15-lipoxygenase**	383.5908713	interacts with	luteolin (interacts with) ALOX15	FALSE	interacts with	luteolin (interacts with) ALOX15	
**Carbonic anhydrase V**	808.78798375	interacts with	luteolin (interacts with) CA5A	FALSE	interacts with	luteolin (interacts with) CA5A	
**5'-nucleotidase**	490.88475032	interacts with	luteolin (interacts with) NT5E	FALSE	interacts with	luteolin (interacts with) NT5E	
**CaM kinase II**	490.88475032	interacts with	luteolin (interacts with) CAMK2B	FALSE	interacts with	luteolin (interacts with) CAMK2B	
**Sentrin-specific protease 1**	486.07642711	interacts with	luteolin (interacts with) SENP1	FALSE	interacts with	luteolin (interacts with) SENP1	
**Carbonic anhydrase**	908.57890855	interacts with	luteolin (interacts with) CA3	FALSE	interacts with	luteolin (interacts with) CA3	
**Carbonic anhydrase VI**	1044.998295	interacts with	luteolin (interacts with) CA6	FALSE	interacts with	luteolin (interacts with) CA6	
**Multidrug resistance-associated protein 1**	554.0	interacts with	luteolin (interacts with) ABCC1	FALSE	interacts with	luteolin (interacts with) ABCC1	
**Arachidonate 12-lipoxygenase**	409.8200966	interacts with	luteolin (interacts with) ALOX12	FALSE	interacts with	luteolin (interacts with) ALOX12	
**ATP-binding cassette sub-family G member 2**	586.77944647	interacts with	luteolin (interacts with) ABCG2	FALSE	interacts with	luteolin (interacts with) ABCG2	
**Telomerase reverse transcriptase**	554.0	interacts with	luteolin (interacts with) TERT	FALSE	interacts with	luteolin (interacts with) TERT	
**Glyoxalase I**	383.5908713	interacts with	luteolin (interacts with) GLO1	FALSE	interacts with	luteolin (interacts with) GLO1	
